# Nano‐Confined Radical Anion as An NIR‐II Photothermal Immunogenic Amplifier for In Situ Cancer Vaccination

**DOI:** 10.1002/advs.76277

**Published:** 2026-06-26

**Authors:** Mengxin Mu, Mengyu Guo, Jun Guan, Xiaofeng He, Mingdi Hu, Fene Gao, Klaus Müllen, Chendong Ji, Chunying Chen, Meizhen Yin

**Affiliations:** ^1^ State Key Laboratory of Chemical Resource Engineering Beijing University of Chemical Technology Beijing China; ^2^ CAS Key Laboratory for Biomedical Effects of Nanomaterials and Nanosafety & CAS Center for Excellence in Nanoscience National Center for Nanoscience and Technology of China Beijing China; ^3^ Max Planck Institute for Polymer Research Mainz Germany

**Keywords:** D‐Chiral hydrogel, glutathione depletion, organic radical anions, photothermal immunotherapy, terrylene diimide

## Abstract

Immunogenic cell death (ICD) induced by photothermal therapy holds promise for eliciting systemic antitumor immunity; however, its efficacy is frequently limited by tumor‐intrinsic immunosuppressive defenses. Herein, we report a nano‐confined radical anion as a photothermal immunogenic amplifier that exploits kinetic stabilization to couple high NIR‐II photothermal efficiency with active glutathione depletion for robust in situ cancer vaccination. This amplifier is constructed through the simple co‐assembly of terrylene diimide (TDI) with Pluronic F127, forming nanoparticles in which stable TDI radical anions are generated and kinetically stabilized. The radical anion nanoparticles are further integrated into an injectable hydrogel to create a multifunctional in situ vaccine. This platform ensures prolonged tumor retention, captures tumor‐associated antigens released during ICD, and provides intrinsic adjuvanticity. In murine models, local laser activation of the platform ablates primary tumors and elicits systemic antitumor immune responses associated with suppression of distant tumor growth. The stabilization of organic radical anions by molecular and supramolecular design combines local photothermal therapy with systemic anti‐tumor immunity.

## Graphical abstract

A nano‐confined radical anion acts as a photothermal immunogenic amplifier. Kinetically stabilized nanoparticles couple high near‐infrared‐II (NIR‐II) photothermal efficacy with glutathione (GSH) depletion to boost reactive oxygen species (ROS) and immunogenic cell death (ICD). Integrated into a D‐chiral hydrogel for prolonged retention, this platform functions as an in situ vaccine, eliciting robust systemic anti‐tumor immunity.

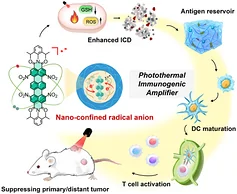

## 1 Introduction

Immunogenic cell death (ICD), a regulated form of cell death, releases damage‐associated molecular patterns and tumor‐associated antigens (TAAs) that prime an adaptive immune response [1]. Inducing ICD to transform a tumor into an in situ vaccine is a compelling strategy for eliciting systemic anti‐tumor immunity [2, 3, 4]. Among various ICD inducers, photothermal therapy (PTT), which uses photothermal agents to convert light into heat and cause cancer cell death, is particularly attractive for its high spatiotemporal precision. Emerging evidence has redefined the immunogenic potential of PTT as being fundamentally governed by the induction of profound oxidative stress, a process mediated by a burst of reactive oxygen species (ROS) that triggers the requisite endoplasmic reticulum stress [5, 6]. To potentiate this ROS‐mediated effect, PTT is often combined with external ROS‐generators like photosensitizers or chemodrugs. However, these may reintroduce systemic toxicities and risk provoking chronic, immunosuppressive inflammation. A more elegant strategy is to target an internal ROS‐generation pathway. The high intracellular concentration of glutathione (GSH), the tumor's primary antioxidant shield against ROS, represents an ideal target. Depleting GSH directly lowers the threshold for oxidative damage, amplifying photothermal‐induced immunogenic signal [7, 8]. This necessitates a photothermal immunogenic amplifier to simultaneously generate heat and dismantle the tumor's intrinsic antioxidant defenses.

Organic conjugated radical anions, characterized by their unpaired electrons and strong spin‐coupling, possess an electronic structure that confers both efficient near‐infrared (NIR) photothermal conversion and pronounced redox activity [9]. Nevertheless, their effective generation and controlled conversion against quenching to closed‐shell forms in aqueous physiological environments remain a formidable challenge. Rylene carboximide stands for an important class of chromophores made by a combination of peri‐fused naphthalenes and dicarboxamide auxochromic groups, such as perylene diimide (PDI) and terrylene diimide (TDI). They possess high molar extinction coefficients and finely tunable photophysical properties [10]. Radical anions derived from PDI have garnered significant interest for PTT, typically achieving a photothermal conversion efficiency (PCE) up to 58% under 808 nm (NIR‐I) laser irradiation [11, 12]. Current strategies to stabilize these PDI radical anions primarily focus on suppressing radical dimerization through steric hindrance, achieved by employing bulky substituents or supramolecular encapsulation [13, 14]. However, these systems rely on moderate‐to‐high reducing environments for radical generation and to prevent oxidation back to the neutral state, resulting in limited photophysical performance in vivo. Terrylene diimide (TDI) derivatives offer a promising alternative, possessing extended π‐conjugation and superior electron‐accepting capabilities compared to PDI analogues [15]. These attributes facilitate the formation of TDI‐based radical anions with optical absorptions extending into the second near‐infrared window (NIR‐II, 1000–1700 nm) [16]. This allows for deeper tissue penetration with minimal scattering by endogenous chromophores [17, 18, 19]. The electron‐accepting capability of TDIs can be enhanced by introducing electron‐withdrawing nitro substituents, which further lower the energy of the lowest unoccupied molecular orbital (LUMO) and reduce susceptibility to oxidation [20]. While this modification facilitates the formation of TDI radical anions (TDI^•−^) under milder and biocompatible reducing conditions, it simultaneously permits over‐reduction to diamagnetic dianions (TDI^2^^−^) in the presence of electron donors [21]. The propensity for sequential electron transfer can hypsochromically shift absorption away from the NIR‐II region and impair PCE, hindering reliable biological applications. Therefore, a photothermal immunogenic amplifier requires the biocompatible generation and stabilization of radical anions for efficient NIR‐II photothermal performance, while enabling subsequent reduction by GSH. Encapsulating TDI within nanoparticles using amphiphilic polymers—commonly employed to improve the water dispersibility of photothermal agents—has not previously been explored as a strategy to simultaneously provide a mildly reducing environment and a loosely packed structure that permits environmental GSH to access and react with TDI.

**SCHEME 1:**
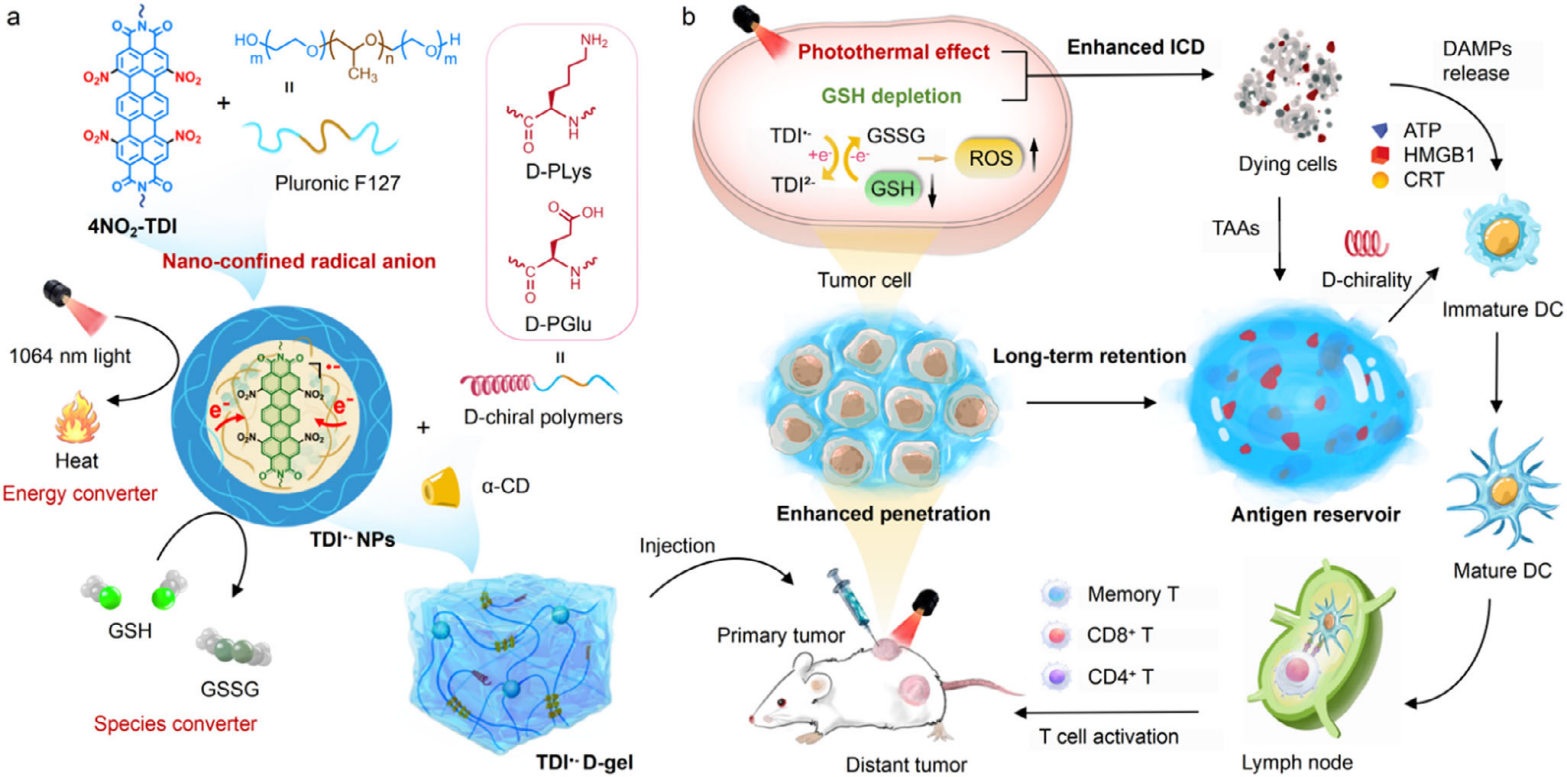
(a) Hierarchical assembly of the TDI^•−^ D‐gel. 4NO_2_‐TDI and Pluronic F127 co‐assemble into radical anion nanoparticles (TDI^•−^ NPs). Then, TDI^•−^ NPs are incorporated into D‐chiral polymers and α‐CD to form a thermoreversible TDI^•−^ D‐gel. (b) Upon 1064 nm laser irradiation, the photothermal effect and GSH depletion stemming from the injectable TDI^•−^ D‐gel synergistically promote ICD and the release of TAAs. The hydrogel then captures these TAAs to promote DC maturation, leading to sustained antitumor immune activation.

Herein, we report the facile construction of a TDI^•−^‐based photothermal immunogenic amplifier, TDI^•−^ NPs, via molecular design and facile supramolecular assembly. Co‐assembly of the tetranitro‐substituted TDI (4NO_2_‐TDI) with the amphiphilic polymer Pluronic F127 forms nanoparticles (TDI^•−^ NPs), wherein the polymer not only imparts aqueous solubility to 4NO_2_‐TDI but also simultaneously reduces it into TDI^•−^ and stabilizes it through the nano‐confined environment. TDI^•−^ NPs exhibit high stability against both oxidative quenching and over‐reduction for up to two months under ambient conditions, and a high NIR‐II PCE of 60.8%. Importantly, the encapsulated TDI^•−^ within F127 retains its reactivity towards intratumoral GSH, enabling a cascade of GSH depletion, ROS amplification, and a potent ICD effect. To translate the local ICD into a robust systemic anti‐tumor immunity, we integrated the TDI^•−^ NPs into an injectable hydrogel (TDI^•−^ D‐gel) composed of D‐chiral poly(amino acids) and α‐cyclodextrin (α‐CD) (Scheme 1a). TDI^•−^ D‐gel prolongs tumor retention, functions as an intrinsic adjuvant promoting dendritic cell (DC) maturation, and serves as an antigen reservoir for in situ vaccination. In murine tumor models, 1064 nm laser activation of TDI^•−^ D‐gel achieved effective primary tumor ablation and elicited a systemic immune response that was associated with suppression of distant tumor growth (Scheme 1b).

## 2 Results and Discussion

### 2.1 Electron‐Accepting Property of 4NO2‐TDI

**FIGURE 1:**
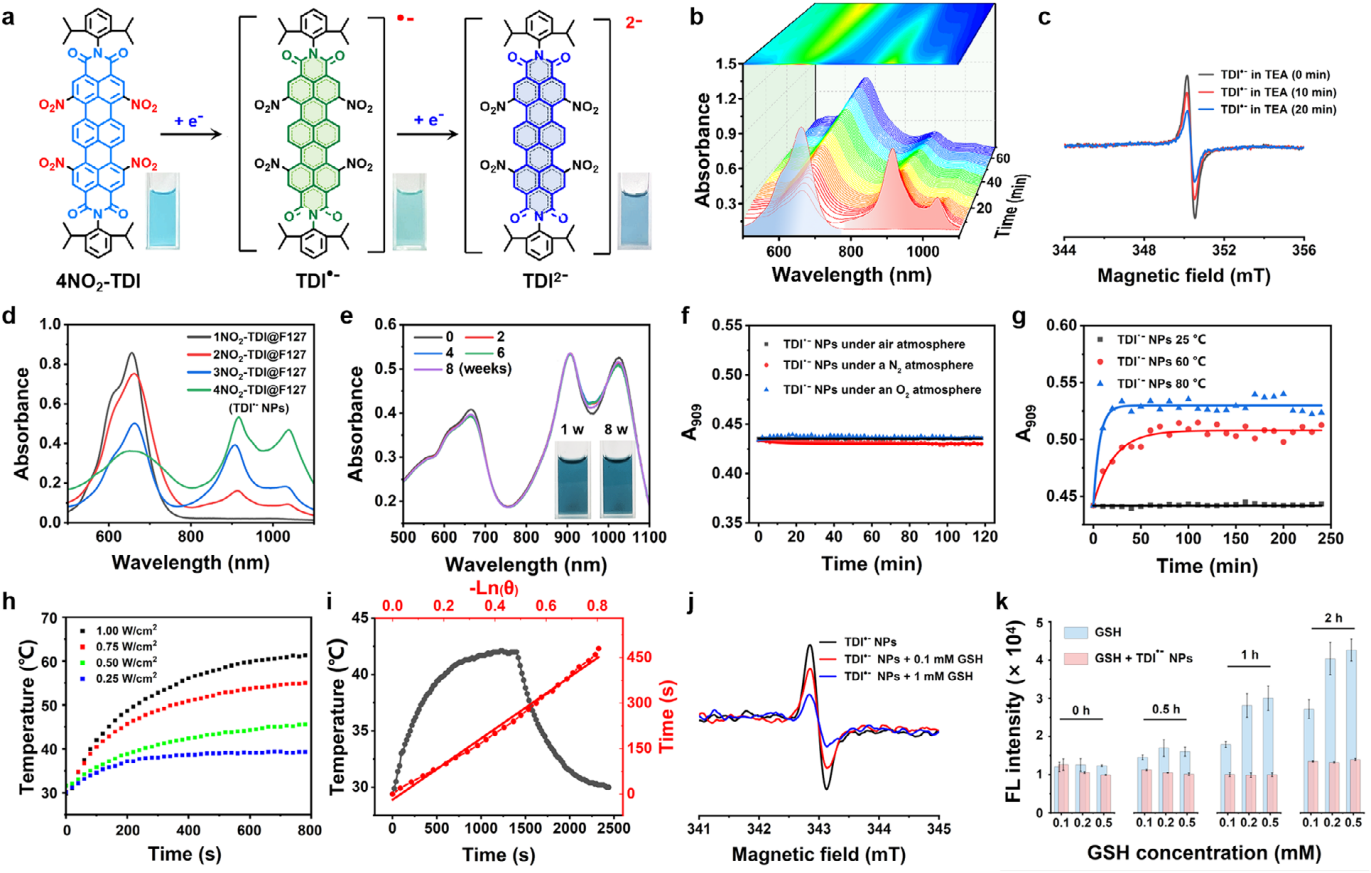
(a) Scheme of 4NO_2_‐TDI radical anion (TDI^•−^) and dianion (TDI^2−^) generation via electron transfer. (b) Spectroscopic monitoring of 4NO_2_‐TDI in tetrahydrofuran (THF) in the presence of triethylamine (TEA), showing the formation of TDI^•−^ and TDI^2−^. (c) Electron paramagnetic resonance (EPR) intensity changes of 4NO_2_‐TDI at different time points after the addition of TEA. (d) Absorption spectra of 1–4NO_2_‐TDI@F127 nanoparticles in aqueous solution. (e) Absorbance changes of TDI^•−^ NPs over 8 weeks, with inset photos showing the solution at initial and 8 weeks under storage. (f) Absorption peak at 909 nm (A_909_) of TDI^•−^ NPs in the presence of N_2_ or O_2_ and (g) at different temperatures. (h) Temperature profiles of TDI^•−^ NPs under 1064 nm laser irradiation at different power densities. (i) Heating and cooling curves of TDI^•−^ NPs under 1064 nm laser irradiation (0.5 W/cm^2^). (j) EPR intensity changes of TDI^•−^ NPs after adding different concentrations of GSH. (k) Fluorescence intensity of monochlorobimane (MCB) probe over time when incubated with GSH, with or without TDI^•−^ NPs. Data are presented as mean ± SD (n = 4). Statistical significance was calculated via one‐way ANOVA with Tukey's post hoc test. P < 0.05 was considered statistically significant.

A series of TDI derivatives with one to four bay‐substituted nitro groups (1‐4NO_2_‐TDI) were synthesized and characterized by ^1^H NMR, ^13^C NMR, and mass spectrometry (Scheme S1 and Figures S1–S12). Density functional theory (DFT) calculations indicated that the introduction of these electron‐withdrawing groups progressively lowered the LUMO energy level, making 4NO_2_‐TDI the most potent electron acceptor. Cyclic voltammetry (CV) revealed two distinct reduction peaks for all substituted TDIs, with 4NO_2_‐TDI showing the most positive reduction potentials at ‐0.03 and 0.13 V, significantly higher than those of PDIs [22] (Figures S13, S14 and Table S1). The sequential two‐step reduction of 4NO_2_‐TDI to its radical anion (TDI^•−^) and then to the dianion (TDI^2−^) was tracked in tetrahydrofuran (THF) using triethylamine (TEA) as a model electron donor (Figure 1a). UV–vis spectroscopic monitoring revealed the transient appearance of the TDI^•−^ absorption peak at 909 nm, followed by the conversion of TDI^•−^ to TDI^2−^ as evidenced by the emergence of the absorption peak at 680 nm. (Figure 1b, Figures S15 and S16a). Electron paramagnetic resonance (EPR) spectroscopy confirmed a strong paramagnetic signal upon TEA addition, which decayed to half of its original intensity over 20 min (Figure 1c). A similar evolution in both UV‐vis and EPR spectra was observed upon treatment with the biological antioxidant GSH (Figure S16b).

The potent electron‐accepting nature of 4NO_2_‐TDI enables radical anion formation by biocompatible molecules, whose electron‐donating abilities are typically too weak to qualify conventional reducing agents. Pluronic F127, an amphiphilic triblock copolymer composed of a central polypropylene oxide (PPO) block flanked by polyethylene oxide (PEO) chains, was employed as electron donor. When mixed with 4NO_2_‐TDI in THF, F127 also mediated a two‐step reduction, although at a slower rate than an equivalent amount of TEA or GSH (Figure S16c). Although 2NO_2_‐TDI and 3NO_2_‐TDI also showed time‐dependent radical‐anion formation in the presence of GSH or F127 in solution (Figure S17), their overall spectral responses were less favorable than those of 4NO_2_‐TDI. In solution, efficient diffusion and molecular contact enable sequential electron transfer, and the good dispersion of TDI^2^^−^ minimizes electrostatic repulsion, together facilitating the undesired over‐reduction of TDI^•−^. In contrast, confining 4NO_2_‐TDI through nanoparticle assembly appears to “freeze” the intermediate state, favoring the selective formation and stabilization of TDI^•−^ (vide infra).

### 2.2 Photothermal and Redox‐Modulating Performance of TDI•− NPs

Rapid injection of a THF solution of 4NO_2_‐TDI and F127 into water yielded uniform nanoparticles with an average diameter of ≈48 nm. The co‐assembled nanoparticles, henceforth termed TDI^•−^ NPs, exhibited a persistent radical anion absorption peak at 909 nm in aqueous media (Figure 1d and Figure S18). Benefiting from the nano‐confined microenvironment, these radical anions show remarkable stability, maintaining their radical anion absorption for over two months under ambient conditions (Figure 1e) and in both nitrogen and oxygen atmospheres (Figure 1f and Figure S19). Notably, the radical absorption intensity increases at elevated temperatures (up to 80 °C), a behavior opposite to that of previously reported PDI^•−^, which suffer from significant thermal quenching [24] (Figure 1g and Figure S20). The increase in radical absorption intensity at elevated temperature may be associated with thermal relaxation of the nano‐confined F127 microenvironment. Increased local chain mobility at higher temperature could reduce constraints on charge separation and favor the persistence of the radical‐anion state, thereby leading to a more pronounced radical absorption signal.

Through screening various combinations of TDI derivatives and amphiphilic polymers, 4NO_2_‐TDI and F127 were identified as the optimal pair, as co‐assembly with other polymers or with TDI derivatives possessing lower electron‐accepting abilities (1‐3NO_2_‐TDI) led to weaker radical signals (Figure 1d and Figure S21). The NIR‐II photothermal performance of the TDI^•−^ NPs was investigated. Upon irradiation with a 1064 nm laser, aqueous TDI^•−^ NPs exhibited a rapid, power density‐dependent temperature increase (Figure 1h). The PCE was calculated to be 60.8% (Figure 1i), substantially surpassing most of the reported photothermal organic radicals [12, 14]. The nanoparticles also demonstrated excellent photostability, with their absorption spectrum remaining unchanged after multiple irradiation‐cooling cycles (Figure S22). Pronounced thermal stability was also seen in solid state, where the lyophilized powder could be heated above 150 °C under 1064 nm laser irradiation without radical quenching (Figure S23).

Different electron donors such as Na_2_S_2_O_4_, TEA, and GSH were individually added, each resulting in the conversion of the radical anion to the dianion, as evidenced by characteristic absorption changes (Figure S24a). Given the therapeutic interest of GSH [23, 24, 25, 26], the concentration‐dependent reactivity was examined, with higher GSH concentrations leading to increased yields of dianions (Figure S24b–d). This radical‐to‐dianion conversion was also validated by the decay of the TDI^•−^ EPR signal (Figure 1j). The consumption of GSH by TDI^•−^ NPs was monitored using the monochlorobimane (MCB) fluorescence probe [27]. In control groups, GSH enhanced the fluorescence of MCB over time, while in the presence of TDI^•−^ NPs, the fluorescence remained low, indicating that GSH was effectively consumed (Figure 1k). These results revealed the redox reaction between TDI^•−^ NPs and GSH (Scheme S2), highlighting the dual functionality of TDI^•−^ NPs for both potent NIR‐II PTT and tumor microenvironment (TME) modulation.

### 2.3 Injectable D‐Chiral Hydrogel Based on TDI•− NPs

The hydrogel was designed as an integrated system in which F127 supports TDI^•−^ nanoparticle formation and stabilization, D‐chiral poly(amino acids) enhance persistence and retention, and α‐CD induces supramolecular gelation. This combination enables local delivery, redox activity, and reservoir‐like retention within one platform. Translating local PTT into a robust systemic anti‐tumor immune response in vivo requires overcoming critical delivery barriers, such as short nanoparticle retention time at the tumor site and rapid clearance of TAAs released during PTT‐induced ICD [28, 29, 30, 31, 32]. Clearly, an in situ antigen reservoir capturing TAAs and enabling sustained release is essential for effective in situ vaccination. Therefore, we embed TDI^•−^ NPs into an injectable, sol‐gel transition reversible hydrogel (TDI^•−^ D‐gel) toward a photothermally activatable in situ vaccine. During laser irradiation, the photothermally induced sol state enhances the spatial distribution of TDI^•−^ NPs at the tumor site and facilitates their reaction with surrounding GSH, promoting ICD and TAA release. Upon cooling, reversion to a solid‐gel phase enhances the function of the hydrogel as an antigen reservoir by trapping the released TAAs.

**FIGURE 2:**
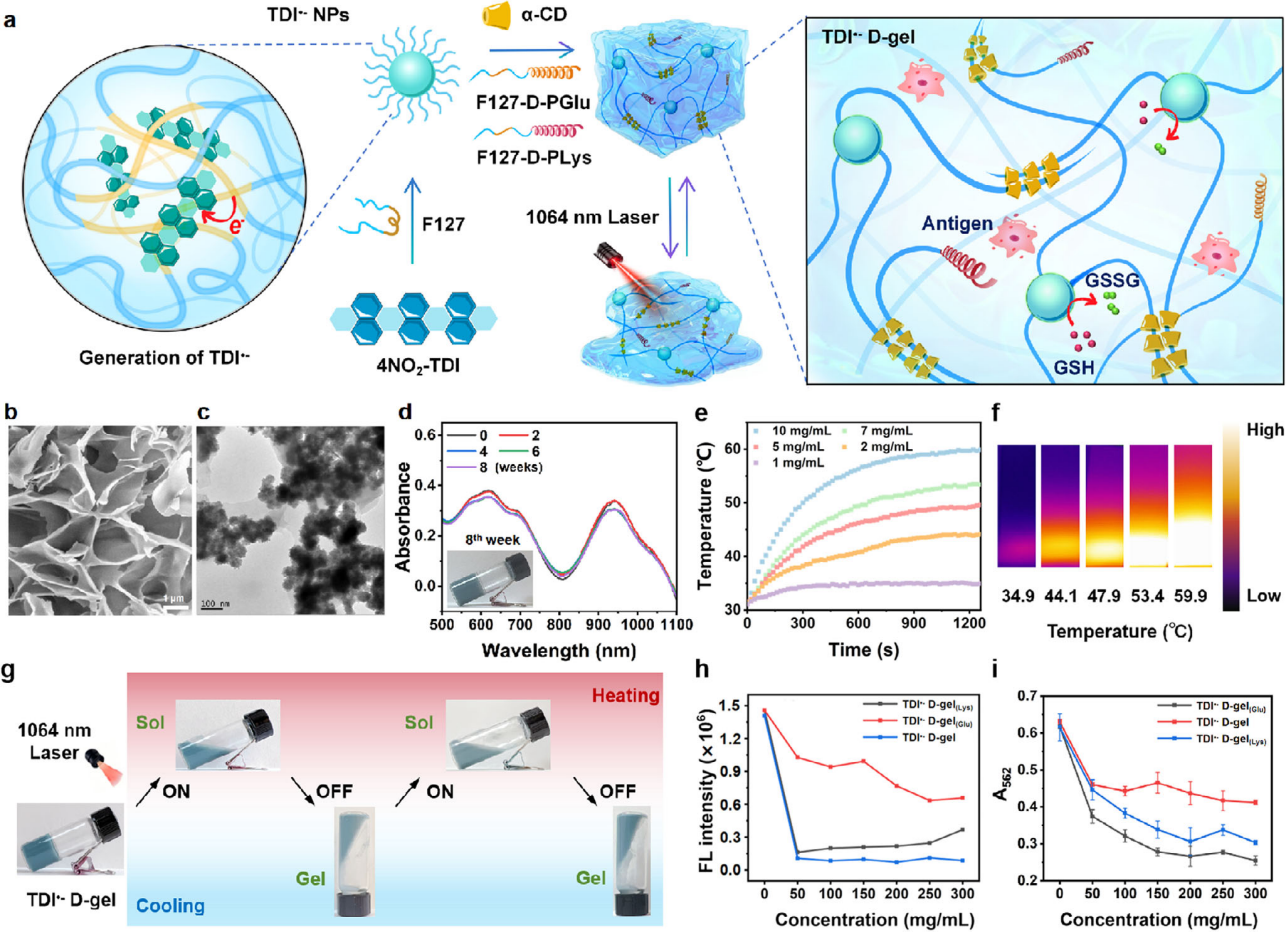
(a) Schematic illustration of the TDI^•−^ D‐gel composition and internal electron transfer. (b and c) Scanning electron microscopy (SEM, scale bar: 1 µm.) and transmission electron microscopy (TEM, scale bar: 100 nm) images of the TDI^•−^ D‐gel. (d) UV–vis absorption spectra of the TDI^•−^ D‐gel after storage at room temperature for different time periods (0–8 weeks). Inset: Photograph of the gel at week 8. Heating curves (e) and photothermal images (f) of the TDI^•−^ D‐gel with different concentrations (1‐10 mg/mL) under 1064 nm laser irradiation (0.5 W/cm^2^). (g) Photographs showing gel‐sol phase transition after multiple laser irradiation cycles. (h) Fluorescence spectra of different concentrations (0–300 mg/mL) of the TDI^•−^ D‐gel_(Lys)_ TDI^•−^ D‐gel_(Glu)_ and TDI^•−^ D‐gel after incubation with fluorescein isothiocyanate‐labeled bovine serum albumin (FITC‐BSA). (i) Absorption values at 532 nm of different concentrations (0–300 mg/mL) of the TDI^•−^ D‐gel_(Lys)_, TDI^•−^ D‐gel_(Glu)_ and TDI^•−^ D‐gel after incubation with TAAs using BCA protein assay kit. Data are presented as mean ± SD (n = 4). Statistical significance was calculated via one‐way ANOVA with Tukey's post hoc test. P < 0.05 was considered statistically significant.

The D‐chiral poly(amino acid) constituents of the hydrogel ensure prolonged local retention via enzymatic degradation resistance [33] and provide intrinsic adjuvant effects for enhanced immune response [34, 35, 36]. TDI^•−^ D‐gel was prepared by mixing TDI^•−^ NPs with phosphate buffered saline (PBS) solutions of the custom F127‐grafted D‐chiral poly(glutamic acid) and poly(lysine), (D‐PGlu and D‐PLys), followed by the addition of α‐CD to induce gelation (Figure 2a, Figures S25 and S26). L‐chiral analogues were also synthesized as controls. Polymer synthesis and characterization details are provided in the Supporting Information (Table S2, Scheme S3 and Figures S27–S34). Gelation is driven by host‐guest interactions, where F127 polymer chains thread through α‐CD cavities, forming pseudopolyrotaxanes [37, 38]. Scanning electron microscopy (SEM) and transmission electron microscopy (TEM) revealed a porous, interconnected network characteristic of hydrogels (Figure 2b,c). Importantly, incorporation into the D‐chiral hydrogel matrix did not compromise the key functional attributes of TDI^•−^ NPs. The TDI^•−^ D‐gel retained strong NIR‐II absorption (Figure S35), and the encapsulated radical anions remained stable within the hydrogel for over two months (Figure 2d).

Besides, TDI^•−^ D‐gel retained potent photothermal efficacy, exhibiting rapid temperature and stable performance over multiple irradiation cycles under 1064 nm laser exposure (Figure 2e,f and Figure S36). Due to the weak driving force for gelation of pseudopolyrotaxanes, the hydrogel displays a laser‐triggered, reversible sol‐gel phase transition, causing a liquid‐like sol state upon heating and reverting to gel upon cooling (Figure 2g).

To assess its capability for antigen capture, the TDI^•−^ D‐gel was evaluated using a model protein, fluorescein isothiocyanate‐labeled bovine serum albumin (FITC‐BSA). While FITC‐BSA provides a convenient model for comparing protein adsorption behavior, it does not fully reproduce the compositional complexity or immunological properties of endogenous tumor‐associated antigens. For comparison, several control gels with different polymer compositions were prepared using a similar method (Table S3). The hydrogels containing positively charged polylysine (TDI^•−^ D‐gel and TDI^•−^ D/L‐gel_(Lys)_) exhibited significantly enhanced FITC‐BSA adsorption compared to their negatively charged (TDI^•−^ D/L‐gel_(Glu)_) or neutral counterparts (Figure 2h and Figure S37). In Figure 2h, residual FITC‐BSA in the supernatant was quantified by fluorescence, such that lower fluorescence indicates stronger adsorption by the hydrogel. This result confirms that electrostatic interactions are crucial for efficient protein capture. The chirality of the poly(amino acid) did not significantly influence adsorption efficiency (Figure S38). Next, these findings were validated using native TAAs from 4T1 breast cancer cell lysates. Enhanced TAA adsorption by the D‐polylysine‐modified hydrogel was confirmed through both a significant decrease in Zeta potential and quantification via a BCA protein assay (Figure 2i, Figures S39 and S40). In Figure 2i, the hydrogel‐retained protein fraction from 4T1‐derived lysates was quantified by BCA assay, so higher absorbance indicates stronger protein retention. The BCA assay revealed an adsorption capacity of 0.045 mg of TAAs for a 300 mg/mL TDI^•−^ D‐gel, which was 1.8‐fold higher than that of the negatively charged control, TDI^•−^ D‐gel_(Glu)_. These results support the protein‐retention capacity of the hydrogel and its potential role as a local antigen reservoir. The porous and interconnected hydrogel network, together with electrostatic interactions, may facilitate the trapping and retention of released TAAs, thereby prolonging their local availability.

### 2.4 Cellular GSH Depletion and Immune Activation of the TDI•− D‐Gel

**FIGURE 3:**
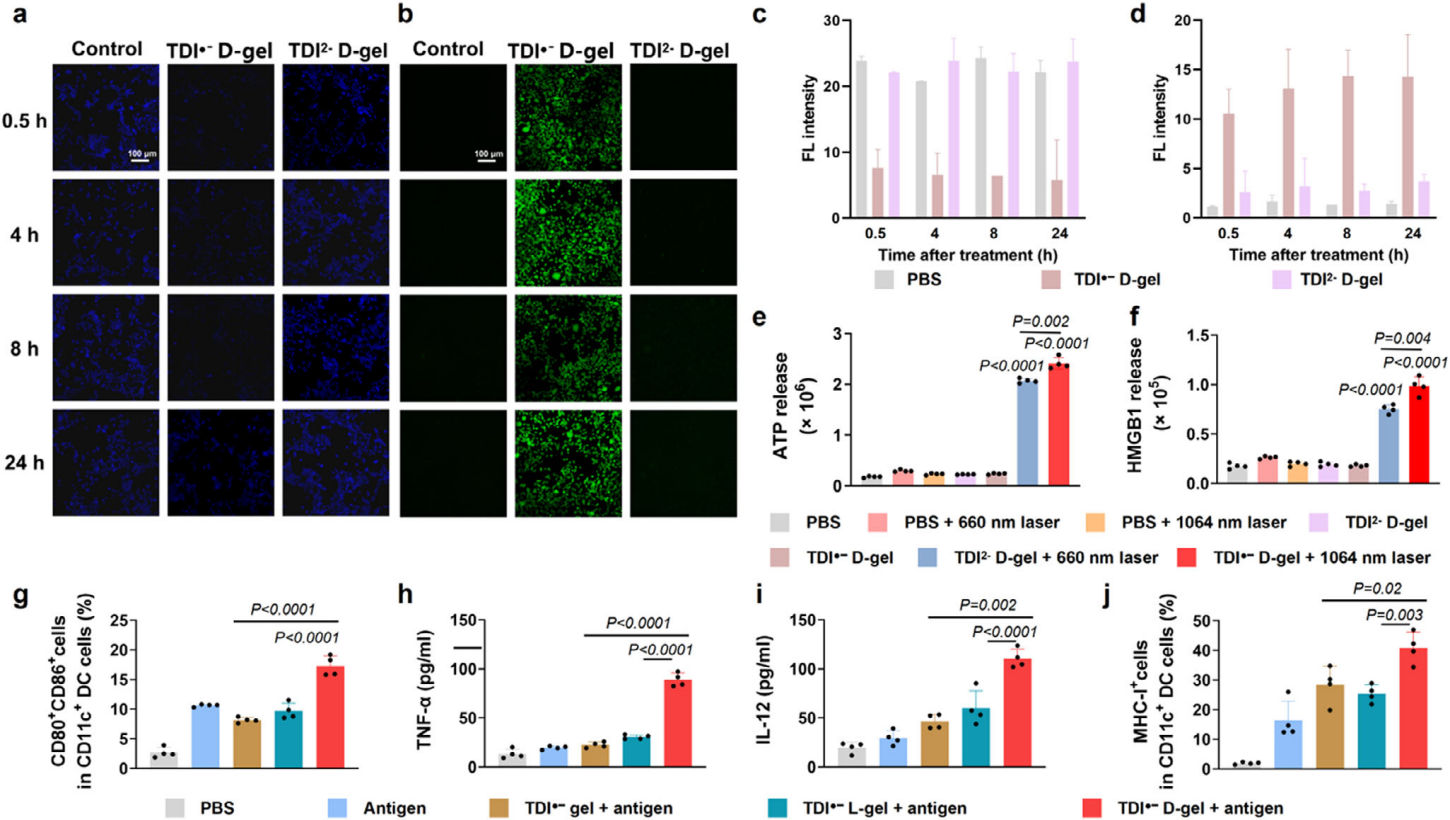
(a, b) Time‐dependent fluorescence images of intracellular GSH depletion and ROS generation in 4T1 cells treated with TDI^•−^ D‐gel or the redox‐inactive control TDI^2−^ D‐gel over 24 h. Scale bar: 100 µm. (c, d) Quantitative analysis of GSH and ROS levels from (a, b). (e) Extracellular adenosine triphosphate (ATP) and (f) high mobility group box 1 (HMGB1) release of 4T1 cells following various treatment conditions. (g) Flow cytometry plots of CD80/CD86 expression on DCs. (h) TNF‐α expression levels. (i) IL‐12 expression levels. (j) MHC‐I expression levels on DC population. Data are presented as mean ± SD (n = 4). Statistical significance was calculated via one‐way ANOVA with Tukey's post hoc test. P < 0.05 was considered statistically significant.

The TDI^•−^ D‐gel was engineered to enhance anti‐tumor immune responses through two distinct but complementary mechanisms. First, the TDI^•−^ species depletes intracellular GSH within the TME, leading to elevated oxidative stress and enhancement of ICD. Second, the D‐chiral amino acid components in the hydrogel drive adjuvant effects that promote DC maturation and inflammatory responses. Fluorescence imaging of 4T1 cancer cells treated with TDI^•−^ D‐gel or the redox‐inactive control TDI^2^^−^ D‐gel showed that the TDI^•−^ D‐gel induced stronger intracellular GSH depletion and ROS generation over 24 h (Figure 3a–d). These results support that the radical‐anion‐containing hydrogel retains intracellular redox‐modulating activity and induces stronger oxidative stress than the redox‐inactive control. The corresponding data for the non‐gelled NP formulation are shown in Figure S41. To validate the synergistic effect of GSH depletion on PTT, a TDI^2^^−^ D‐gel was prepared using GSH‐pretreated TDI^•−^ NPs, serving as a redox‐inactive photothermal control. Because TDI^2^^−^ and TDI^•−^ exhibit different optical absorption characteristics, the TDI^2^^−^ D‐gel and TDI^•−^ D‐gel were irradiated at 660 and 1064 nm, respectively, with laser parameters adjusted to achieve comparable temperature elevation (Figure S42). Compared to control groups, the TDI^•−^ D‐gel with 1064 nm laser irradiation showed enhanced release of adenosine triphosphate (ATP) and high mobility group box 1 (HMGB1), established markers of ICD [39, 40] (Figure 3e,f). This confirmed that elevated ROS levels amplify pro‐inflammatory ICD cascades through oxidative stress‐mediated cellular damage. Because TDI^•−^ and TDI^2^^−^ possess different absorption profiles, this comparison was intended as a functional redox‐state comparison under approximately matched thermal output rather than as a strictly wavelength‐matched optical control. Despite its radical anion‐mediated GSH depletion effects, excellent biocompatibility was observed with the TDI^•−^ D‐gel in both cancer and normal cells under physiological conditions (Figure S43a), while potent cytotoxicity against cancer cells was demonstrated upon NIR‐II laser exposure (Figure S43b and c).

The immunostimulatory function of the TDI^•−^ D‐gel was evaluated on bone marrow‐derived DCs (BMDCs). Flow cytometry analysis revealed significantly enhanced expression of costimulatory molecules CD80/CD86 on DCs when co‐cultured with antigens released from 4T1 cells treated by TDI^•−^ D‐gel plus laser (Figure 3g). This heightened DC activation was accompanied by increased secretion of pro‐inflammatory cytokines, including TNF‐α and IL‐12 [41, 42] (Figure 3h,i). The benefit of the D‐chiral scaffold was highlighted by significantly higher levels of expression of major histocompatibility complex class‐I (MHC‐I) on DCs treated with TDI^•−^ D‐gel compared to an equivalent L‐chiral hydrogel formulation (TDI^•−^ L‐gel + antigen) or antigen alone (Figure 3j). This enhanced antigen presentation capability can be attributed to the prolonged persistence and direct adjuvant effects of the D‐chiral components. These results demonstrate that the TDI^•−^ D‐gel effectively combines redox modulation with immune activation.

### 2.5 In Vivo Retention and Antigen Release by the TDI•− D‐Gel

D‐amino acid polymers were intended to enhance the resistance of the hydrogel against enzymatic degradation and to extend retention time. Preliminary in vivo assessments of different hydrogel formulations were performed via subcutaneous injection in healthy mice to check degradation patterns. Notably, the TDI^•−^ D‐gel exhibited exceptional biostability with in vivo persistence exceeding 11 days post‐injection and maintaining visible structural integrity. In contrast, both its L‐chiral enantiomer (TDI^•−^ L‐gel) and an achiral analogue (TDI^•−^ gel) completely degraded within 7–9 days (Figure S44).

**FIGURE 4:**
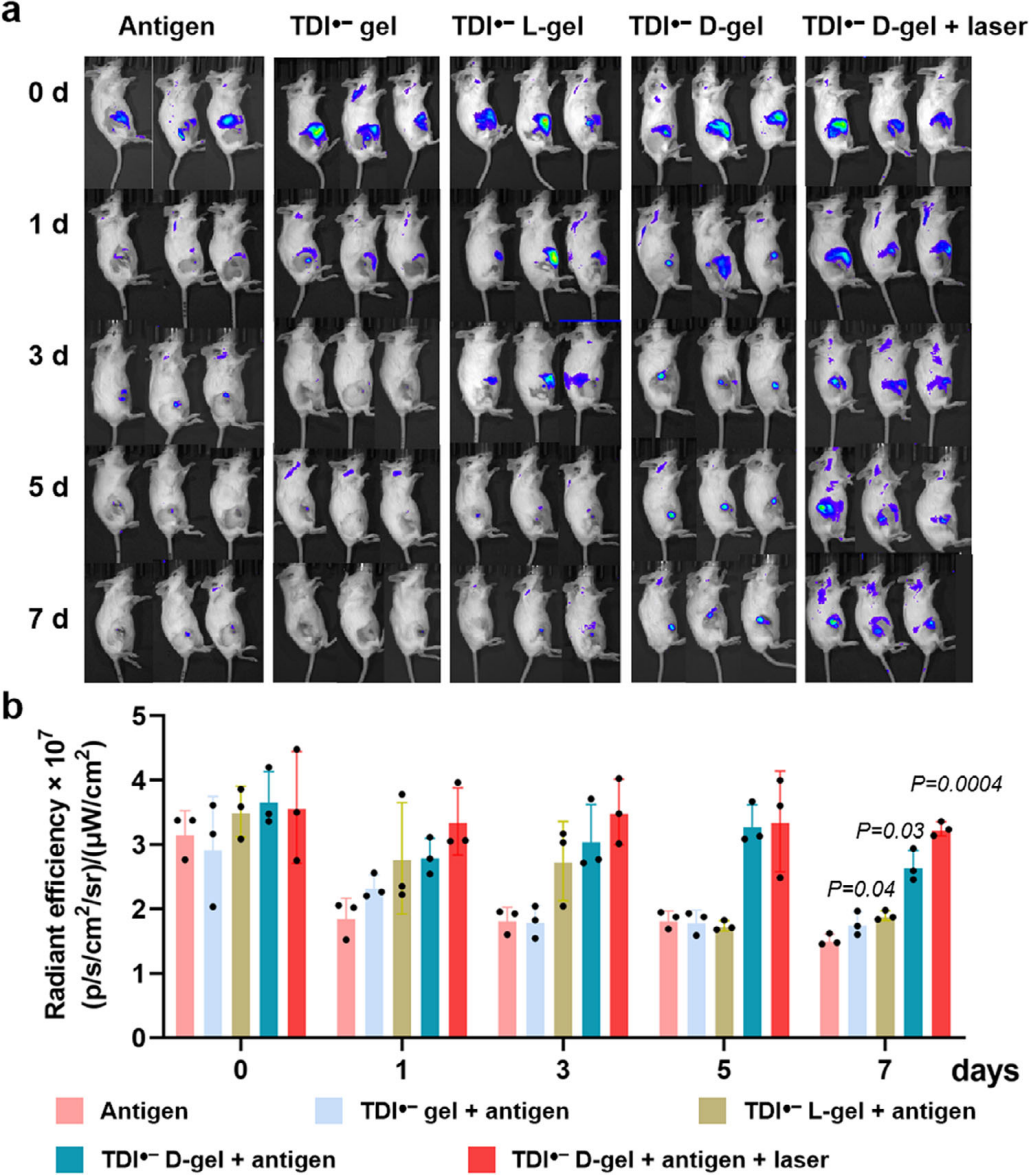
(a) In vivo fluorescence images of ICG‐labeled BSA retention and distribution in different treatment groups over 7 days. (b) Quantitative analysis of fluorescence intensity from (a). Data are presented as mean ± SD (n = 3). Statistical significance was calculated via one‐way ANOVA with Tukey's post hoc test. P < 0.05 was considered statistically significant.

To investigate the antigen retention and spatiotemporal release profiles, we employed NIR fluorescence tracking using Indocyanine Green (ICG)‐labeled BSA as a model antigen [43] (Figure 4a). Quantitative analysis of fluorescence intensity over 7 days demonstrated that the TDI^•−^ D‐gel achieved significantly prolonged antigen retention compared to free antigen and TDI^•−^ L‐gel groups (Figure 4b). Strikingly, the TDI^•−^ D‐gel group maintained approximately 90% of the initial antigen‐associated fluorescence intensity at day 7, significantly outperforming both free antigen and TDI^•−^ L‐gel formulations and establishing its superior antigen retention capability. Additionally, following light irradiation, the TDI^•−^ D‐gel exhibited an observable expansion in the spatial distribution of fluorescence signals, suggesting enhanced gel diffusion and penetration throughout the tumor tissue via sol‐gel transition. The sustained presence of the D‐hydrogel system enabled prolonged GSH depletion, as demonstrated by a ∼24% reduction in intratumoral GSH levels compared to the PBS group by day 7 (Figure S45). The persistent redox modulation continued to promote ICD in tumor cells, while the gel reservoir effectively retained these released antigens and synergistically amplified the antigen‐driven immune response.

### 2.6 Primary Tumor Treatment and Immune Activation

**FIGURE 5:**
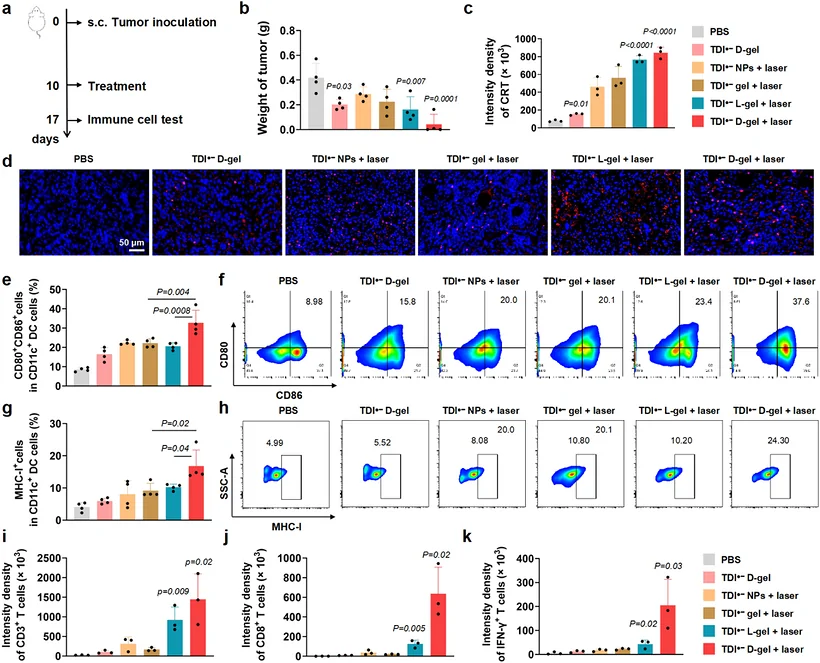
(a) Treatment timeline for in vivo anti‐tumor therapy. (b) Tumor weight analysis after different treatments. (c) Immunofluorescence images of CRT expression in tumor tissues under different treatments. (d) CRT expression levels across treatment groups. Scale bar: 50 µm. (e, f) Flow cytometry analysis of CD80^+^CD86^+^ DCs in tumor‐draining lymph nodes. (g, h) Flow cytometry analysis of MHC‐I expression on CD11c^+^ DCs. (i‐k) Immunofluorescence staining analysis of tumor‐infiltrating lymphocytes: (i) CD3^+^ T cell percentages (j) CD8^+^ cytotoxic T cell percentages (k) IFN‐γ^+^ T cell percentages. Data are presented as mean ± SD (n = 5). Statistical significance was calculated via one‐way ANOVA with Tukey's post hoc test. P < 0.05 was considered statistically significant.

The therapeutic efficacy of TDI^•−^ D‐gel was evaluated in 4T1 tumor‐bearing mice following the experimental timeline shown in Figure 5a. In addition to the distant‐tumor study, therapeutic efficacy against the primary tumor was evaluated by endpoint tumor weight and intratumoral immune analyses. Following tumor inoculation on day 0, mice were administered peritumoral injections of PBS, TDI^•−^ NPs, TDI^•−^ gel, TDI^•−^ L‐gel, or TDI^•−^ D‐gel on day 10. For the laser‐treated groups, tumors were irradiated with a 1064 nm laser (0.5 W/cm^2^, 5 min). This generated localized hyperthermia as monitored by thermal imaging (Figure S46). Among all treatment groups, the TDI^•−^ D‐gel plus laser irradiation demonstrated the most pronounced tumor inhibition, with the tumor weight being reduced by ≈90% compared to the PBS control (Figure 5b). Consistently, the tumor growth curves also showed that the TDI^•−^ D‐gel + laser group most effectively suppressed primary tumor progression during the treatment period (Figure S47). Body weight was monitored throughout the treatment period, and no obvious treatment‐related changes were observed in any group, indicating good systemic tolerability (Figure S48). In addition, blood biochemistry parameters and major organ histology remained within normal ranges (Figures S49 and S50), confirming the favorable biosafety profile of this therapeutic approach.

For a mechanistic insight into the enhanced therapeutic effects, ICD markers were examined. Immunofluorescence analysis revealed significantly elevated calreticulin (CRT) expression in the group with laser treated TDI^•−^ D‐gel. This is documented by an 11‐fold increase compared to control groups (Figure 5c,d). The immunological consequences of treatment were also analyzed. In tumor‐draining lymph nodes, the laser treated TDI^•−^ D‐gel significantly enhanced the proportion of CD80^+^CD86^+^ mature DCs compared to reference groups (Figure 5e,f). This pattern of immune activation suggests photothermal‐triggered ICD works synergistically with D‐amino acid's adjuvant properties to promote DC maturation. Consistent with sustained antigen availability, MHC‐I expression on CD11c^+^ DCs showed significant upregulation in the “TDI^•−^ D‐gel with laser” group (Figure 5g,h). This indicates enhanced antigen processing and presentation. Analysis of tumor‐infiltrating lymphocytes revealed profound immune microenvironment remodeling. The group of irradiated TDI^•−^ D‐gels significantly increased the density of CD3^+^ T cells (63‐fold), CD8^+^ cytotoxic T cells (578‐fold), and IFN‐γ^+^ T cells (39‐fold) within tumors compared to the control group (Figure 5i–k and Figure S51). This improved lymphocyte infiltration profile confirms successful conversion of immunologically “cold” tumors to “hot” phenotypes through coordinated ICD‐mediated antigen release and D‐amino acid‐enabled immune potentiation. It should be noted that the ICD readouts in Figure 5c,d were measured at an early stage after treatment, when the dominant effect is expected to arise mainly from the acute photothermal and redox insult. Therefore, the superior persistence of the D‐gel may not necessarily be reflected proportionally in these early ICD markers.

### 2.7 Systemic Antitumor Immunity and Memory Response

**FIGURE 6:**
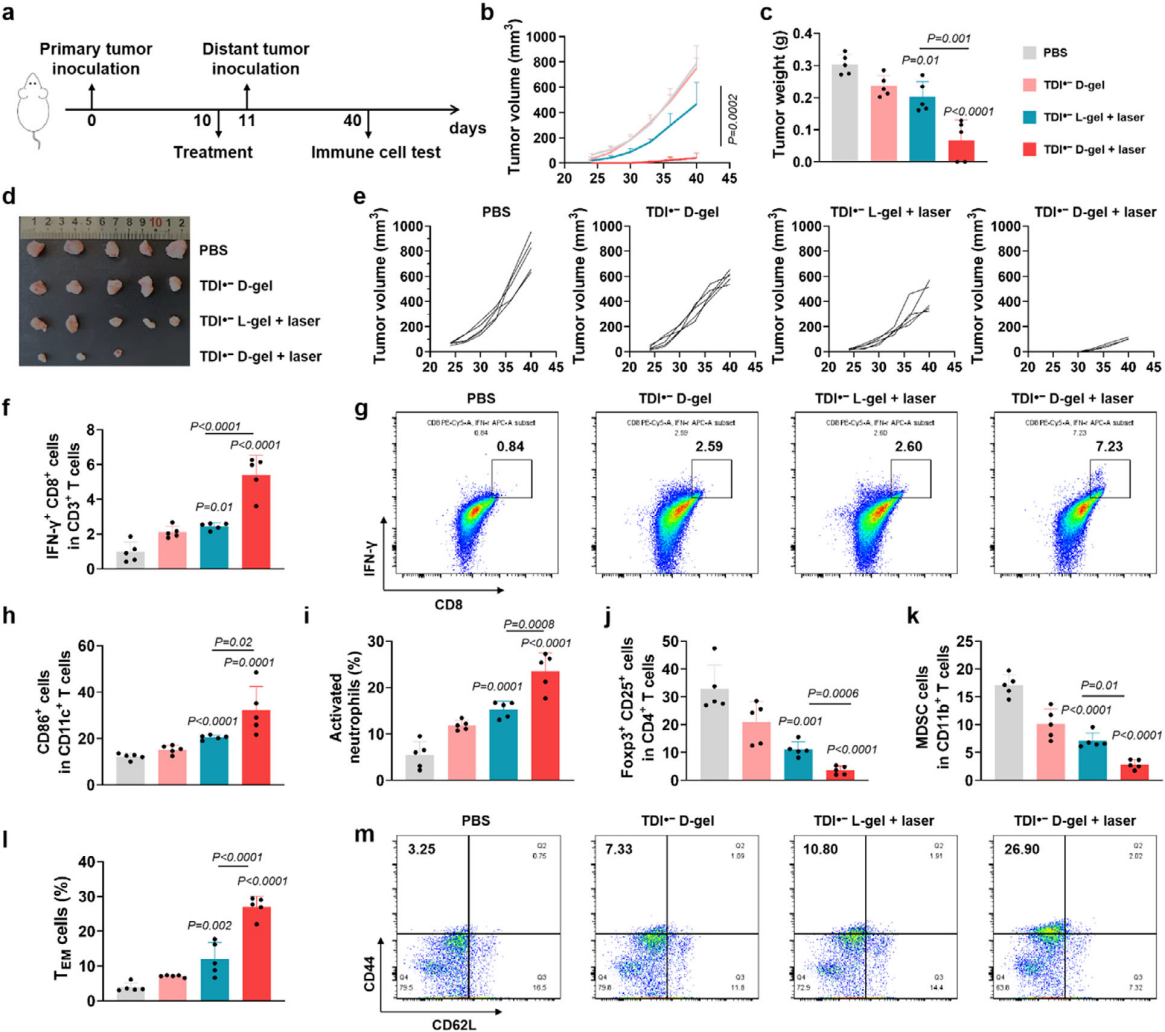
(a) Experimental timeline for the bilateral tumor model study. (b) Photographs of distant tumors of different treatment groups. (c) Growth curves of distant tumors and systemic therapeutic effects. (d) Weights of distant tumors on day 40. (e) Individual tumor growth curves of different treatment groups. (f, g) Flow cytometry analysis of IFN‐γ^+^ CD8+ T cells in distant tumors. (h) CD86^+^ CD11c^+^ dendritic cells in distant tumors detected by flow cytometry. (i) Activated neutrophil infiltration in distant tumors. (j) Regulatory T cell (Treg) populations in distant tumors. (k) Myeloid‐derived suppressor cell (MDSC) percentages. (l, m) Flow cytometry analysis of effector memory T cell (T_EM_) populations. Data are presented as mean ± SD (n = 5). Statistical significance was calculated via one‐way ANOVA with Tukey's post hoc test. P < 0.05 was considered statistically significant.

To confirm whether the robust local immune activation could translate into a systemic anti‐tumor effect, we established a bilateral 4T1 tumor model (Figure 6a). To dissect abscopal effects and immunological memory, primary tumors were inoculated on day 0, followed by therapeutic intervention (day 10) and distant tumor inoculation (day 11). Monitoring until day 40 revealed profound suppression of distal tumor progression in the “TDI^•−^ D‐gel laser group” versus all other controls (Figure 6b–d). Analysis of tumor weights revealed significantly reduced tumor burden in the treatment group, with average weights decreased by 78%, with parallelized growth curves across biological replicates confirming therapeutic consistency (Figure 6e).

Comprehensive immunophenotyping revealed successful reprogramming of the immunosuppressive microenvironment in distant tumors. Flow cytometry analysis demonstrated markedly enhanced CD8^+^ T cell responses, highlighted by a 7‐fold increase in IFN‐γ^+^ CD8^+^ T cells in the “TDI^•−^ D‐gel plus laser group” compared to the PBS group (Figure 6f,g). This was paralleled by improved antigen presentation efficiency, evidenced by increased proportions of CD86^+^ CD11c^+^ DCs from 12.2% to 32.0% (Figure 6h). There was significant infiltration of activated neutrophils, with their proportion rising from 5.5% to 23.6% (Figure 6i) and increased percentage of NK cells (Figure S52), underscoring robust innate immune activation. Furthermore, the therapeutic intervention effectively reshaped the immunosuppressive TME through multiple mechanisms. Flow cytometry analysis revealed substantial reductions of immunosuppressive cell populations, including regulatory T cells, which decreased from 32.8% to 2.0% (Figure 6j and Figure S53), and myeloid‐derived suppressor cells (MDSC), which also showed a marked reduction from 17.0% to 2.9% (Figure 6k). The persistent antitumor immunity was further supported by an increase in effector memory T cells in the treatment group, rising from 3.8% to 27.0% (Figure 6l,m). This suggests the potential of TDI^•−^ D‐gel for memory‐associated antitumor immune responses against tumor recurrence, effectively bridging localized therapy with systemic immune surveillance.

In brief, the D‐poly(amino acid) modified hydrogel orchestrates anti‐tumor immunity through carefully orchestrated mechanisms. The process is initiated by laser‐triggered hyperthermia and GSH depletion, which disrupt redox homeostasis to enhance ICD and the in situ release of tumor antigens. Then, the D‐chiral hydrogel functions as a sustained antigen reservoir, enabling prolonged DC cross‐presentation. The D‐chiral backbone simultaneously acts as an intrinsic adjuvant independent of photothermal and redox effects, amplifying T cell activation and remodeling the tumor immune microenvironment. This qualifies the TDI^•−^ D‐gel as an effective in situ vaccination platform capable of inducing a memory T cell response and offering protection against distant tumor growth.

## 3 Conclusions

In this work, we present a photothermal immunogenic amplifier based on a TDI chromophore whose redox activity is precisely controlled. This enables different redox states to amplify ICD: the stable radical anion provides the initial NIR‐II photothermal stress, while its subsequent reduction dismantles the antioxidant defenses that would otherwise suppress the immunogenic response. The facile co‐assembly of strongly electron‐withdrawing TDI chromophores with biocompatible polymers creates a nano‐confined domain that kinetically traps the radical anion in its active state. These nano‐amplifiers are then integrated within an injectable D‐chiral hydrogel, transforming the local therapeutic into an in situ vaccine platform that provides sustained release, antigen capture, and intrinsic adjuvancy. In preclinical models, light activation of this integrated system not only eradicated primary tumors but also induced a powerful systemic immune response that led to the suppression of distant tumors. Our work thus establishes a new paradigm for radical anion‐based therapeutics, moving beyond passive stabilization to the precise management of multi‐step redox activity for enhanced photothermal immunotherapy.

## 4 Experimental Section

### 4.1 Animals and Cell Lines

BALB/c female mice (5–8 weeks old) were obtained from Beijing Charles River Laboratory Animal Technology Co., Ltd. (Beijing, China). All animal experiments were conducted in accordance with the Guide for the Care and Use of Laboratory Animals and were approved by the Institutional Animal Care and Use Committee of the National Center for Nanoscience and Technology, China (approval no. NCNST21‐2407‐0602). Animal experiments and reporting were conducted with consideration of the ARRIVE recommendations. 4T1 mouse breast cancer cells (RRID: CVCL_0125) and human umbilical vein endothelial cells (HUVECs, RRID: CVCL_9Q53) were obtained from Service‐bio.

### 4.2 Synthesis and Preparation of Materials

1–4NO_2_‐TDI derivatives were synthesized from the parent TDI by controlled nitration using an optimized procedure based on a reported method [15]. NH_2_‐F127, F127‐D/L‐P_Glu_, and F127‐D/L‐P_Lys_ were synthesized according to modified literature procedures. TDI^•−^ NPs were prepared by co‐assembly of 4NO_2_‐TDI with Pluronic F127. TDI^•−^ D/L‐gels were prepared by incorporating TDI^•−^ NPs into supramolecular hydrogels formed from F127‐grafted chiral poly(amino acids) and α‐cyclodextrin.

### 4.3 Biological and In Vivo Evaluations

Protein‐ and antigen‐retention assays were performed using FITC‐BSA and 4T1‐cell‐derived lysates as model substrates. Intracellular GSH, ROS, ICD, cytotoxicity, and BMDC activation studies were performed using standard procedures In vivo retention, primary tumor treatment, and bilateral tumor experiments were carried out in murine 4T1 tumor models using standard procedures.

### 4.4 Statistical Analysis

All quantitative data were presented as mean ± SD unless otherwise stated. The value of n was defined in the corresponding figure legends and represents biologically independent samples, animals, or independently prepared material samples, as appropriate. No data normalization or transformation was applied unless explicitly stated for a specific assay. No data points were excluded unless justified by clear experimental failure; if applicable, such exclusion criteria were predefined. Statistical analyses were performed using GraphPad Prism 8.0. For comparisons between two groups, two‐tailed unpaired Student's t‐tests were used. For comparisons among multiple groups, one‐way or two‐way ANOVA followed by Tukey's post hoc multiple‐comparison test was used, as specified in the figure legends. Survival data, where applicable, would be analyzed using the Kaplan–Meier method with a log‐rank test. Differences were considered statistically significant at *P* < 0.05.

## AI Use Statement

No AI or machine‐generated tools were used in the preparation of this manuscript, including writing, figure preparation, data analysis, or interpretation of results. All scientific content, data interpretation and figure preparation were generated and verified solely by the authors.

## Conflicts of Interest

The authors declare no conflicts of interest.

## Supporting information




**Supporting File**: advs76277‐sup‐0001‐SuppMat.docx.

## Data Availability

The data that supports the findings of this study are available in the supplementary material of this article.
