# Supplementary material for: Nano‐Confined Radical Anion as An NIR‐II Photothermal Immunogenic Amplifier for In Situ Cancer Vaccination
Source: Adv Sci (Weinh). 2026 Jun 26:e76277. Online ahead of print. doi: 10.1002/advs.76277 (PMC13336734; doi:10.1002/advs.76277)
Supplement: Supplementary file 1 — Supporting File: advs76277‐sup‐0001‐SuppMat.docx. [file ADVS-9999-e76277-s001.docx]

Supporting Information

Nano-Confined Radical Anion as an NIR-II Photothermal Immunogenic Amplifier for in Situ Cancer Vaccination

*Mengxin Mu^‡[a]^, Mengyu Guo^‡[b]^, Jun Guan^[a]^, Xiaofeng He^[a]^, Mingdi Hu^[b]^, Fene Gao^[b]^, Klaus Müllen^[c]^, Chendong Ji*^[a]^, Chunying Chen*^[b]^, Meizhen Yin*^[a]^*

[a] Mengxin Mu, Dr. Jun Guan, Xiaofeng He, Dr. Chendong Ji, Prof. Meizhen Yin

State Key Laboratory of Chemical Resource Engineering, Beijing University of Chemical Technology, Beijing 100029, China

E-mail: jicd@mail.buct.edu.cn (ORCID: 0000-0003-4913-1645);

yinmz@mail.buct.edu.cn (ORCID: 0000-0001-8519-8578)

[b] Dr. Mengyu Guo, Mingdi Hu, Fene Gao, Prof. Chunying Chen

CAS Key Laboratory for Biomedical Effects of Nanomaterials and Nanosafety & CAS Center for Excellence in Nanoscience, National Center for Nanoscience and Technology of China, Beijing 100190, China

E-mail: chenchy@nanoctr.cn (ORCID: 0000-0002-6027-0315)

[c] Prof. Klaus Müllen

Max Planck Institute for Polymer Research, Ackermannweg 10, D-55128 Mainz, Germany

E-mail: muellen@mpip-mainz.mpg.de

**Experimental Section/Methods**

**Animal housing and cell culture**

The mice were housed on a 12 h light-dark cycle at 20-26 °C with 30-70% humidity. The 4T1 cells and the HUVEC cells were cultured in Dulbecco’s Modified Eagle’s Medium (DMEM) supplemented with 10% Fetal Bovine Serum (FBS) and 1% antibiotics (penicillin-streptomycin). All cells were maintained at 37 °C and 5% CO_2_.

**Materials**

HNO_3_ (68%), F127, α-CD and MCB fluorescence probe were all obtained from HEOWNS and used without further purification. D/L-Glu(Obzl)-NCA were purchased from Enlai Bio-tech. TDI were synthesized according to literature procedures. Cell Counting Kit (CCK-8) reagent, FBS, DMEM, Calcein-AM, Propidium Iodide (PI) were purchased from Solarbio. The ATP assay kit and FITC-BSA were purchased from Beyotime (Shanghai, China). Lumit™ HMGB1 (Human/Mouse) immunoassay was purchased from Promega (Beijing, China) Biotech Co., Ltd. MO IL-12 uncoated ELISA Kit and Mouse TNF-α uncoated ELISA Kit were from Invitrogen (San Diego, CA, USA). APC-Cyanine7 Anti-Mouse CD11c (N418), PE Anti-Mouse CD80 (16-10A1), APC Anti-Mouse CD86 (GL-1), FITC Anti-Mouse MHC Class II (M5/114.15.2), PE-Cyanine7 Anti-Mouse CD62L (L-Selectin) (MEL-14) and APC Anti-Human/Mouse CD44 (IM7) were obtained from Tonbo Biosciences (USA).

**Measurements**

Nuclear magnetic resonance (NMR) spectra were recorded on a Bruker 400 (400 MHz ^1^H) spectrometer and a Bruker 600 (600 MHz ^13^C) spectrometer, using CDCl_3_ and DMSO-d*_6_* as solvents and tetramethylsilane (TMS) as internal reference respectively. Mass spectra (MS) were measured with a mass spectrometer (Waters, Xevo G2 Qtof, USA). Matrix-assisted laser-desorption ionization time-of-flight mass spectrometry (MALDI-TOF MS) were determined on AXIMA-CFR plus MALDI-TOF mass spectrometer The UV-Vis spectra were determined by a spectrophotometer (UV-2600, Shimadzu, Japan) in a quartz cuvette. An 1064 nm laser (Stone-laser LTD, Beijing, China) was used for irradiation. The temperatures of samples were recorded by an IR-thermal camera (Ti400, Fluke, USA). Morphologies of nanoparticles and hydrogel were observed with SEM (Hitachi S-4700) and TEM (JEM-3010, JEOL, Japan). Crystalline phases of the materials were determined using an X-ray diffractometer (Ultima IV). Sizes and Zeta potential were measured with a Zetasizer Nano ZS (Brookhaven, Omni, USA). EPR measurements were performed at X-band (~9.5 GHz) using a Bruker Elexsys E680-X/W EPR spectrometer. CV measurements were performed using a CH Instruments potentiostat in a conventional three-electrode configuration. The electrochemical cell consisted of a platinum disk working electrode, a saturated calomel electrode (SCE) as the reference electrode, and a platinum wire auxiliary electrode.

**Synthesis methods**

*Synthesis of 1-4NO_2_-TDI*

TDI was synthesized and purified as previously reported^7^. In a dry and degassed 100 mL round bottom flask, TDI (100 mg, 0.1198 mmol) was dissolved in 50 mL of dry CHCl_3_. After 15 minutes, nitric acid (265 μL, 5.99 mmol) was added. The reaction time was controlled at room temperature to obtain TDI with different degrees of nitration. Specifically, 1NO_2_-TDI, 2NO_2_-TDI, 3NO_2_-TDI, and 4NO_2_-TDI were obtained by reacting the samples for 2 h, 12 h, 24 h, and 72 h, respectively. The completion of the reaction was monitored by TLC, and the remaining acid was quenched by a saturated solution of KOH. The organic layer was collected using chloroform, dried over anhydrous Na_2_SO_4_, and concentrated under reduced pressure. The crude mixture was subjected to silica gel column chromatography using DCM and hexane as eluents. 1NO_2_-TDI: ^1^H NMR (400 MHz, CDCl_3_) δ 8.93 - 8.79 (m, 3H), 8.83 (s, 2H), 8.79 - 8.65 (m, 3H), 8.35 (d, J = 8.4 Hz, 1H), 7.59 - 7.49 (m, 1H), 7.39 (dd, J = 7.8, 2.4 Hz, 3H), 5.41 - 5.30 (m, 1H), 2.85 - 2.70 (m, 3H), 2.25 (t, J = 7.6 Hz, 1H), 2.04 (d, J = 6.3 Hz, 1H), 1.66 (s, 1H), 1.28 (s, 7H), 1.22 (d, J = 6.8 Hz, 18H), 0.94 - 0.87 (m, 1H), 0.09 (s, 1H). m/z [M]+ calcd for [C_58_H_45_N_3_O_6_]+: 880.3308, found: 880.3451. 2NO_2_-TDI: ^1^H NMR (400 MHz, CDCl_3_) δ 1.28 (s, 4H), 1.28 (s, 2H), 1.22 (dd, J = 6.8, 2.0 Hz, 1H), 0.95 - 0.83 (m, 1H), 0.87 (s, 2H), 0.10 (s, 1H), 0.10 (s, 13H). m/z [M]+ calcd for [C_58_H_44_N_4_O_8_]+: 925.3159, found: 925.3242. 3NO_2_-TDI: ^1^H NMR (400 MHz, CDCl_3_) δ 9.01 - 8.91 (m, 1H), 8.89 - 8.81 (m, 1H), 8.33 (d, J = 16.6 Hz, 1H), 7.56 (td, J = 8.0, 5.2 Hz, 1H), 7.40 (dt, J = 7.8, 2.0 Hz, 2H), 2.73 (dq, J = 23.6, 6.8 Hz, 1H), 2.04 (d, J = 6.4 Hz, 1H), 1.29 (s, 7H), 1.28 (s, 2H), 1.22 (d, J = 6.8 Hz, 11H), 0.99 - 0.87 (m, 1H), 0.09 (s, 1H). m/z [M]+ calcd for [C_58_H_43_N_5_O_10_]+: 970.3010, found: 970.3073. 4NO_2_-TDI: ^1^H NMR (400 MHz, CDCl_3_) δ 8.98 (s, 4H), 8.34 (s, 4H), 7.57 (t, J = 7.8 Hz, 2H), 7.41 (d, J = 7.8 Hz, 4H), 2.69 (h, J = 6.9 Hz, 4H), 1.22 (d, J = 6.8 Hz, 25H). m/z = calcd for C_58_H_42_N_6_O_12_: 1014.2861, found: 1014.2740.

*Synthesis of NH_2_-F127*

NH_2_-F127 was synthesized according to the reported method^44^. 25 g dried F127 was dissolved in 100 mL anhydrous CH_2_Cl_2_, with 50 mL anhydrous pyridine and 0.60 g 4-methylbenzenesulfonyl chloride added. The mixture was allowed to react at room temperature for 24 h. The product was extracted with 3 M HCl, and the organic phase was washed with 5.0 g of NaHCO_3_. The product was recrystallized from THF/diethyl ether and dried under vacuum to give a 92% yield of F127-sulfanilic acid ester. The ester was dissolved in 250 mL of NH_3_ and reacted at 80 °C for 16 h. The product was extracted with CH_2_Cl_2_, and the organic phase was mixed with 1 M NaOH for 2 h. The organic phase was washed to neutral, and the solvent was evaporated to give a 72% yield of NH₂-F127.

*Synthesis of D/L-Lys(Obzl)-NCA*

D-Lys(Obzl)-NCA was synthesized according to the reported method. Briefly, 1.0 g D-Lys(Obzl) (3.58 mmol) was dispersed in 25 mL anhydrous THF. Then, 0.425 g triphosgene (1.425 mmol) was dissolved in 10 mL THF and added slowly into the D-Lys(Obzl) suspension. The reaction was carried out at 40 °C under a dry argon atmosphere for about 1 h. After the mixture became transparent, THF was evaporated under reduced pressure. The obtained solid was re-crystallized three times from a mixture of THF/petroleum ether and then dried at room temperature in a vacuum. The yield was 80.5% (1.14 g). L-Lys(Obzl)-NCA was prepared using the same method.

*Synthesis of F127-D/L-PGlu and F127-D/L-PLys*

D-PGlu-F127 was synthesized by the ring-opening polymerization of D-Glu(Obzl)-NCA initiated by the free amino groups of NH_2_-F127. Specifically, 1.0 g (3.26 mmol) D-Glu(Obzl)-NCA was dissolved in 8 mL DMF. Then 1.95 g (0.39 mmol) NH_2_-F127 was dissolved in 7 mL THF and added to the D-Glu(Obzl)-NCA solution. The reaction mixture was stirred for 72 h at 40 °C under a dry argon atmosphere. The reaction mixture was poured into diethyl ether to precipitate the product, which was then collected by filtration and dried under vacuum, yielding the protected polymer in 65% yield. The obtained D-PGlu(Obzl)-F127 was then deprotected using HBr. 0.70 g of the protected polymer was dissolved in 5 mL acetic acid and stirred for 1 h, followed by addition of 15 mL of 40% HBr solution in acetic acid. After vigorous stirring for 30 min in an ice bath, a precipitation was observed. The precipitate was collected, dissolved in 5 mL distilled water, and dialyzed for 3 days to remove any unreacted NH_2_-F127 and other reactants. The final product, F127-D-PGlu, was obtained after lyophilization. F127-L-PGlu and F127-D/L-PLys were obtained using the same synthetic approach.

**Preparation and physicochemical characterization of TDI^•-^ NPs**

1-4NO_2_-TDI and F127 were dissolved in THF, 2 mL, followed by dropwise addition into ultrapure water (6 mL) under sonication in a water bath for 10 minutes. The THF was subsequently removed via rotary evaporation to obtain the TDI^•-^ NPs solution (50 μM). The same procedure was applied to synthesize 1NO_2_-TDI@F127, 2NO_2_-TDI@F127, and 3NO_2_-TDI@F127 NPs.

The stability of TDI^•-^ NPs were systematically investigated under various conditions. For long-term stability assessment, the TDI^•-^ NPs were dispersed in PBS buffer to achieve a final TDI^•-^ concentration of 50 μM. The particle size distribution was monitored in triplicate using DLS measurements and UV-Vis spectroscopy over an 8-week period at room temperature. Additionally, the stability of TDI^•-^ NPs was evaluated under different experimental conditions, including various temperature conditions, different light exposure conditions and different atmospheric environments.

**Antigen capture study and preparation of antigen-captured TDI^•-^ NPs**

*Collection of TAAs*

First, 4T1 cells cultured in culture dishes were washed with PBS, and the complete medium was replaced with serum-free medium. The culture dish containing cells was then placed in a water bath at 50 °C for 5 minutes, followed by incubation at 37 °C for 48 hours. Subsequently, insoluble cellular debris was removed by centrifugation at 5000 rpm for 5 minutes, and the supernatant containing TAAs was collected.

The protein adsorption capacity of the *in situ*-forming hydrogel was determined using a BCA assay kit, thereby quantifying the amount of protein captured by the forming hydrogel.

*Preparation of antigen-captured TDI^•-^ gel*

For the BMDC activation assay, antigen-loaded hydrogels were prepared by incubating 100 μL hydrogel with 100 μL antigen-containing supernatant at 37°C in a shaking incubator for 12 h. The amount of antigen adsorbed/loaded in the hydrogel under these conditions was determined to be 0.03 mg/mL. To ensure a valid comparison, the antigen alone control was normalized to the same antigen amount as that contained in the antigen-loaded hydrogel formulation. Unless otherwise specified, each material sample was prepared independently for each well.

**Preparation and characterization of the TDI^•-^ D/L-gel**

TDI^•-^ NPs (10 mg), F127-D-PGlu (10 mg) and F127-D-PLys (10 mg) were dissolved in 1 mL of PBS solution (10 mM, pH 7.4). Under sonication, α-CD (100 mg/mL) in PBS solutions were slowly added dropwise. After 30 minutes of sonication, the mixed solution was allowed to stand, and the gelation time was determined using the test tube inverting experiment. The microstructures of the hydrogels were observed through SEM and TEM. The interactions between F127 and α-CD in the hydrogels were measured using an X-ray diffractometer. The sol-gel transition temperatures of TDI^•-^ gels of different ratios of α-CD and F127 were determined using an inverted-vial method.

**In vitro protein adsorption study of TDI^•-^ D-gel**

50 μL of hydrogel dispersions containing different concentrations of D-Glu or D-Lys were added to each well of a 96-well plate. The samples were kept at room temperature for 1 h to form hydrogels, followed by the addition of 50 μL fetal bovine serum (FBS). After co-incubation with FITC-BSA in a shaking incubator (37 °C, 100 rpm) for 24 h, the protein content in the supernatant was determined using a fluorescence spectrophotometer protein quantification kit. The Zeta potential of hydrogels after protein adsorption was also measured to evaluate the surface charge properties.

**Synthesis and characterization of Cy7-BSA conjugates**

A modified conjugation protocol was developed for the preparation of Cy7-labeled BSA. Specifically, Cy7 (0.8 mg) and BSA (10 mg) were individually dissolved in anhydrous dimethyl sulfoxide (DMSO, 1 mL). The solutions were subsequently combined and homogenized via gentle pipetting to initiate the coupling reaction. The reaction mixture was then incubated overnight at ambient temperature under dark conditions to ensure complete conjugation. The crude product was purified via dialysis using a membrane with a molecular weight cut-off (MWCO) of 10 kDa. The dialysis process was performed with three buffer exchanges at 4-hour intervals to effectively remove unreacted components. The purified Cy7-BSA conjugates were collected and stored at -20 °C until further use.

**Photothermal properties and caclculation of the photothermal conversion efficiency**

The TDI^•-^ NPs aqueous solution (2 mL, 50 μM) or TDI^•-^ gel (2 mL, 1-10 mg/mL) was irradiated with an 1064 nm laser (0.25-1 W/cm^2^). The TDI^2-^ NPs aqueous solution (2 mL, 50 μM) was also irradiated with 660 nm lasers (0.5W/cm^2^). The temperatures were monitored by a Fluke (Ti400) thermal imaging camera. The temperature was recorded until it reached a stable maximum, and then the laser was turned off to record the cooling curve. In addition, the effect of different laser power densities (0.25, 0.5, 0.75, and 1 W/cm^2^) of TDI^•-^ NPs temperature changes was measured as described above. The photothermal conversion efficiency (η) was calculated using equation (1):

**η = [hA(T_max_-T_amb_) - Q_dis_] / I(1-10^-A1064^) (1)**

where h and A were the heat transfer coefficient and surface area of the container, respectively. T_max_ was the maximum steady-state temperature, T_amb_ was the ambient temperature. Q_dis_ was the heat dissipation of the solvent (water), which can be derived from equation (2):

**Q_dis_ = h_A_ (T_max_ (water) - T_amb_ (water)) (2)**

“I” was incident laser power (1.0 W/cm^2^), and A_0_ was absorbance at 1064 nm. h_A_ was calculated using the following equation (3):

**h_A_ = Σm_i_ C_i_ / τs (3)**

where m and C were the mass (2 g) and heat capacity (4.2 J/g) of water, respectively. τs was the sample system time constant, which can be calculated using equation (4):

**τs = -T / lnθ (4)**

where θ represented the dimensionless driving force defined as (T - T_amb_) / (T_max_ - T_amb_), and T was time.

**Cytotoxicity experiments**

CCK-8 assays and Live-cell imaging were performed to assess cell viability under different treatments. 4T1 cells or HUVEC cells were seeded in 96-well plates at a density of 5000 cells per well and cultured for 24 h at 37 °C in a 5% CO_2_ atmosphere. The cells were then incubated with TDI^•-^ D-gel at concentrations ranging from 0 to 60 mg/mL, with or without 1064 nm laser irradiation (0.5 W/cm^2^, 5 min). After 24 hours:

For Live-cell imaging: Cells were incubated with PBS buffer (100 μL per well) containing Calcein-AM (2 μM) and PI (5 μM) for 20 min in the cell culture chamber. The cells were then washed three times with PBS and observed under a fluorescence microscope.

For CCK-8 assay: The culture medium was replaced with fresh medium containing 10% CCK-8 reagent, and the cells were incubated for an additional 1 h. The optical density at 450 nm was measured using a microplate reader. All experiments were performed in triplicate and the results were presented as mean ± SD relative to the untreated control group.

The gel concentration range used in the cytotoxicity and related cellular assays was selected based on preliminary evaluation of formulation stability and handling in the in vitro setting, together with the concentration range that showed measurable activity in the protein-retention experiments.

**Cellular ROS generation assay**

The intracellular reactive oxygen species (ROS) generation was measured using the 2',7'-dichlorofluorescin diacetate (DCFH-DA) assay. 4T1 cells were seeded in 96-well plates at a density of 5000 cells per well and incubated for 24 h. The cells were then incubated with PBS, TDI^•-^ NPs or TDI^•-^ gel for 4 h. After the incubation, the cell culture medium was replaced with fresh DMEM containing DCFH-DA solution at a concentration of 1 × 10^-6^ M, and the cells were incubated for 20 min. The cells were then washed twice with PBS and observed under a fluorescence microscope to evaluate the ROS generation. The excitation wavelength and emission wavelength were 485 nm and 525 nm, respectively.

**Cellular GSH depletion assay**

4T1 cells were seeded in 96-well plates and incubated with PBS, TDI^•-^ NPs or TDI^•-^ gel. At different time points, the cell culture medium was replaced with medium containing the monochlorobimane probe. After 20 min of incubation, the cells were washed twice with PBS and imaged under a fluorescence microscope. The monochlorobimane fluorescence signals were observed in the emission wavelength range of 420-485 nm.

**Detection of *in vitro* immunogenic cell death**

4T1 cells were treated with PBS, TDI^•-^ D-NPs diluent or TDI^•-^ D-gel diluent at a gel concentration of 50 mg/mL for 4 hours. The cells were then irradiated with or without 660 nm or 1064 nm laser at a power density of 0.5 W/cm^2^ for 5 minutes. After 4 hours incubation, the extracellular release of ATP and HMGB1 was examined using the ATP assay kit and Lumit™ HMGB1 (Human/Mouse) immunoassay according to the manufacturer's instructions, respectively.

***In vitro* BMDCs activation**

Murine BMDCs were isolated from femurs and tibiae of BALB/c female mice, and were cultured in in RPMI1640 medium supplemented with 10% fetal bovine serum (FBS), 1% penicillin and streptomycin (PS, 1×), 2 mM L-glutamine, 50 μM β-mercaptoethanol, and 25 mM HEPES buffer. BMDCs were induced to differentiation using the medium mentioned above plus 20 ng/mL interleukin-4 (IL-4) and 20 ng/mL granulocyte-macrophage colony-stimulating factor (GM-CSF). Next, BMDCs were seeded into 6-well plates (1×10^6^ cells/well) and cultured in the medium without cytokines. After treatment with PBS, antigen, TDI^•-^ gel + antigen, TDI^•-^ L-gel + antigen and TDI^•-^ D-gel + antigen, the BMDCs were harvested and stained with antibodies against CD11c, CD80, CD86, MHC class I, and MHC class II at 37 °C for 30 min and then analyzed by flow cytometry. For cytokine production analysis, the supernatant was collected and analyzed for TNF-α and IL-12 using an ELISA kit according to the manufacturer’s instructions.

**Tumor distribution quantification**

To examine the accumulation and localization of different gels in the tumors, mice were divided into five groups (antigen, TDI^•-^ gel + antigen, TDI^•-^ L-gel + antigen, TDI^•-^ D-gel + antigen and TDI^•-^ D-gel + antigen + laser [1064 nm laser at 0.5 W/cm^2^ for 5 min]). The mice were imaged at predetermined time points after peritumoral injection (fluorescence excitation 650 nm, emission 670 nm, autoexposure setting, n = 3 mice in each group) using an IVIS Spectrum Imaging System (PerkinElmer). Regions of interest (ROIs) were designated, and the average radiant efficiency [p/s/cm^2^/sr]/[μW/cm^2^] was measured in each ROI using Living Image software.

**Study of *in vivo* tumor immunotherapeutic**

BALB/c female mice (6-week-old) were inoculated subcutaneously with 1×10^5^ 4T1 cells into the right side of the back on day 0. Tumors were allowed to grow for ten days, and then these tumor-bearing mice were randomly divided into six groups (n=4 for each group). On day 10, mice were treated with 80 μL PBS, TDI^•-^ D-gel, TDI^•-^ NPs+ laser, TDI^•-^ gel + laser, TDI^•-^ L-gel + laser and TDI^•-^ D-gel + laser, respectively. Tumors were collected on day 17 for weight measurement and histological analysis. Major organs underwent H&E staining for toxicity assessment, while draining lymph nodes were processed for flow cytometric analysis of dendritic cell activation markers (CD11c, CD80, CD86, MHC I).

**Inhibition of distant tumor growth**

As described above, BALB/c female mice were inoculated subcutaneously with 1×10^5^ 4T1 cells into the right side of the back. Then on day10, mice were treated with PBS, TDI^•-^ D-gel, TDI^•-^ L-gel + laser and TDI^•-^ D-gel + laser. 11 days after inoculation of the primary tumor, 5 × 10^4^ 4T1 cells were injected into the left side of each mouse to establish distant tumors. The distant tumor volumes were recorded until the end of the experiment. The larger (L) and smaller (S) diameters of tumors were monitored every other day using calipers in three dimensions, and their volumes were calculated using the formula V=0.5×L×S^2^. To evaluate immune responses, distant tumors and spleens were harvested for flow cytometry analysis. Tumor-infiltrating immune cells were analyzed for CD8^+^ T cells (CD3^+^CD8^+^), IFN-γ^+^CD8^+^T cells, DCs (CD11c^+^CD86^+^), neutrophils (CD11b⁺Ly6G⁺), Tregs (CD3^+^CD4^+^CD25^+^Foxp3^+^), and MDSCs (CD11b^+^Gr-1^+^). Splenic effector memory T cells (T_EM_, CD44^+^CD62L^-^) were also assessed.

**Supplementary Figures**


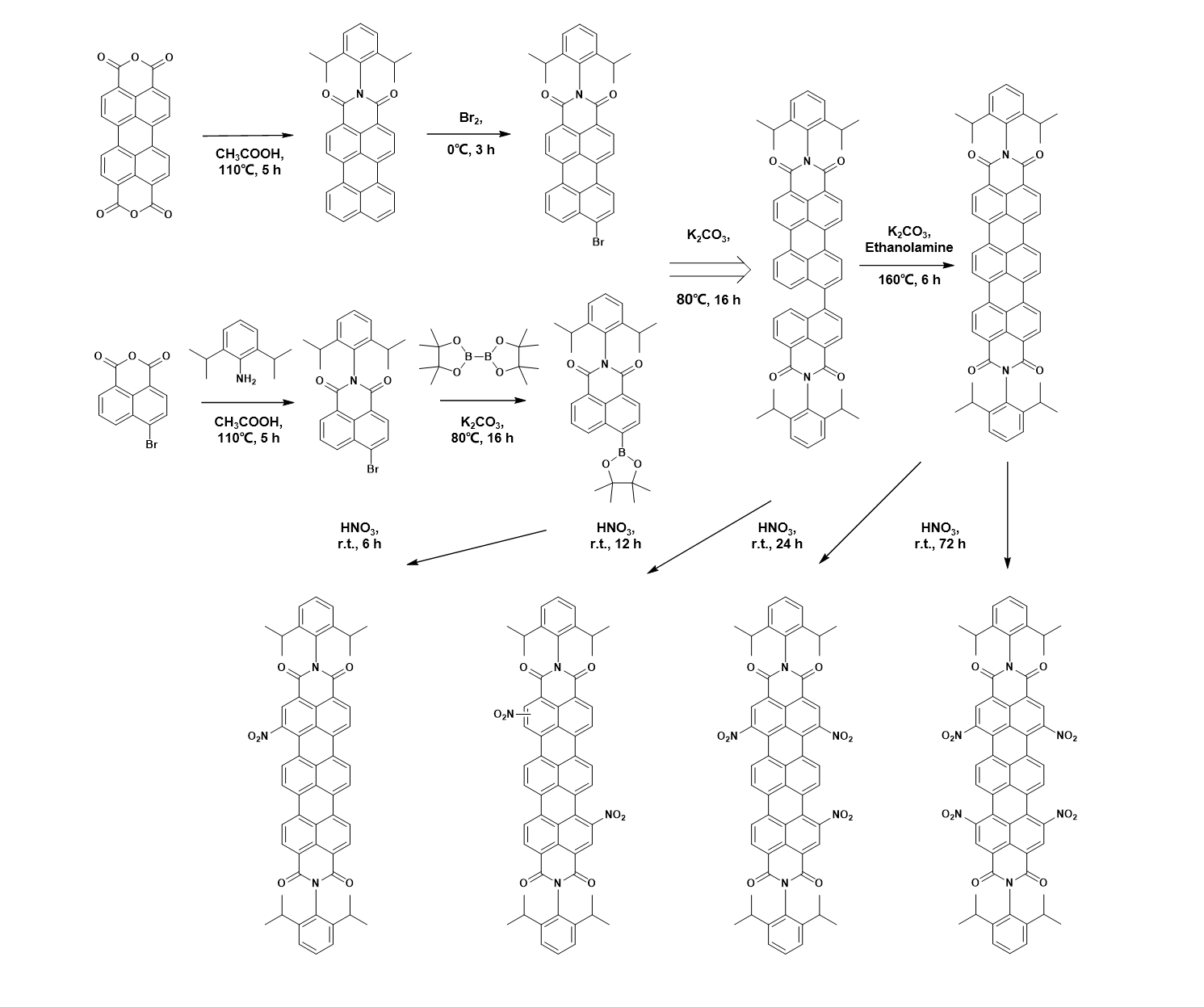


**Scheme S1.** Synthetic routes toward 1NO_2_-TDI, 2NO_2_-TDI, 3NO_2_-TDI and 4NO_2_-TDI.


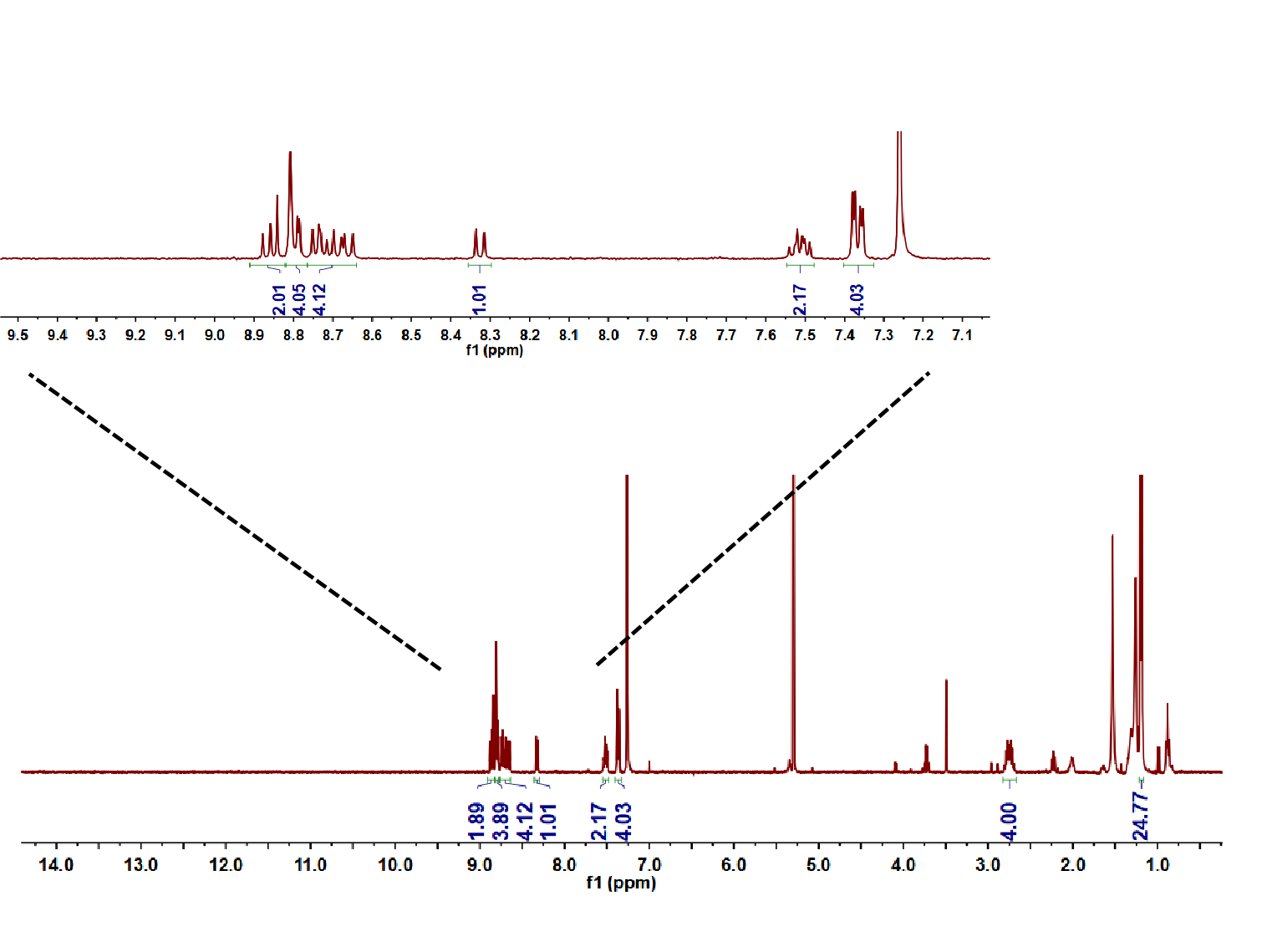


**Figure S1.** ^1^H NMR spectrum of 1NO_2_-TDI (400 MHz, CDCl_3_, 298 K).


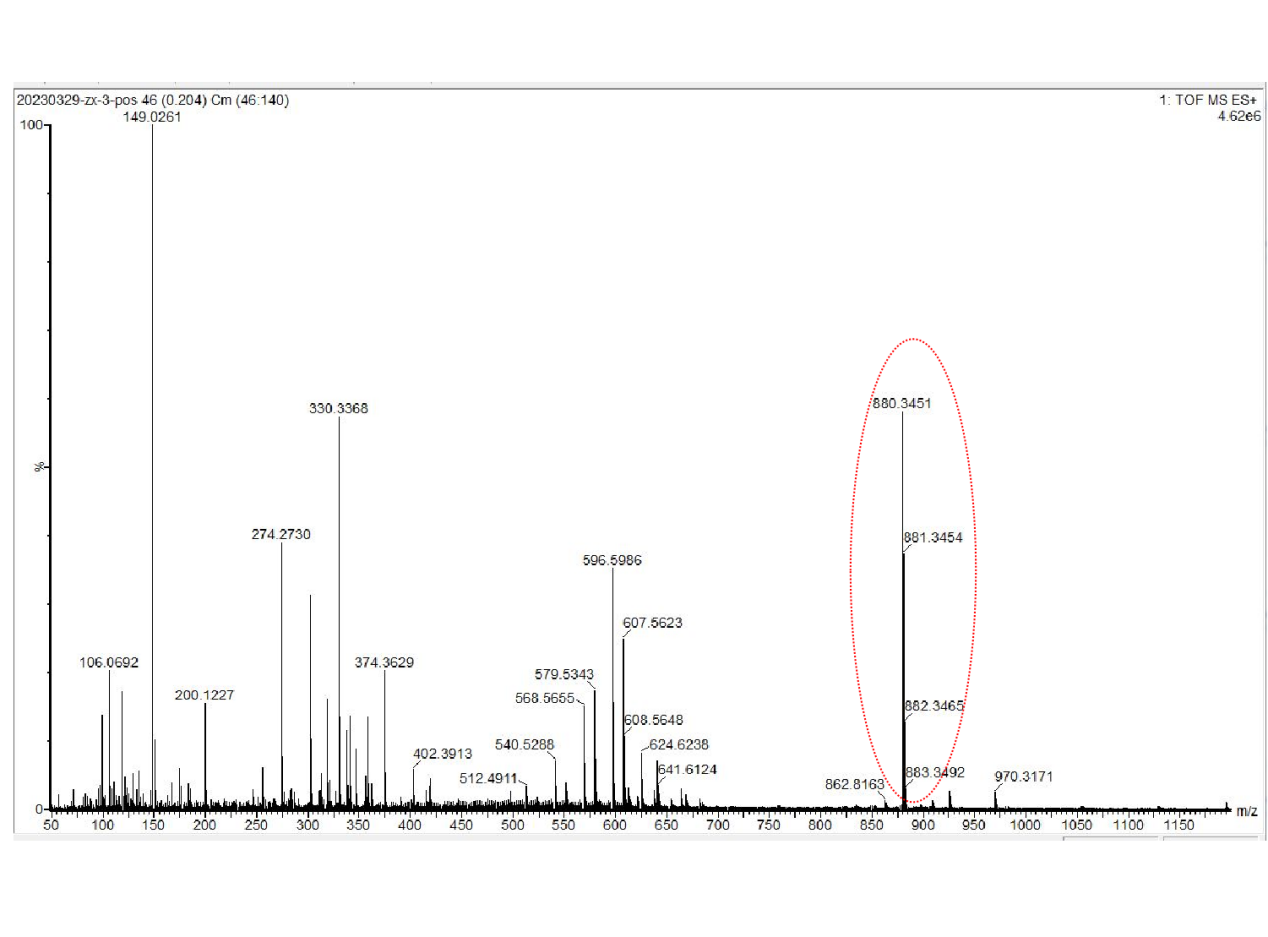


**Figure S2.** Mass spectrum of 1NO_2_-TDI (Exact mass = 879.3308).


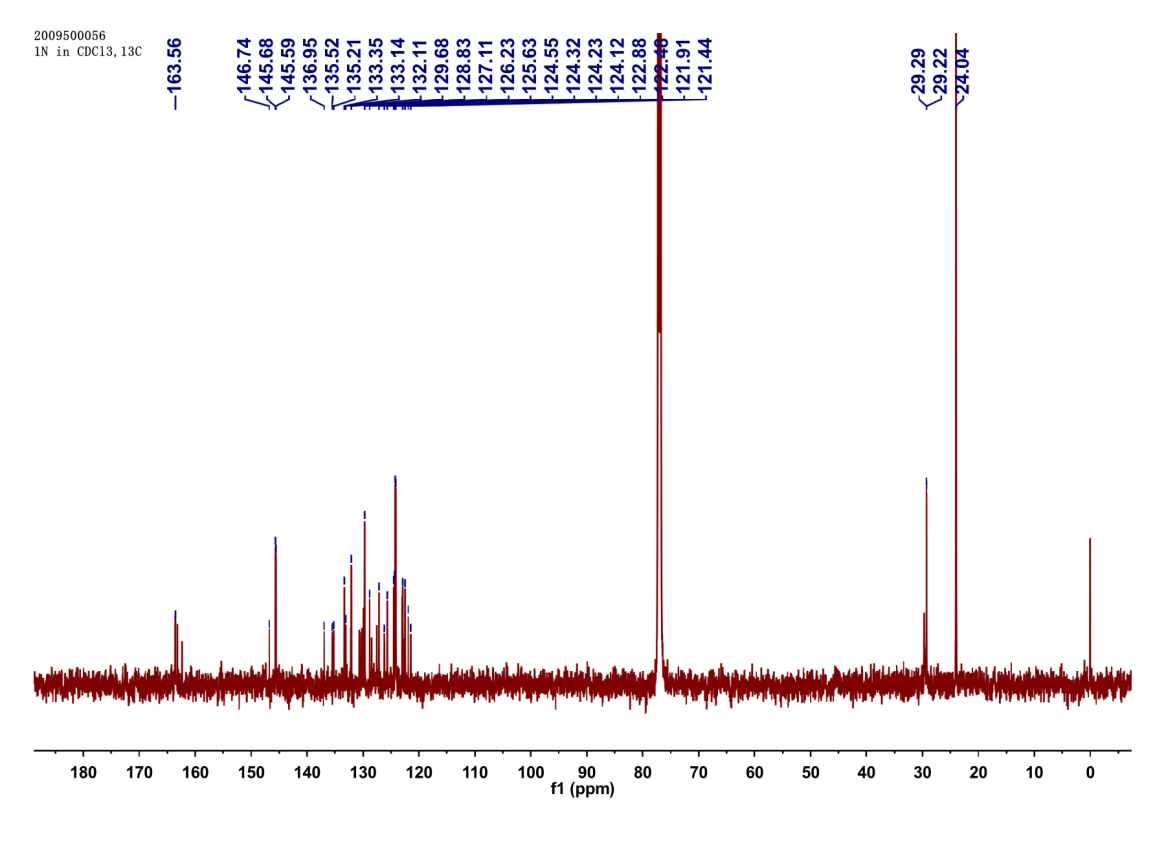


**Figure S3.** ^13^C NMR spectrum of 1NO_2_-TDI (600 MHz, CDCl_3_, 298 K).


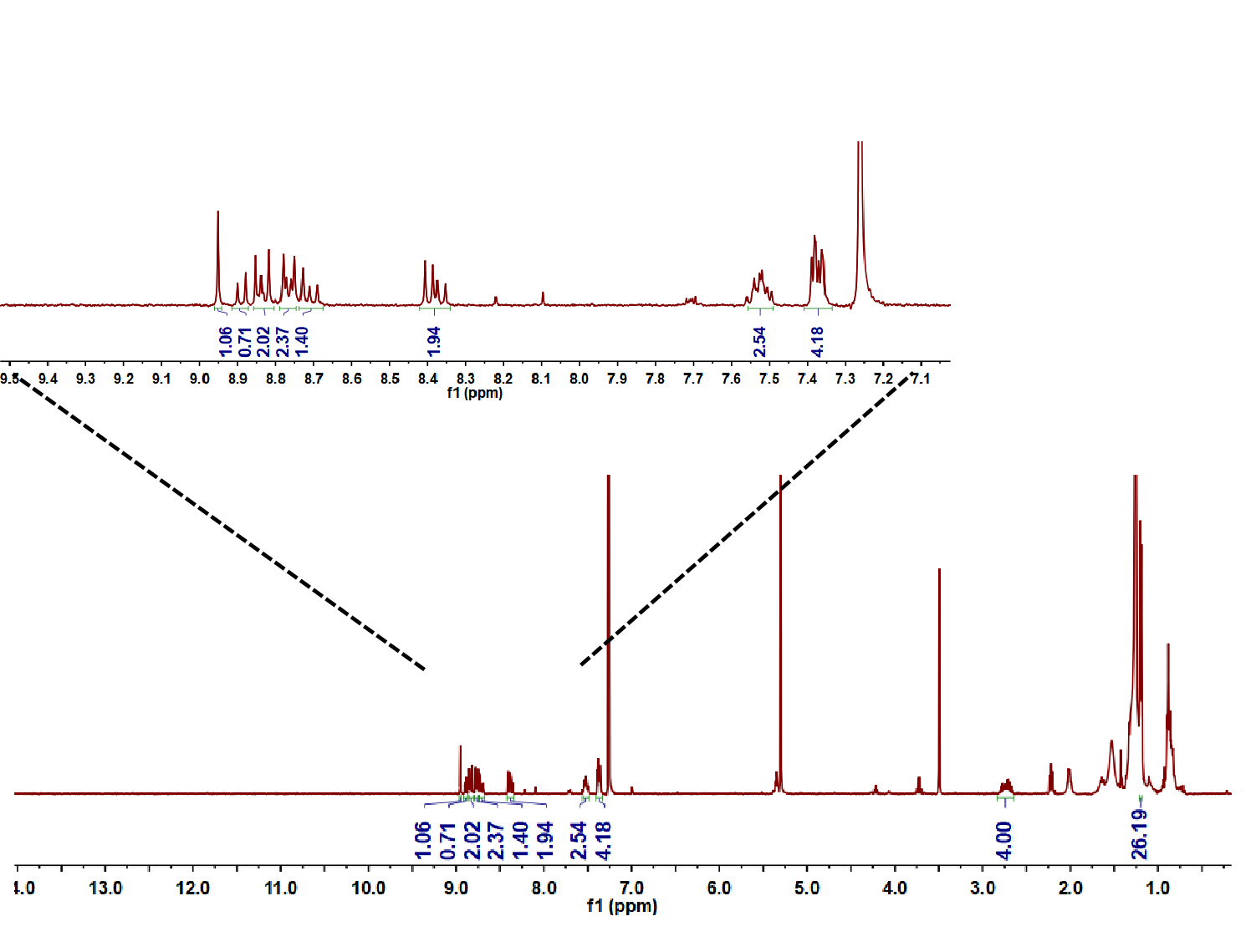


**Figure S4.** ^1^H NMR spectrum of 2NO_2_-TDI (400 MHz, CDCl_3_, 298 K). The compound exists as a mixture of positional isomers with similar physicochemical and electrochemical properties and was used without further purification^44^.


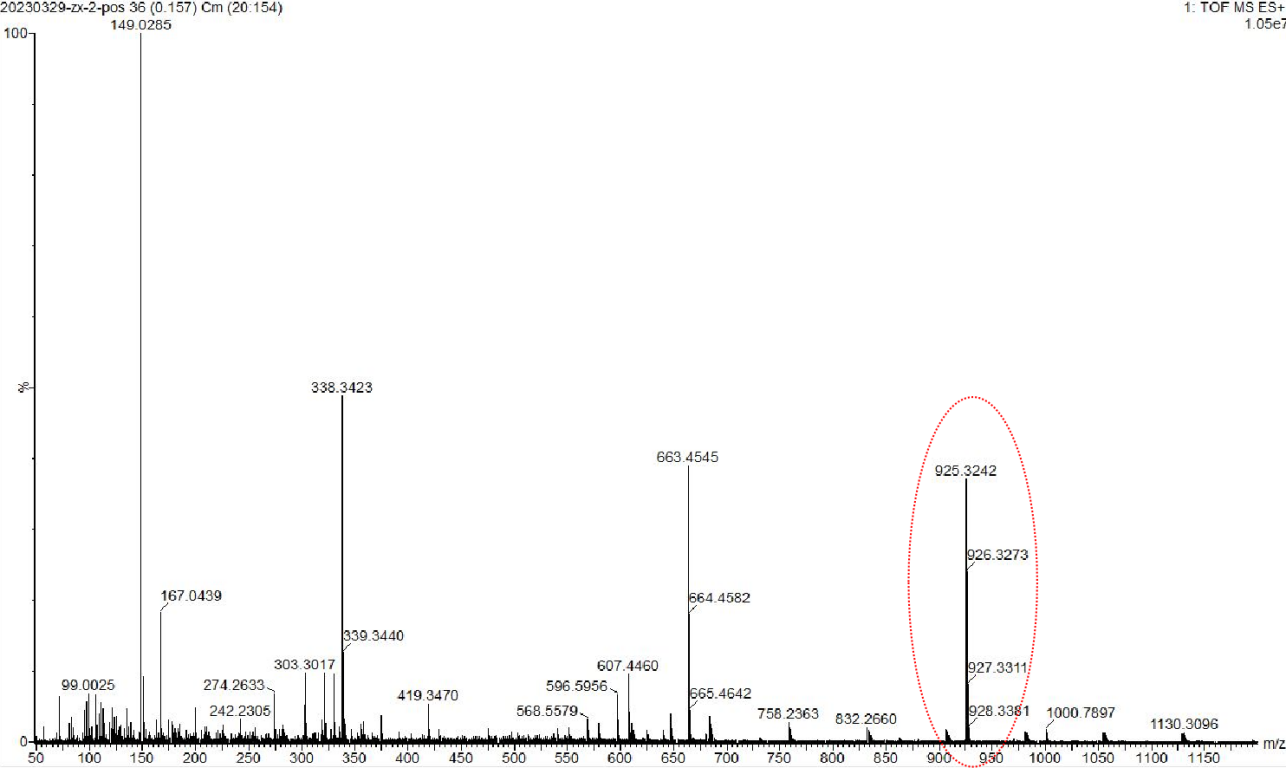


**Figure S5.** Mass spectrum of 2NO_2_-TDI (Exact mass = 924.3159).


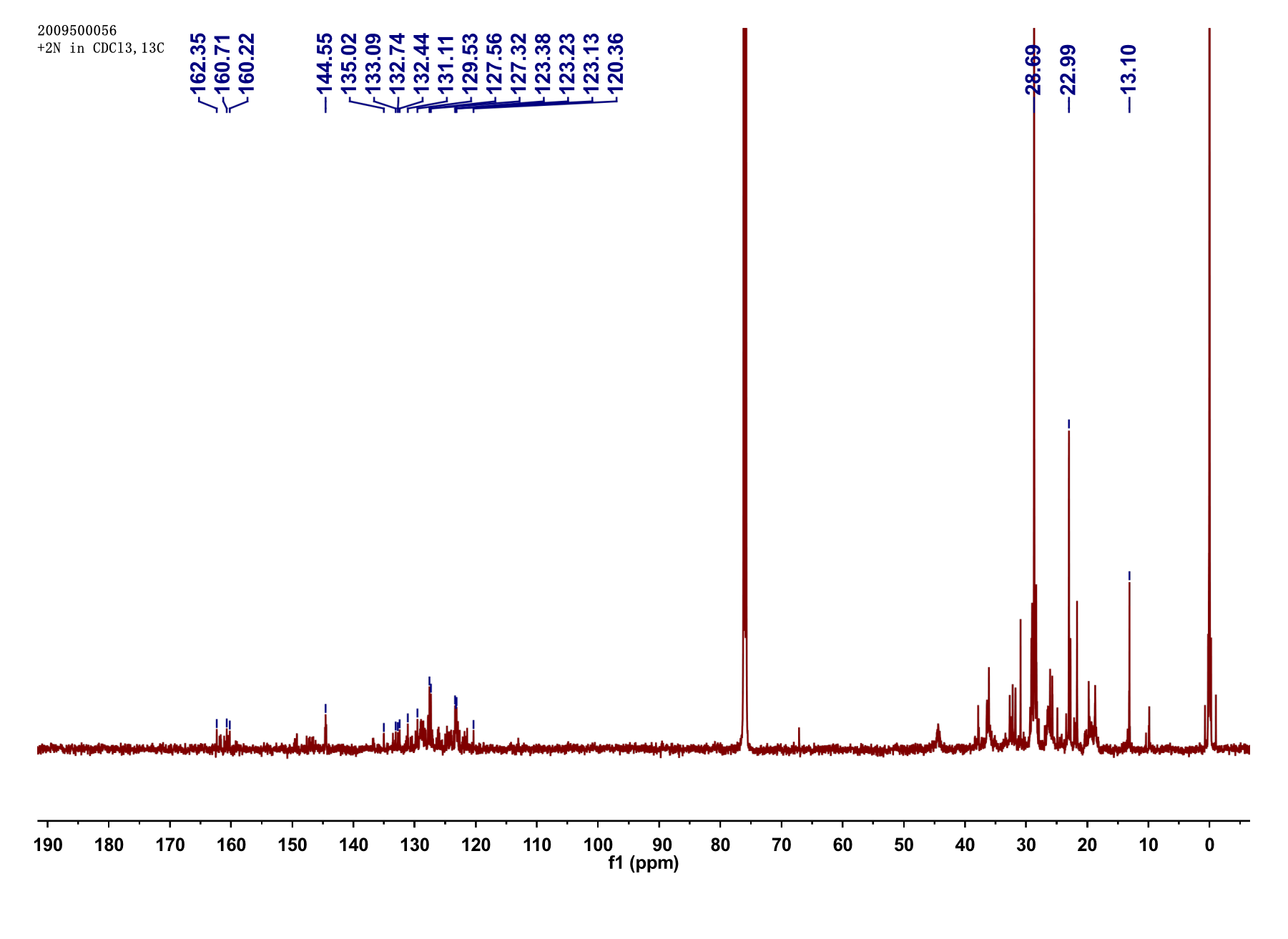


**Figure S6.** ^13^C NMR spectrum of 2NO_2_-TDI (600 MHz, CDCl_3_, 298 K).


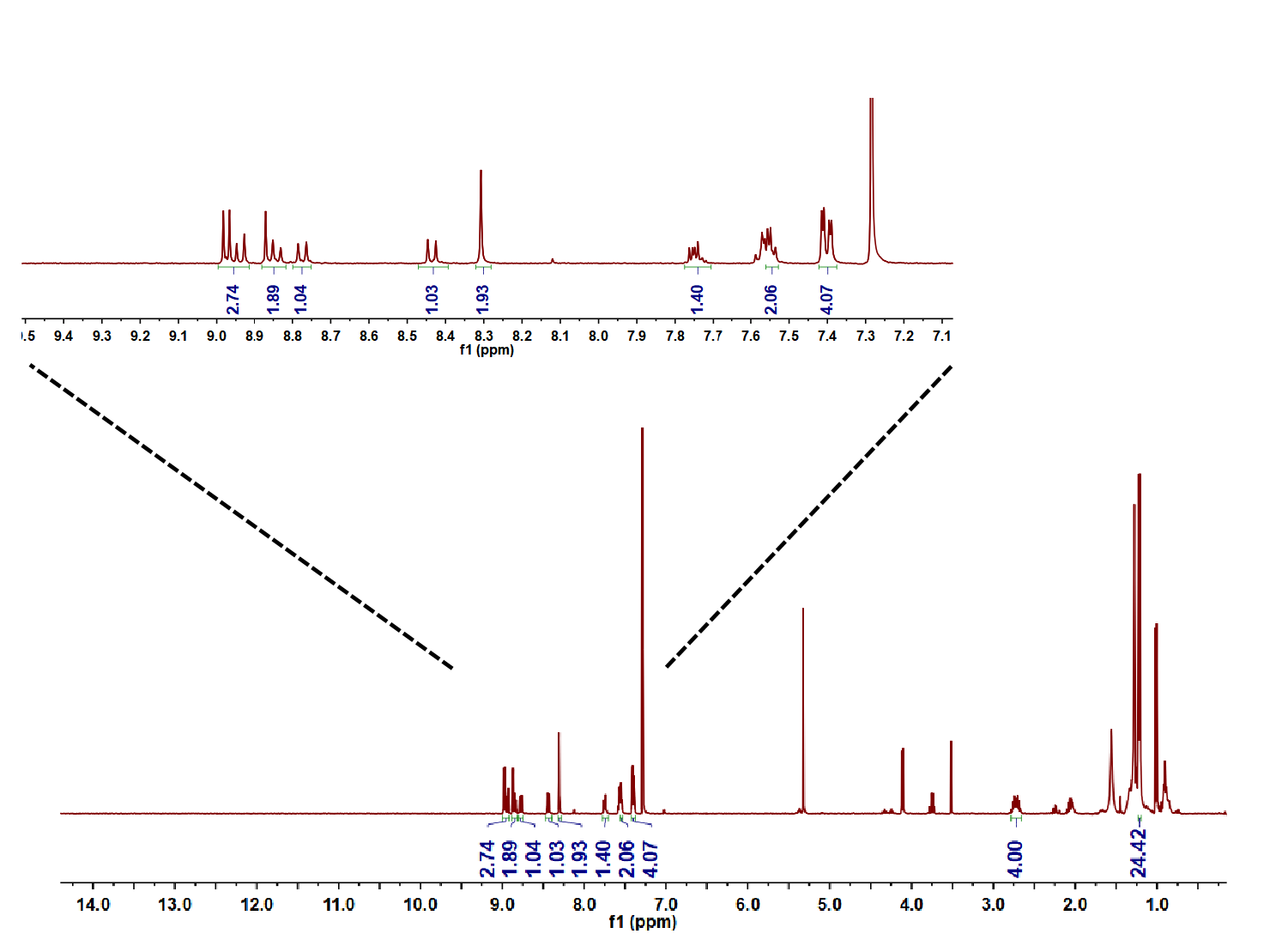


**Figure S7.** ^1^H NMR spectrum of 3NO_2_-TDI (400 MHz, CDCl_3_, 298 K).


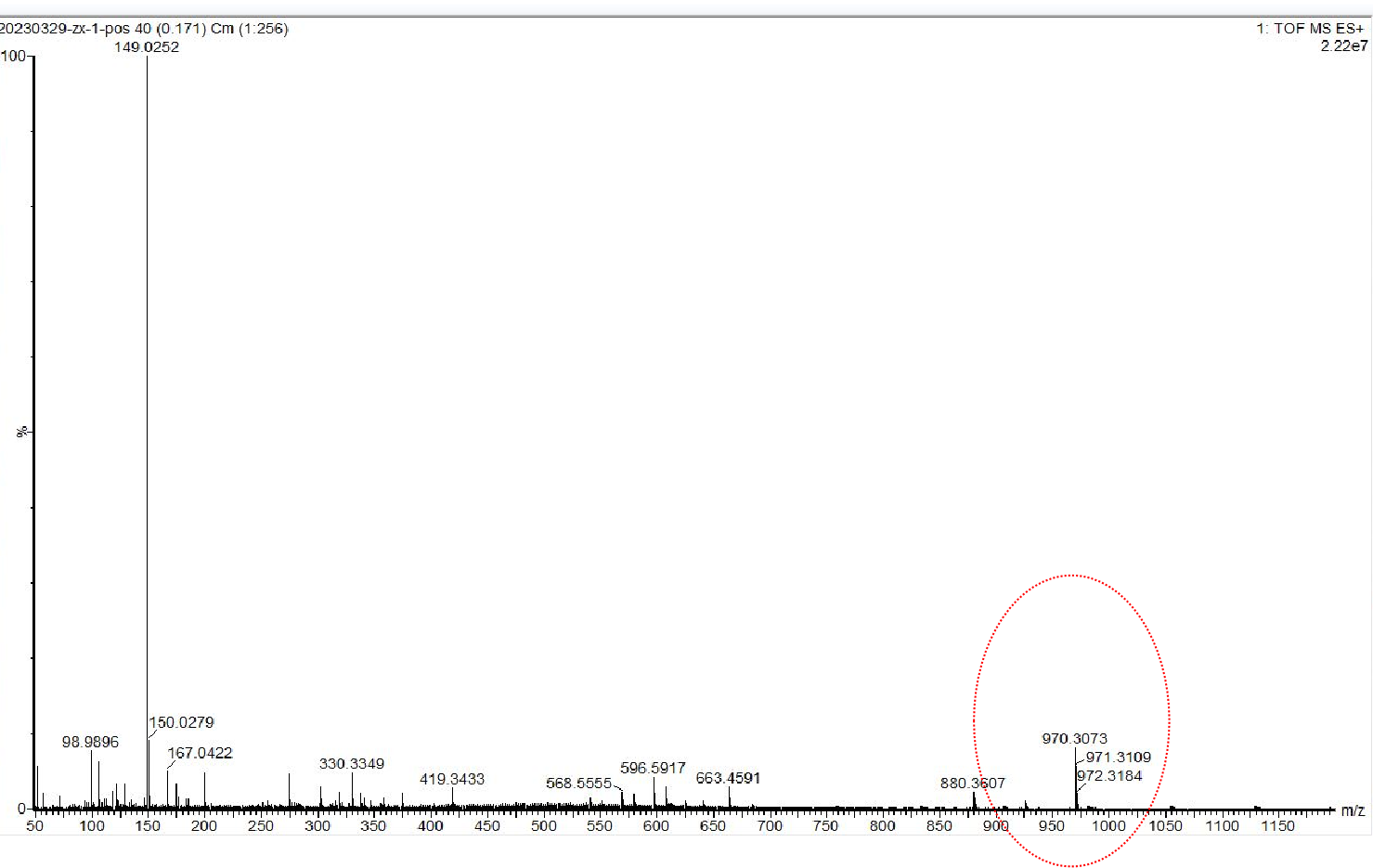


**Figure S8.** Mass spectrum of 3NO_2_-TDI (Exact mass = 969.3010).


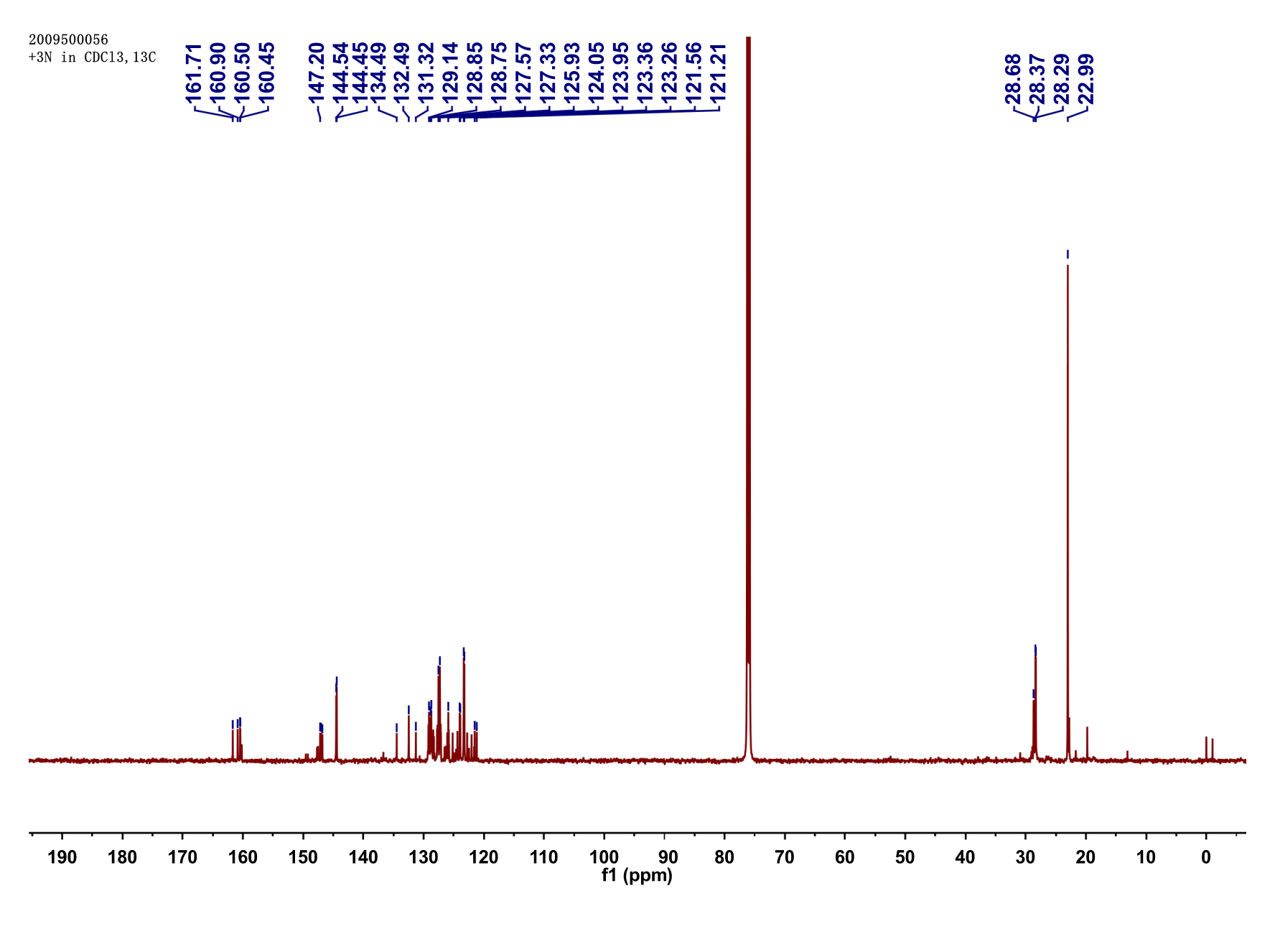


**Figure S9.** ^13^C NMR spectrum of 3NO_2_-TDI (600 MHz, CDCl_3_, 298 K).


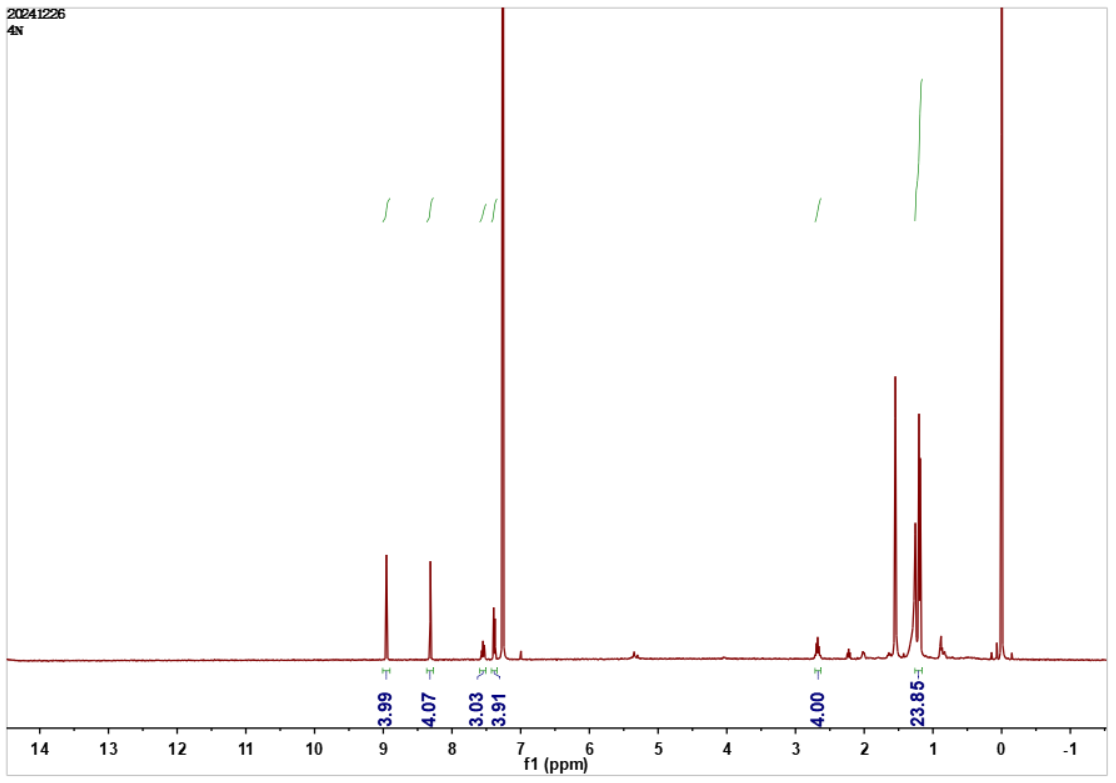


**Figure S10.** ^1^H NMR spectrum of 4NO_2_-TDI (400 MHz, CDCl_3_, 298 K).


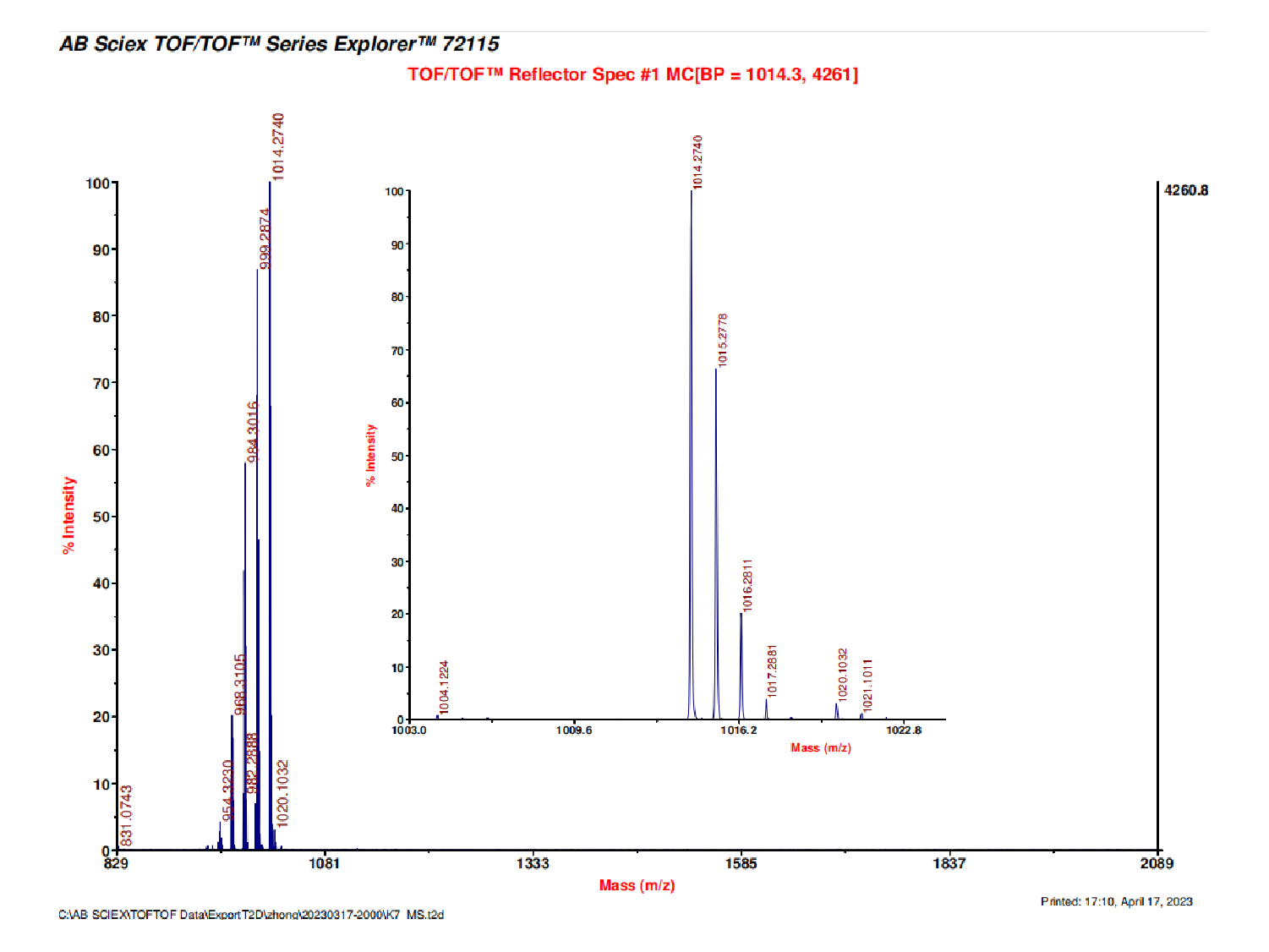


**Figure S11.** Mass spectrum of 4NO_2_-TDI (Exact mass = 1014.2861).


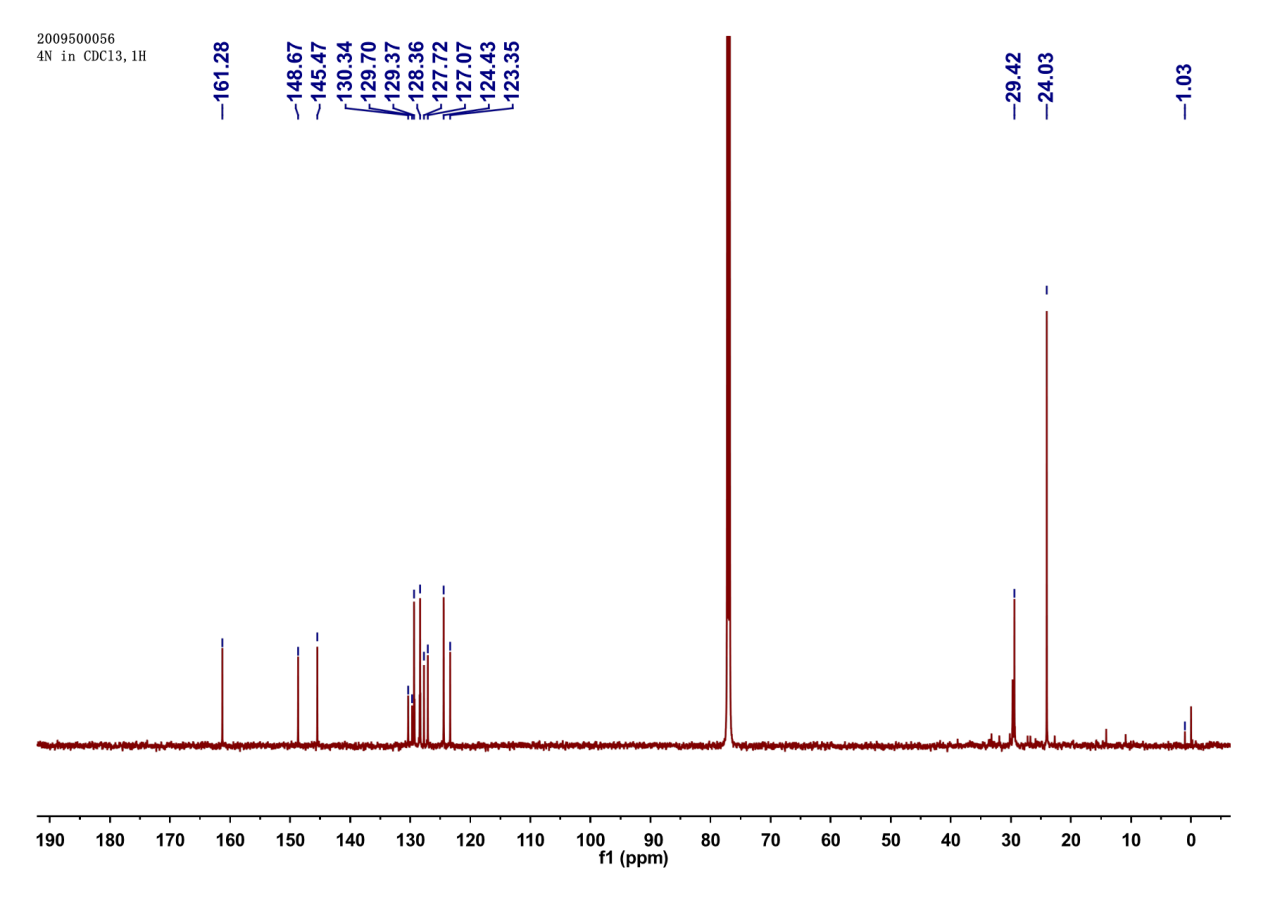


**Figure S12.** ^13^C NMR spectrum of 4NO_2_-TDI (600 MHz, CDCl_3_, 298 K).


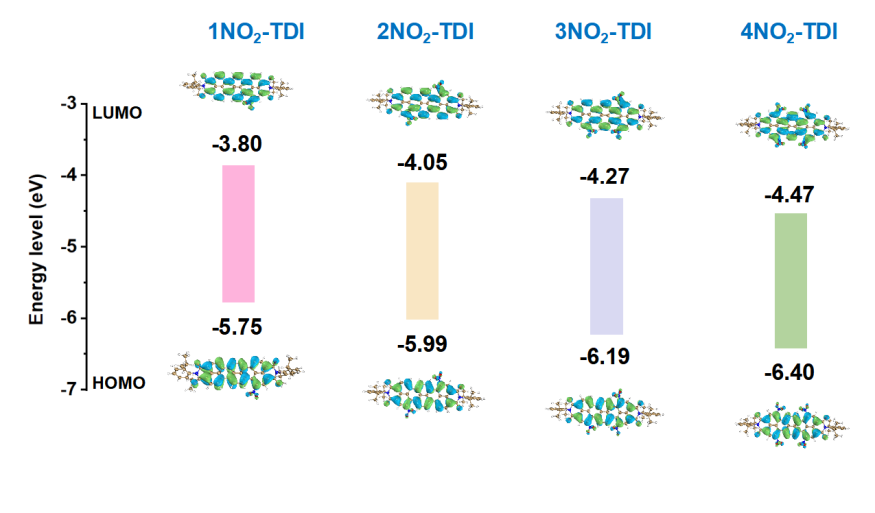


**Figure S13.** DFT computed frontier molecular orbital diagrams with optical band gaps (Eg) of 1-4NO_2_-TDIs. 4NO_2_-TDI shows the lowest LUMO energy (-4.47 eV), indicating highest suscetability to reduction.


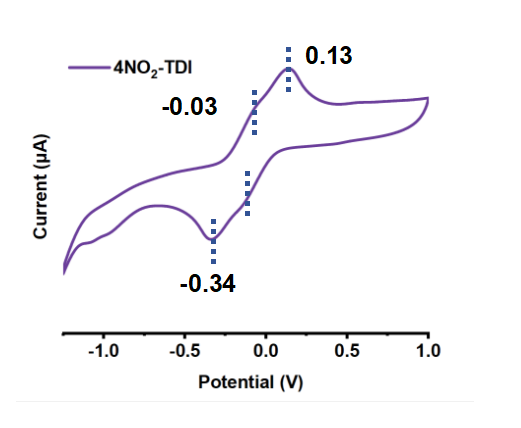


**Figure S14.** Cyclic voltammograms of 4NO_2_-TDI vs. Fc/Fc^+^ in THF. All measurements were conducted in anhydrous THF containing 0.1 M tetrabutylammonium hexafluorophosphate (TBAP) as the supporting electrolyte. The first reversible reduction potentials become progressively more anodic with increasing nitration degree, with 4NO_2_-TDI exhibiting the highest reduction potential, confirming its enhanced electron-accepting capability.

|  | 1NO_2_-TDI | 2NO_2_-TDI | 3NO_2_-TDI | 4NO_2_-TDI |
| --- | --- | --- | --- | --- |
| E^1^_ox_ (V) | -0.71 | -0.37 | -0.41 | -0.34 |
| E^2^_ox_ (V) | -0.59 | -0.24 | -0.27 | -0.14 |
| E^1^_red_ (V) | -0.53 | -0.18 | -0.13 | 0.13 |
| E^2^_red_ (V) | -0.66 | -0.30 | -0.27 | -0.03 |

**Table S1.** Summary of the redox properties of 1-4NO_2_-TDIs. E^1^_ox_, first oxidation potential; E^2^_ox_, second oxidation potential; E^1^_red_, first reduction potential; E^2^_red_, second reduction potential.


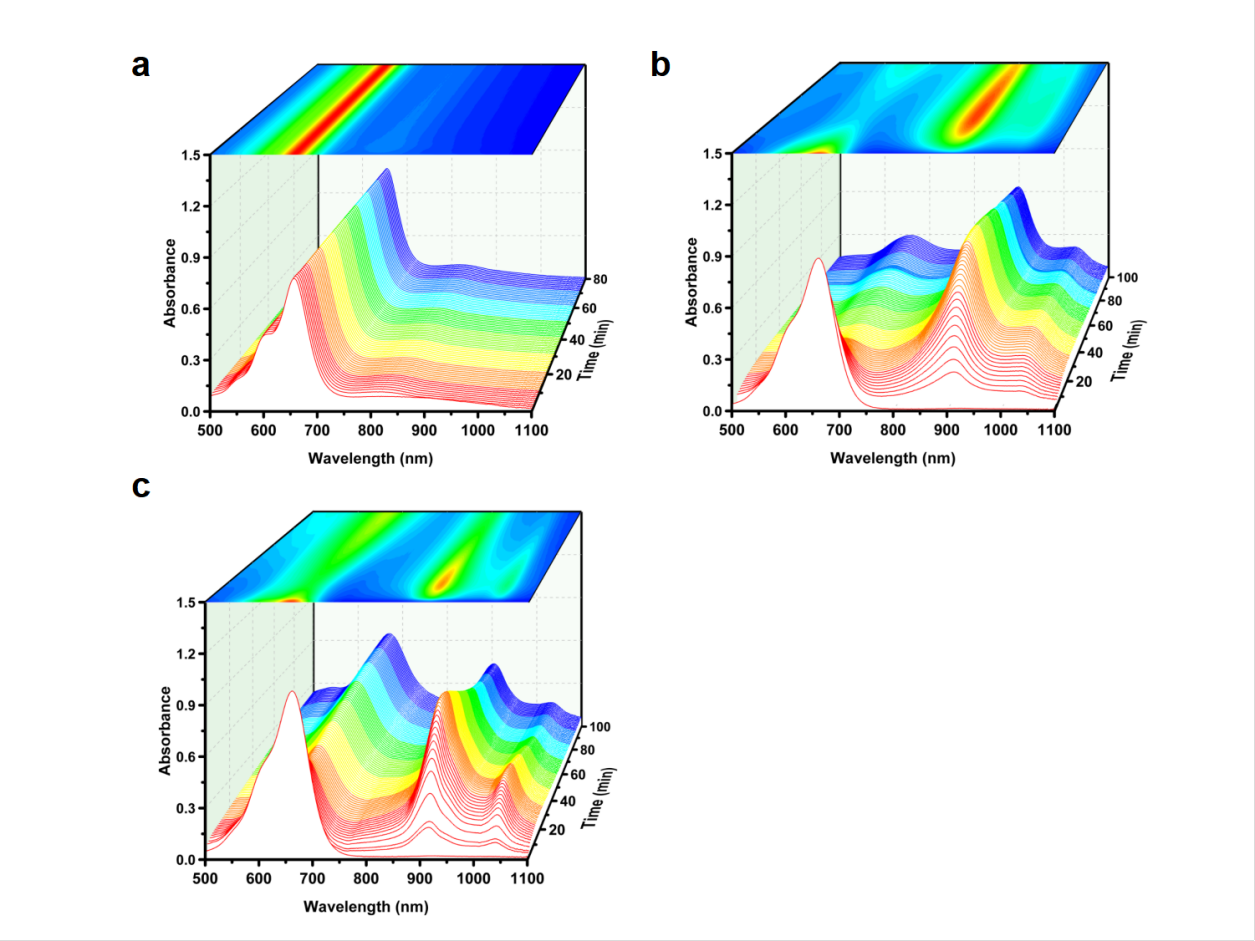


**Figure S15.** Time-dependent UV-vis absorption spectral changes of 1NO_2_-TDI (a), 2NO_2_-TDI (b) and 3NO_2_-TDI (c) (50 μM) after the addition of 7.15 mM triethylamine (TEA) in THF. Upon TEA addition, 2NO_2_-TDI and 3NO_2_-TDI readily formed radical anions with strong absorption in the NIR-I and NIR-II regions (800-1100 nm), while 1NO_2_-TDI showed negligible radical anions formation.


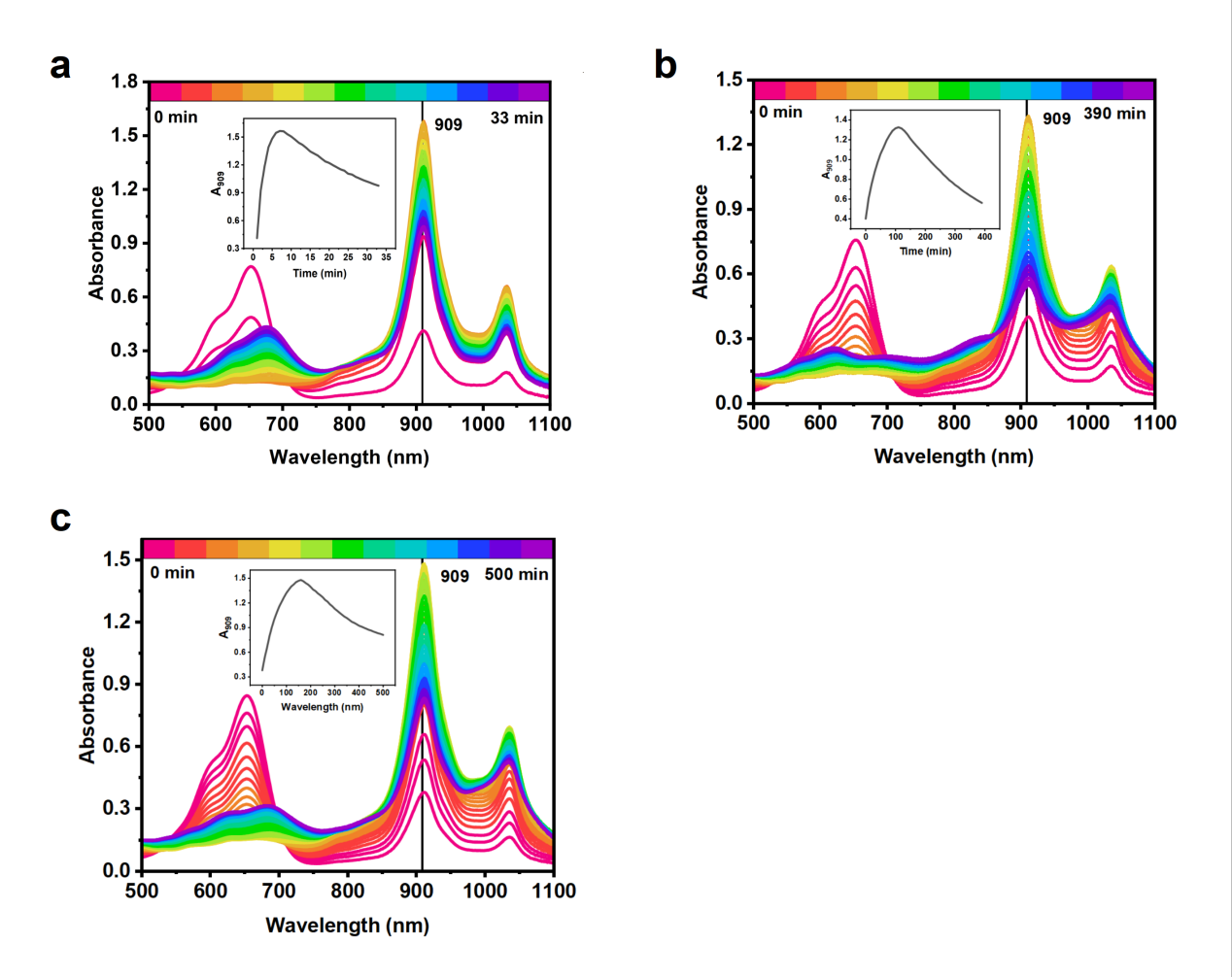


**Figure S16.** Time-dependent UV-vis absorption spectral changes of 4NO_2_-TDI (50 μM) after the addition of 7.15 mM TEA (a), 4 mM GSH (b) and 0.2 mM F127 (c) in THF. Inset: Absorption changes of 4NO_2_-TDI at 909 nm as a function of time. These spectral changes reveal sequential formation of TDI^•-^ (characteristic absorption at 909 nm) followed by its conversion to TDI²⁻ (absorption at 680 nm) and demonstrate a two-step electron transfer process: (TDI + e⁻ → TDI^•-^; TDI^•-^ + e⁻ → TDI²⁻).


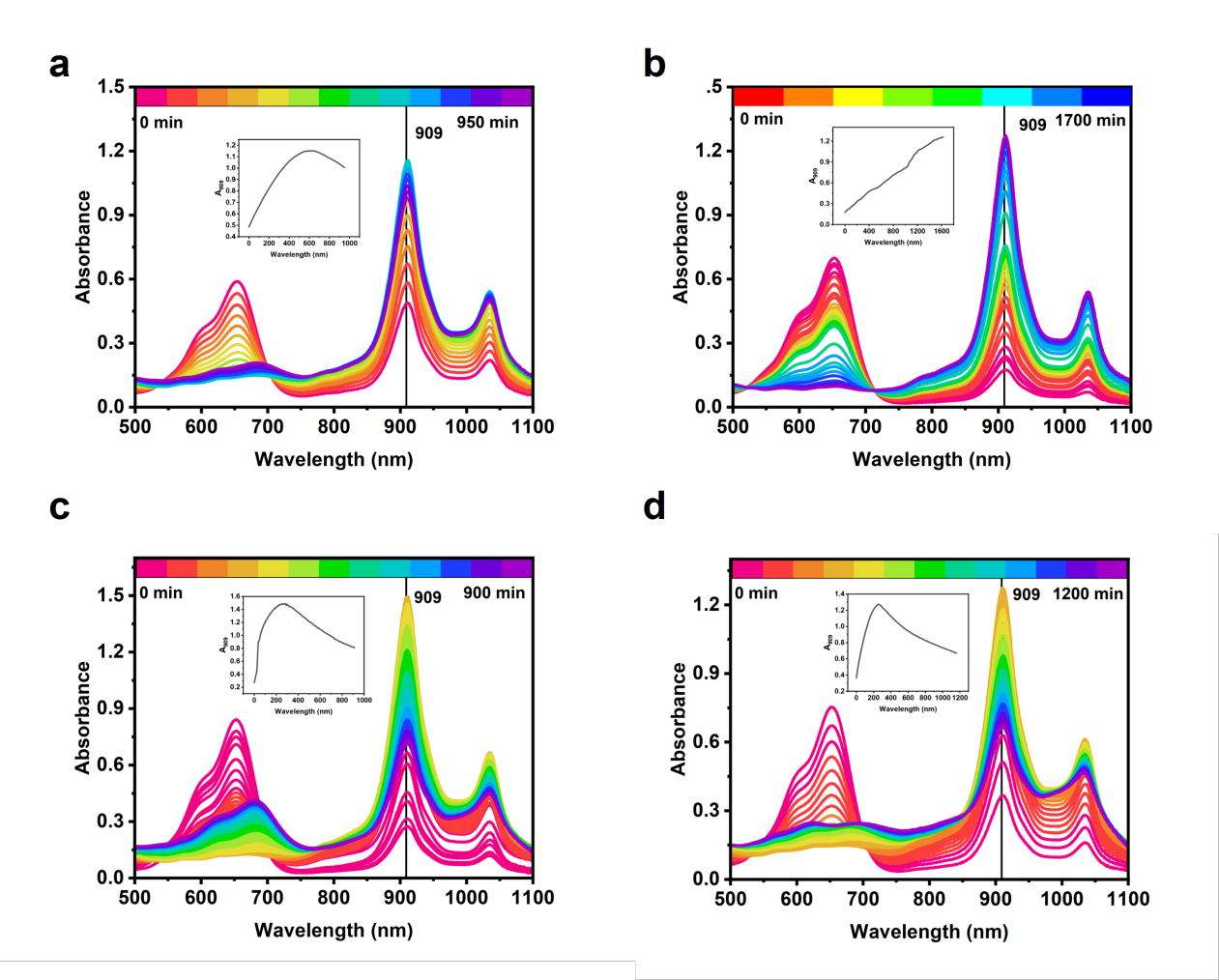


**Figure S17.** Time-dependent UV–vis absorption spectral changes of 2NO_2_-TDI and 3NO_2_-TDI (50 μM) after the addition of 4 mM GSH (a,c) or 0.2 mM F127 (b,d) in THF. Insets: Changes in the absorbance at 909 nm for 2NO_2_-TDI or 3NO_2_-TDI as a function of time.


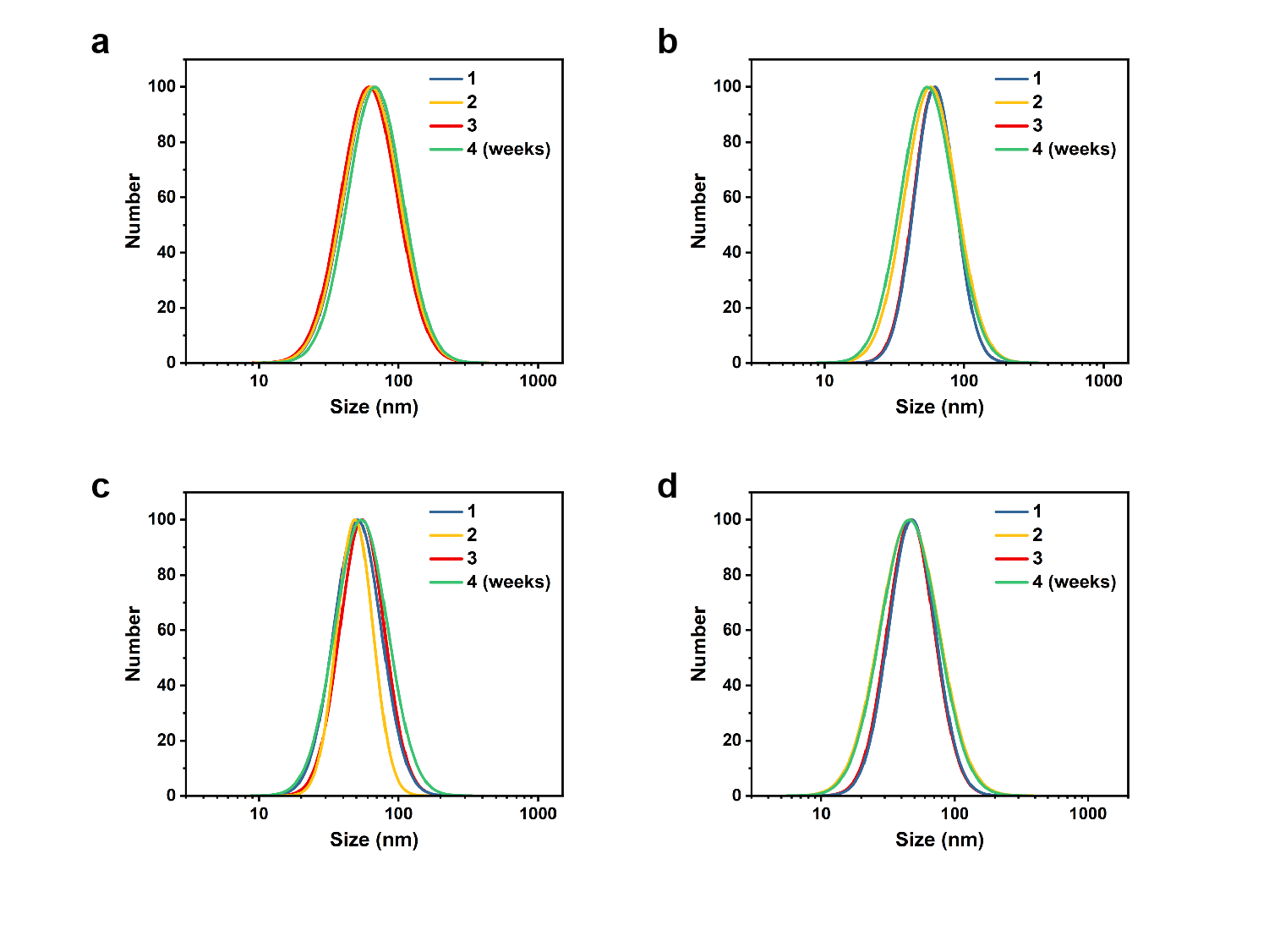


**Figure S18.** The hydrodynamic diameter and the storage stability of (a) 1NO_2_-TDI@F127 NPs (63 nm), (b) 2NO_2_-TDI@F127 NPs (55 nm), (c) 3NO_2_-TDI@F127 NPs (52 nm), (d) 4NO_2_-TDI@F127 NPs (namely TDI^•^**⁻** NPs, 48 nm).


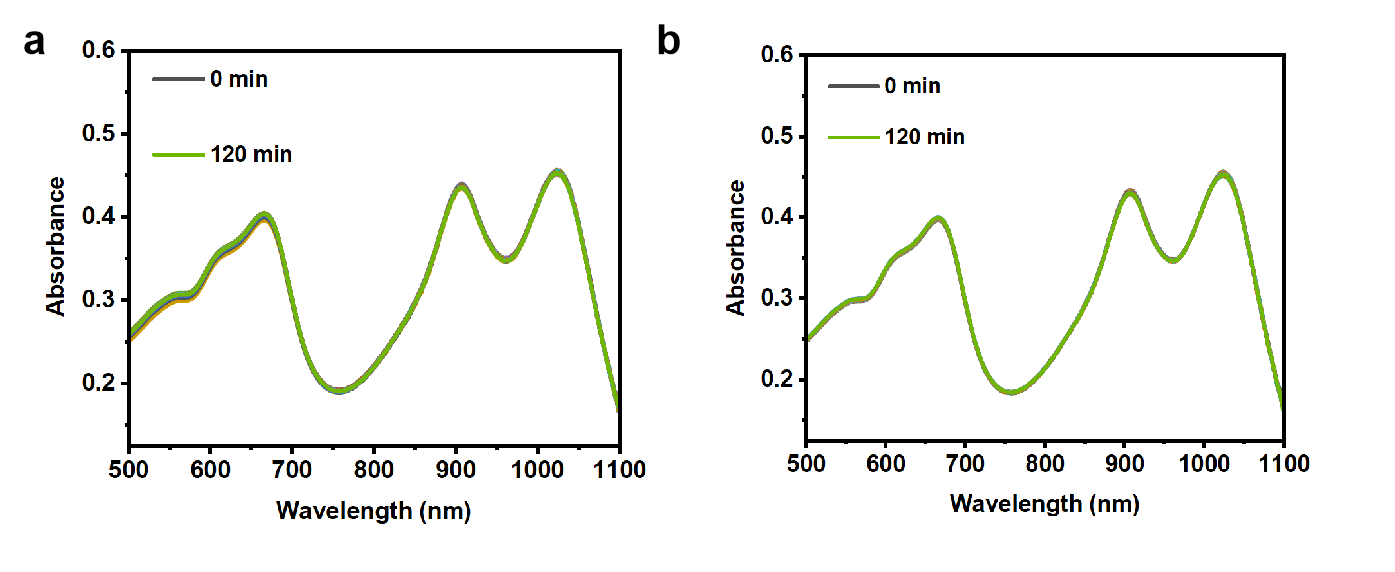


**Figure S19.** Time-dependent UV-vis absorption spectral changes of TDI^•-^ NPs (50 μM) under an nitrogen (a) or oxygen atmosphere (b).


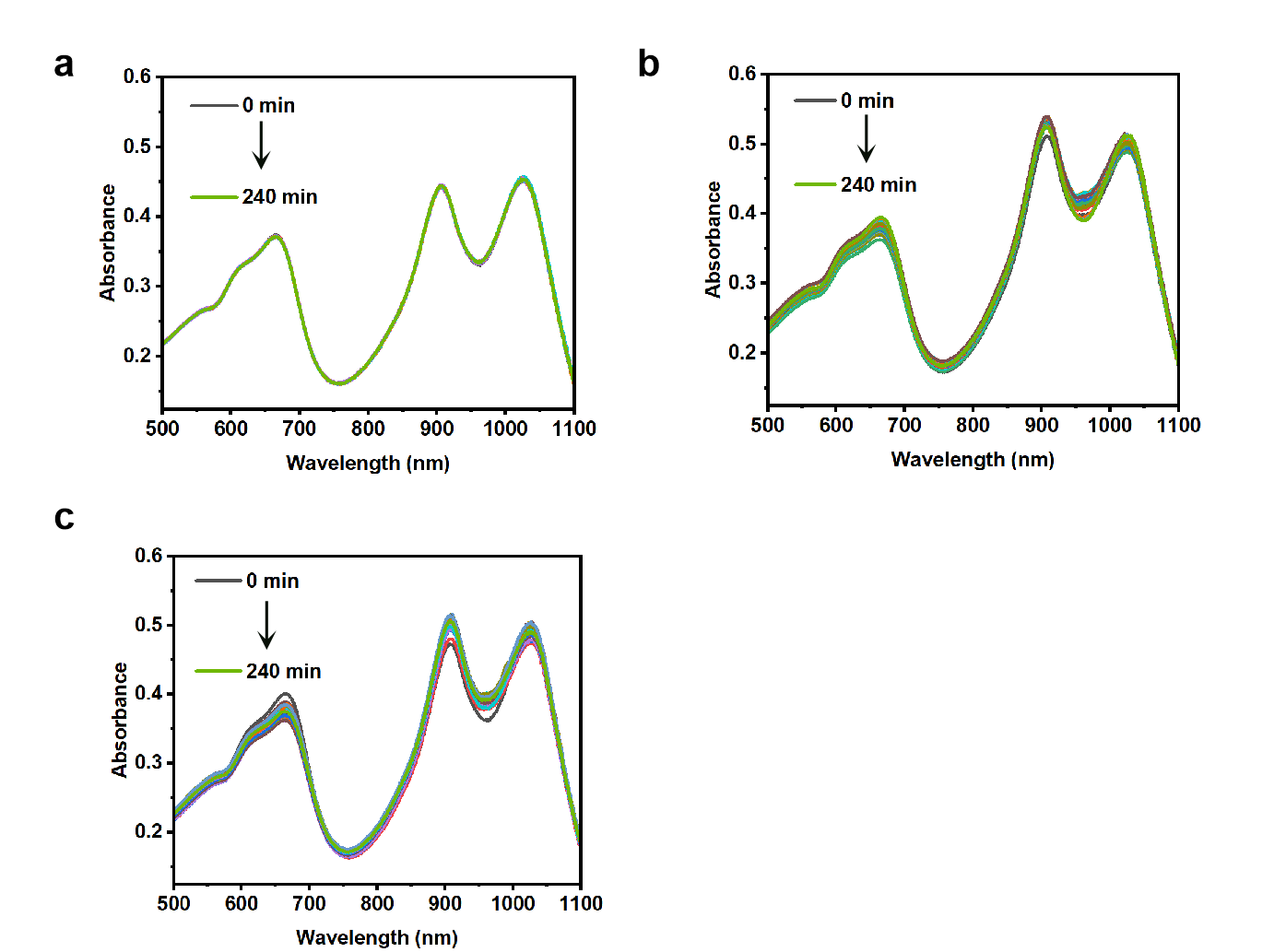


**Figure S20.** Time-dependent UV-vis absorption spectral changes of (a) TDI^•-^ NPs (50 μM) at 25 ℃, (b) 60 ℃, and (c) 80 ℃.


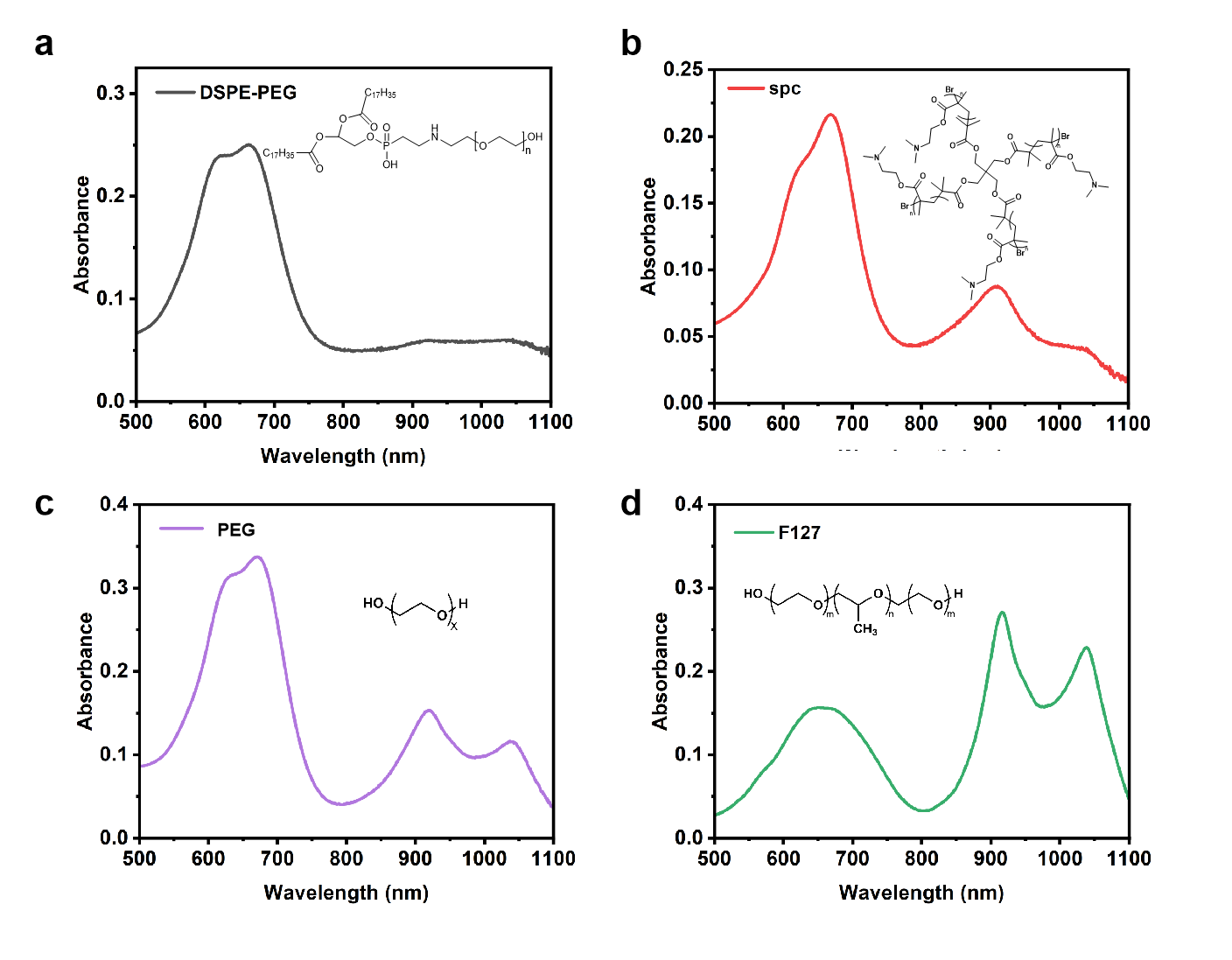


**Figure S21.** UV-visible absorption spectra of 4NO_2_-TDI encapsulated with different surfactants: 1,2-distearoyl-sn-glycero-3-phosphoethanolamine-polyethylene glycol (DSPE-PEG) (a), spc (b), polyethylene glycol (PEG) (c) and F127 (d). Inset: chemical structures of each surfactant.


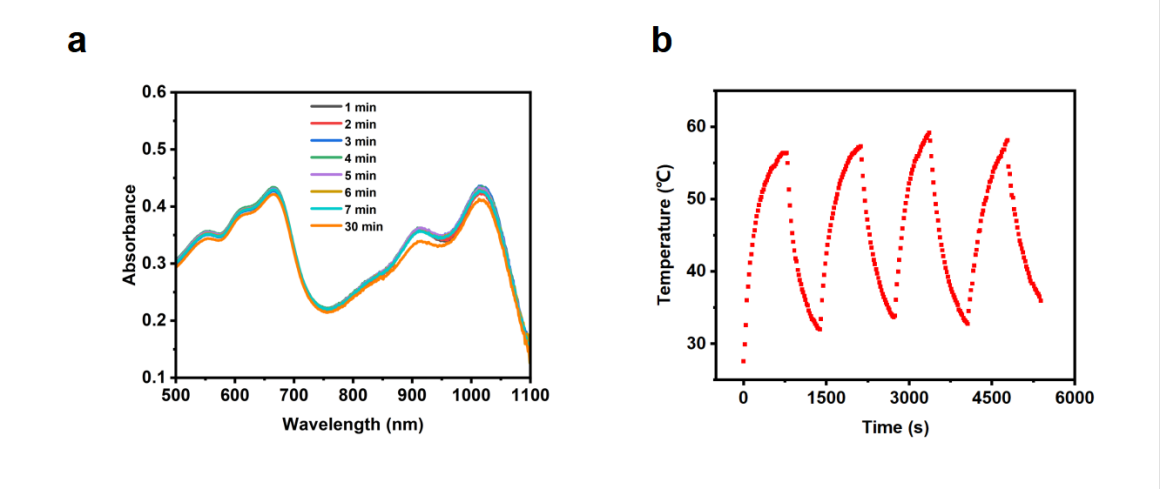


**Figure S22.** (a) Time-dependent UV-vis absorption spectral changes of TDI^•-^ NPs (50 μM) under 1064 nm laser irradiation (0.5 W/cm²). (b) Multiple heating-cooling cycles of TDI^•-^ NPs (50 μM) under 1064 nm laser irradiation (1 W/cm²).


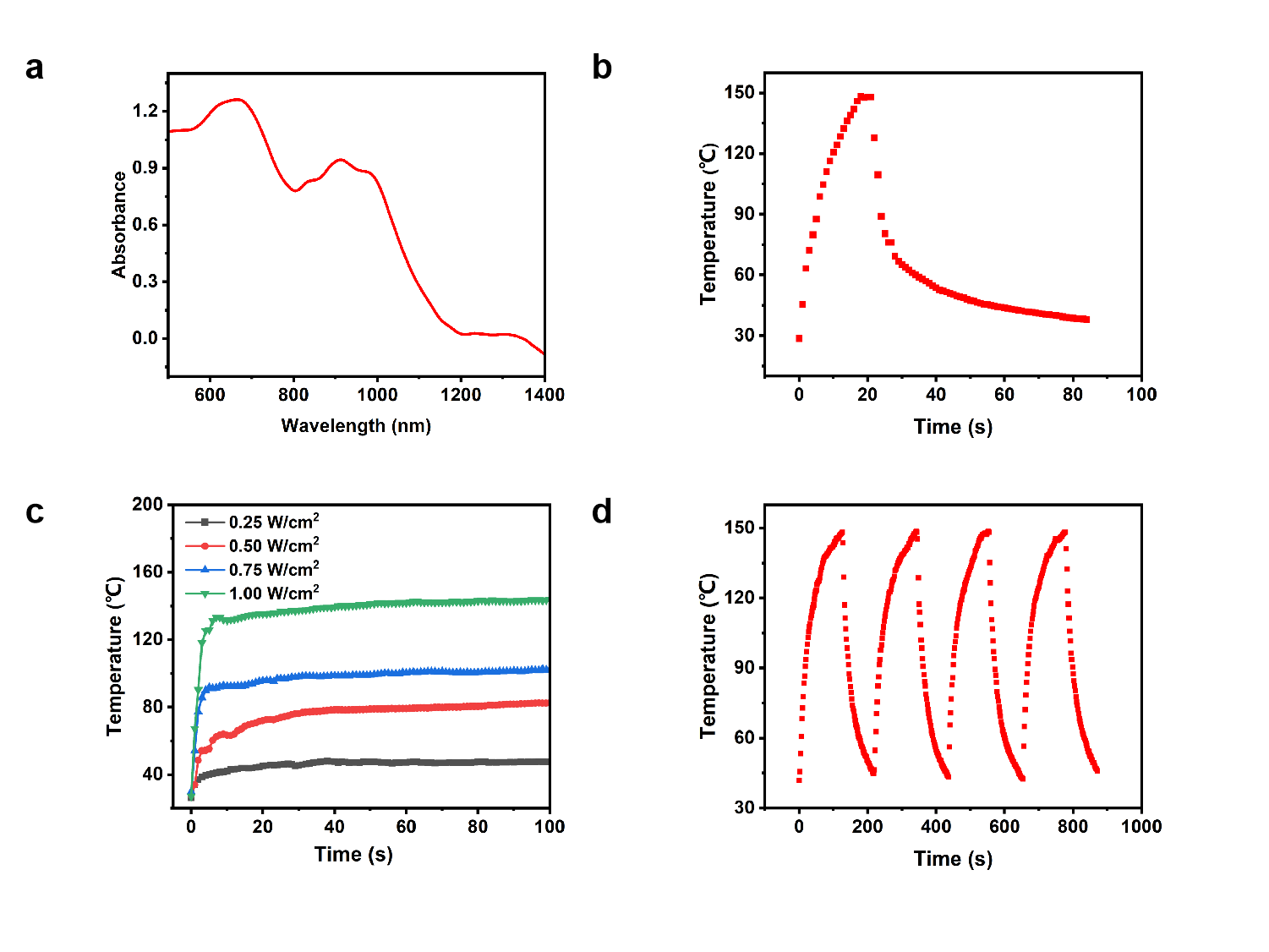


**Figure S23.** Photothermal performance of lyophilized TDI^•-^ NPs powder. (a) UV-vis absorption spectrum of TDI^•-^ NPs in the solid. (b) Heating-cooling curve of TDI^•-^ NPs under 1064 nm laser irradiation (1 W/cm^2^) in the solid state. (c) Heating curves of TDI^•-^ NPs in the solid state under 1064 nm laser irradiation at different power densities. (d) Multiple heating-cooling cycles of TDI^•-^ NPs in the solid state under 1064 nm laser irradiation (1 W/cm^2^).


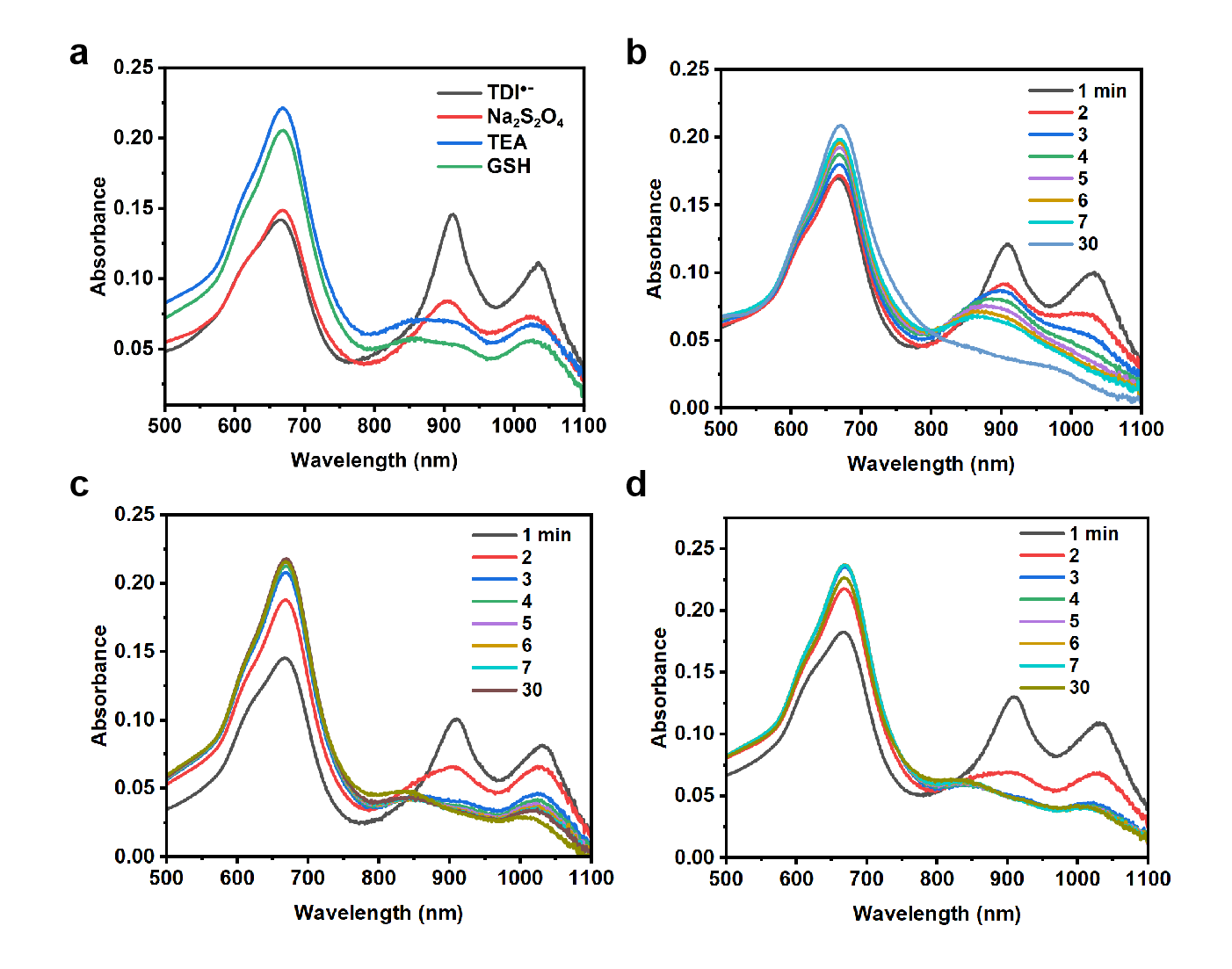


**Figure S24.** Response of TDI^•-^ NPs (20 μM) to different reducing agents (a). Time-dependent UV-vis absorption spectral changes of TDI^•-^ NPs (20 μM) upon addition of 0.1 mM GSH (b), 1 mM GSH (c), and 10 mM GSH (d).


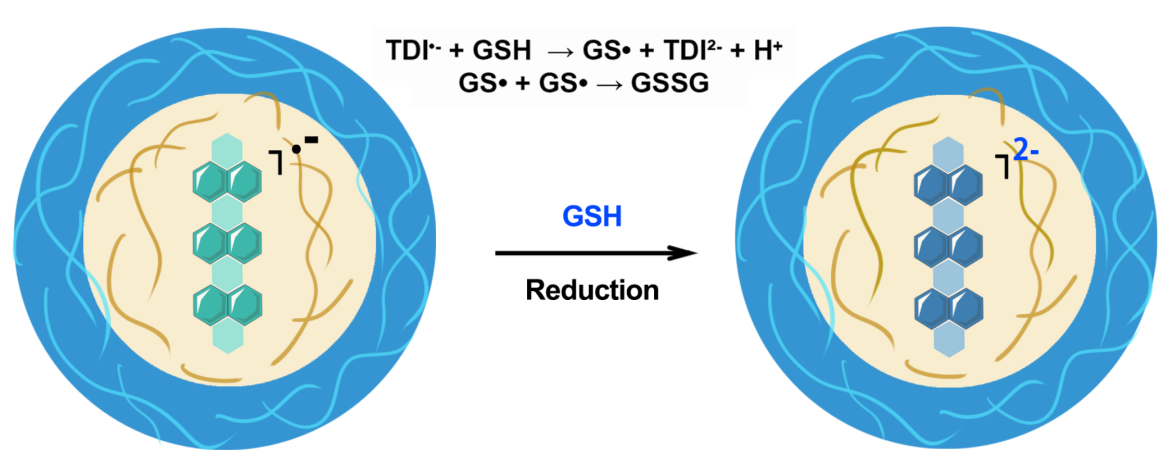


****Scheme S2**.** The reaction between TDI^•-^ NPs and GSH.


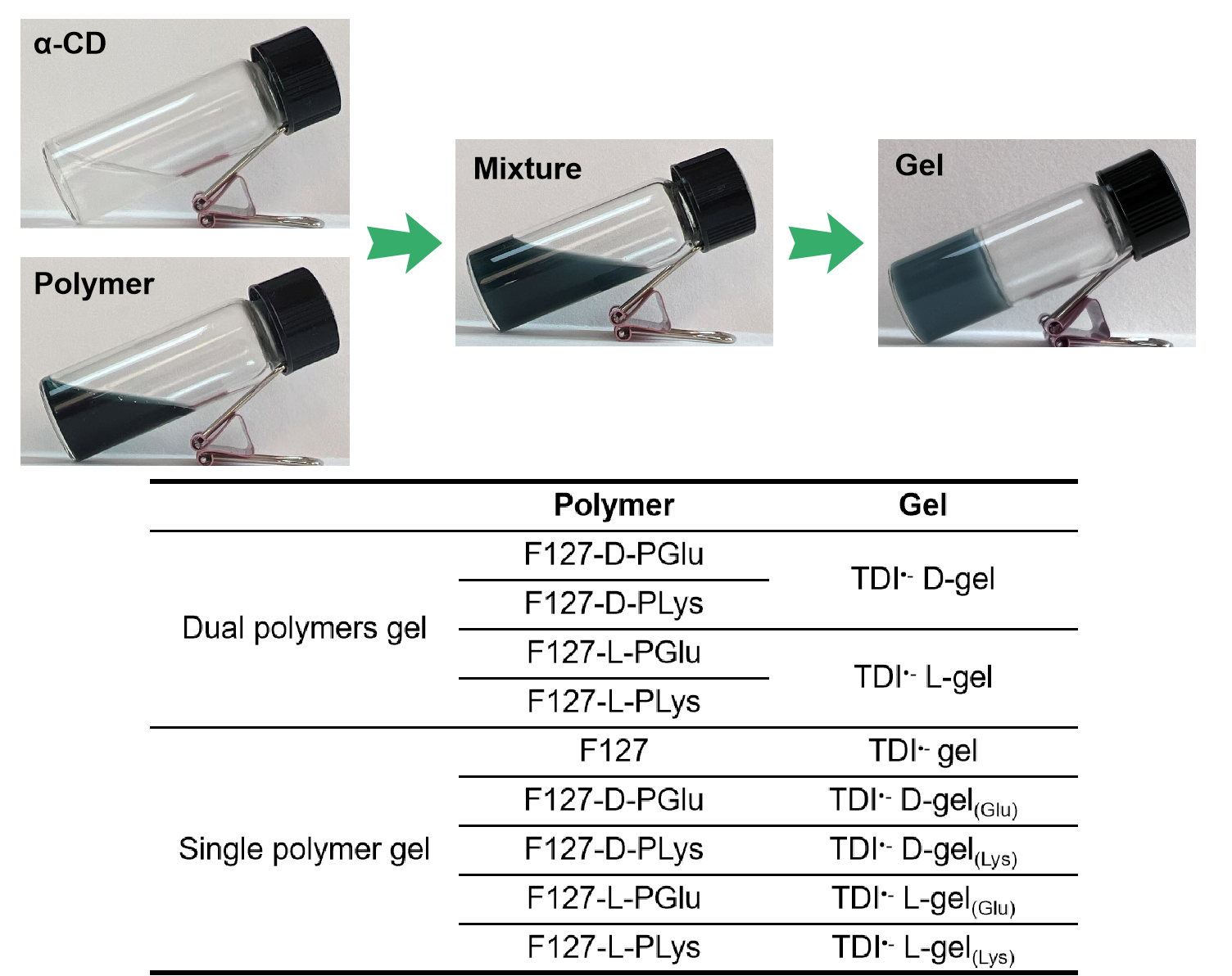


**Figure S25.** Photographic record of hydrogel fabrication.


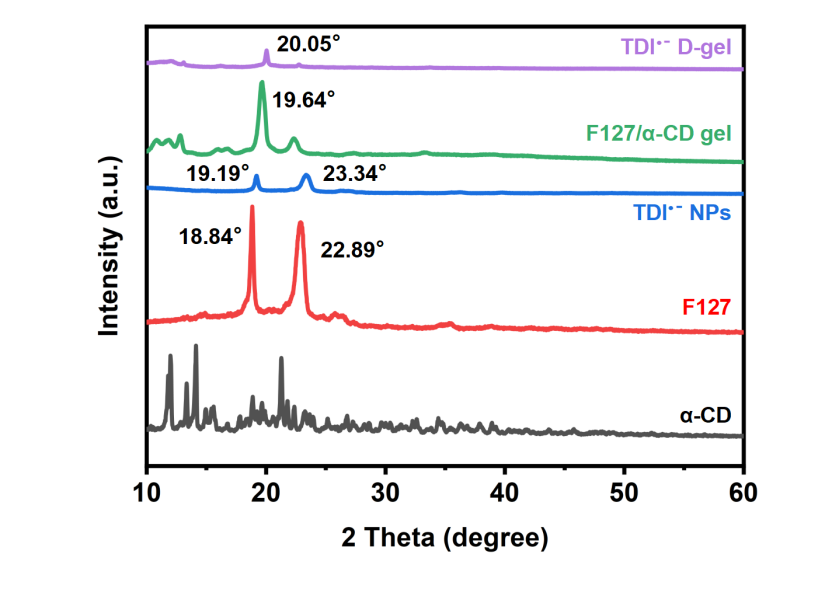


**Figure S26.** XRD Spectra of α-CD, F127, TDI^•-^ NPs, F127/α-CD gel and TDI^•-^ D-gel. The peak at 20.05° represents the interaction between F127 and α-CD, confirming the formation of the gel.

| Sample | α-CD (mg/mL) | F127 (mg/mL) | α-CD/F127 | Gelation time (h) | State | Phase transition temperature (℃) |
| --- | --- | --- | --- | --- | --- | --- |
| 1 | 10 | 15 | 5.8 | - | Sol | - |
| 2 | 20 | 15 | 11.6 | - | Sol | - |
| 3 | 30 | 15 | 17.4 | - | Sol | - |
| 4 | 40 | 15 | 23.2 | - | Precipitate | - |
| 5 | 50 | 15 | 29.1 | 8 | Gel | 45 |
| 6 | 60 | 15 | 34.9 | 5 | Gel | 50 |
| 7 | 70 | 15 | 40.7 | 4 | Gel | 50 |
| 8 | 80 | 15 | 46.6 | 3 | Gel | 55 |
| 9 | 90 | 15 | 52.4 | 0.5 | Gel | 55 |

**Table S2.** Gelation ability and phase-transition temperatures of different ratios of α-CD and F127.


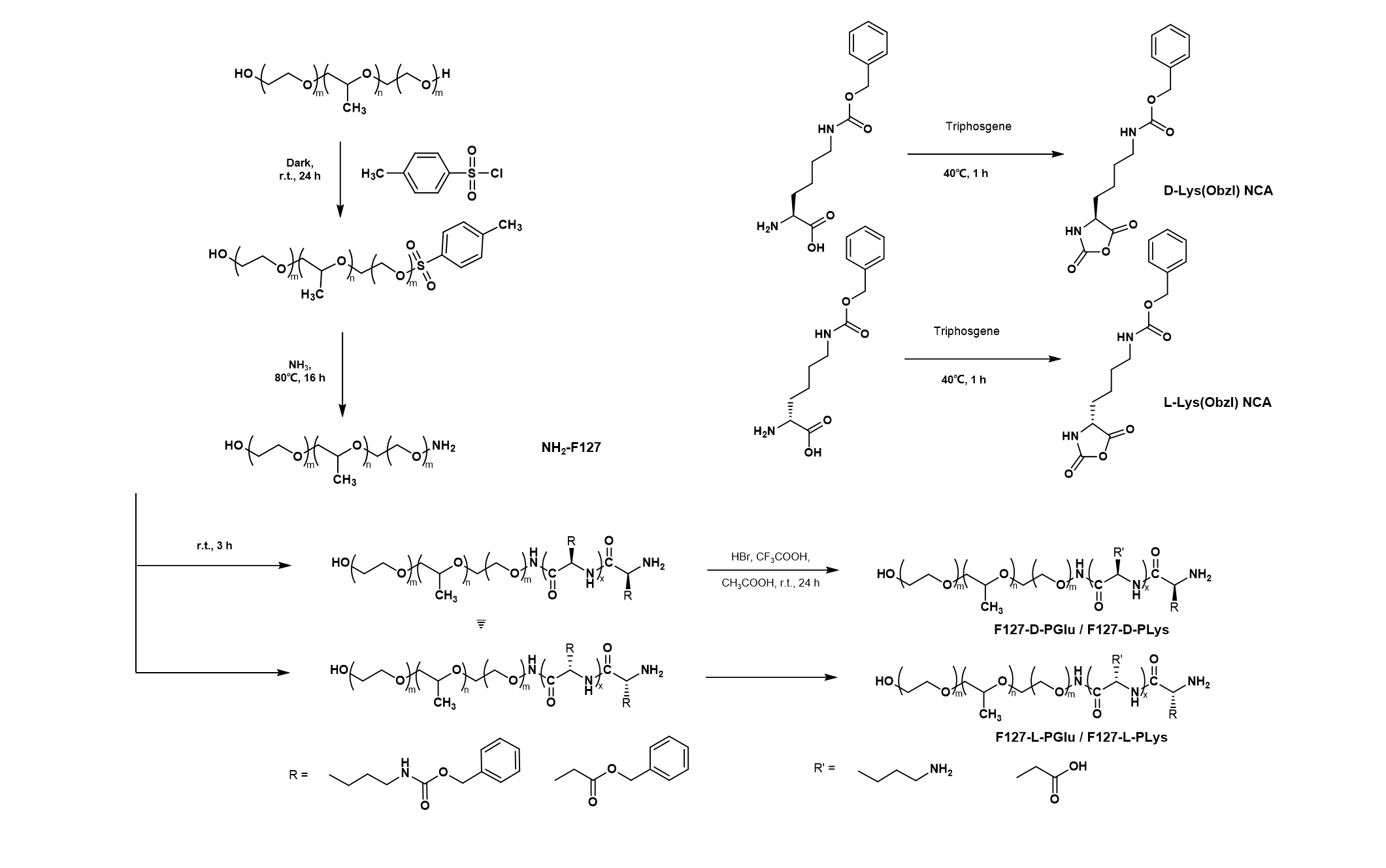


**Scheme S3.** Synthetic routes toward D-Lys(Obzl) NCA, L-Lys(Obzl) NCA, F127-L-PLys, F127-D-PLys, F127-L-PGlu and F127-D-PGlu.


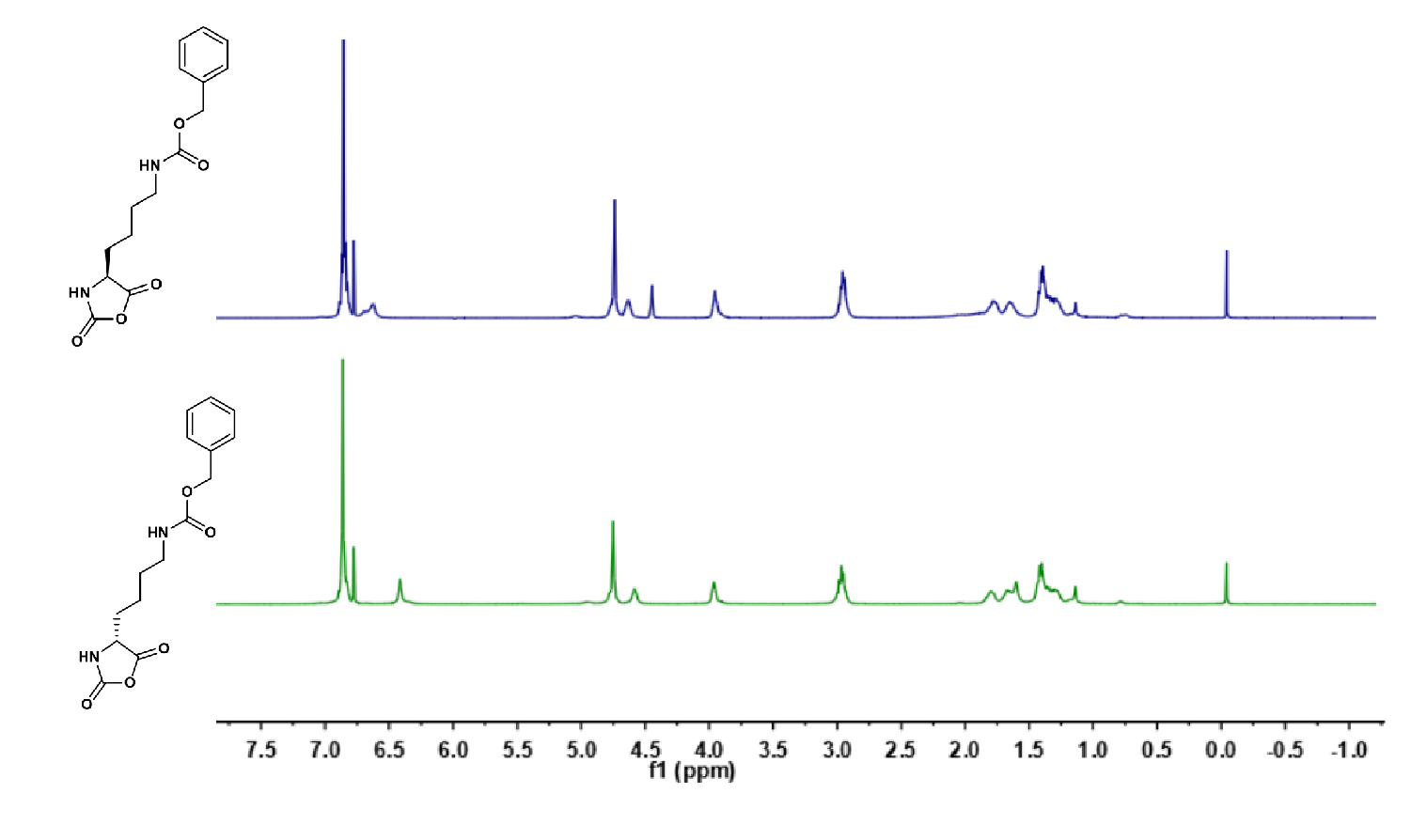


**Figure S27.** ^1^H NMR spectra of D-Lys(Obzl) NCA (top) and L-Lys(Obzl) NCA (bottom) (400 MHz, CDCl_3_, 298 K).


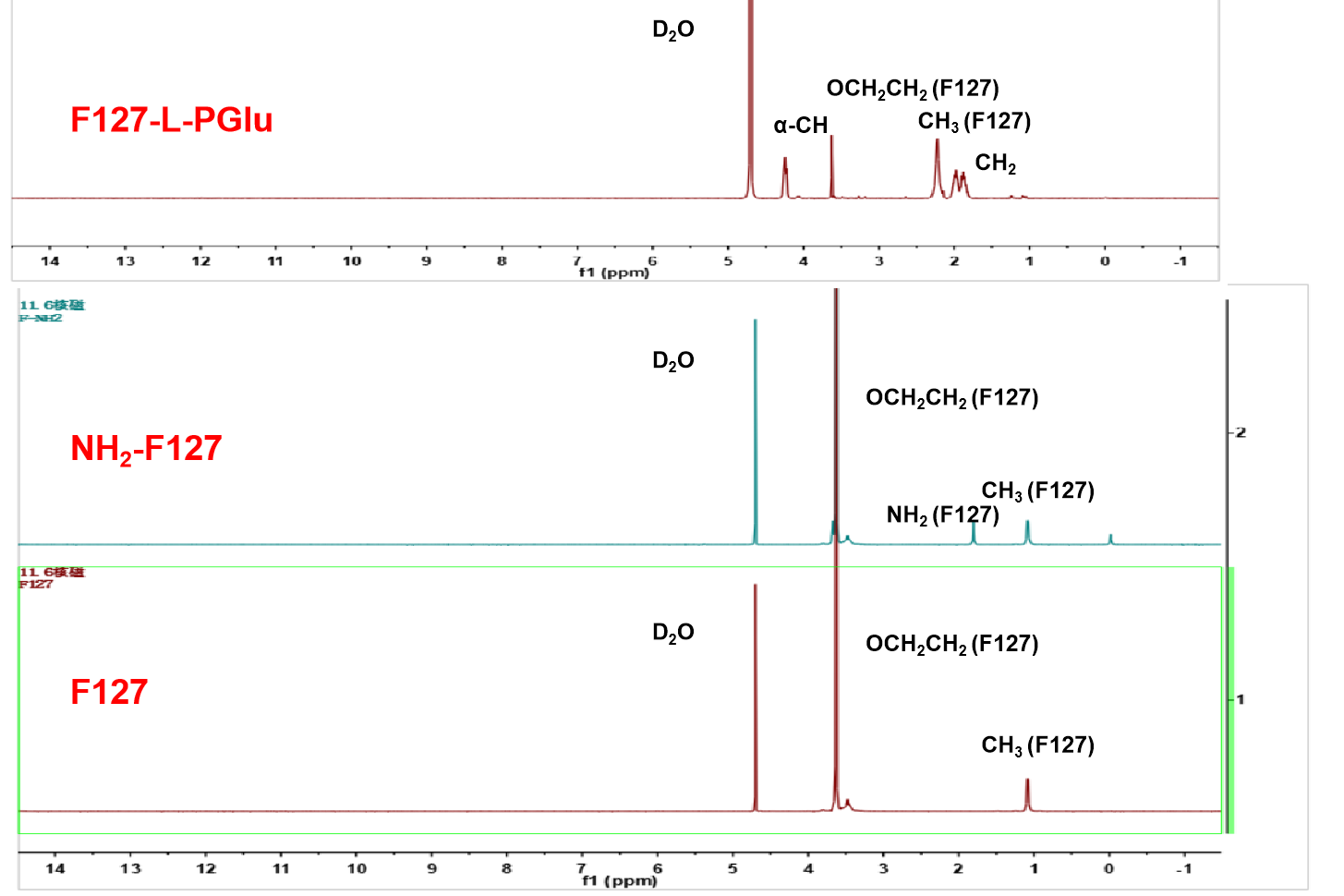


**Figure S28.** ^1^H NMR spectra of F127-L-PGlu, NH_2_-F127 and F127 (400 MHz, CDCl_3_, 298 K).


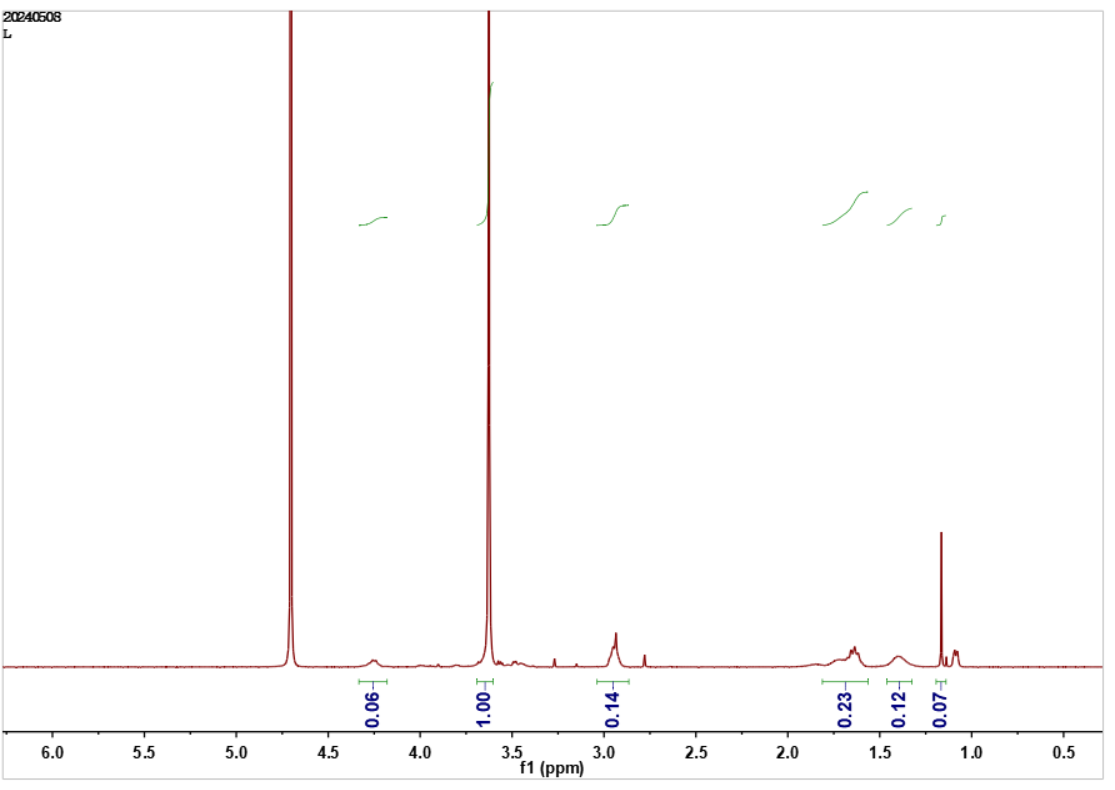


**Figure S29.** ^1^H NMR spectra of F127-L-PLys (400 MHz, CDCl_3_, 298 K).


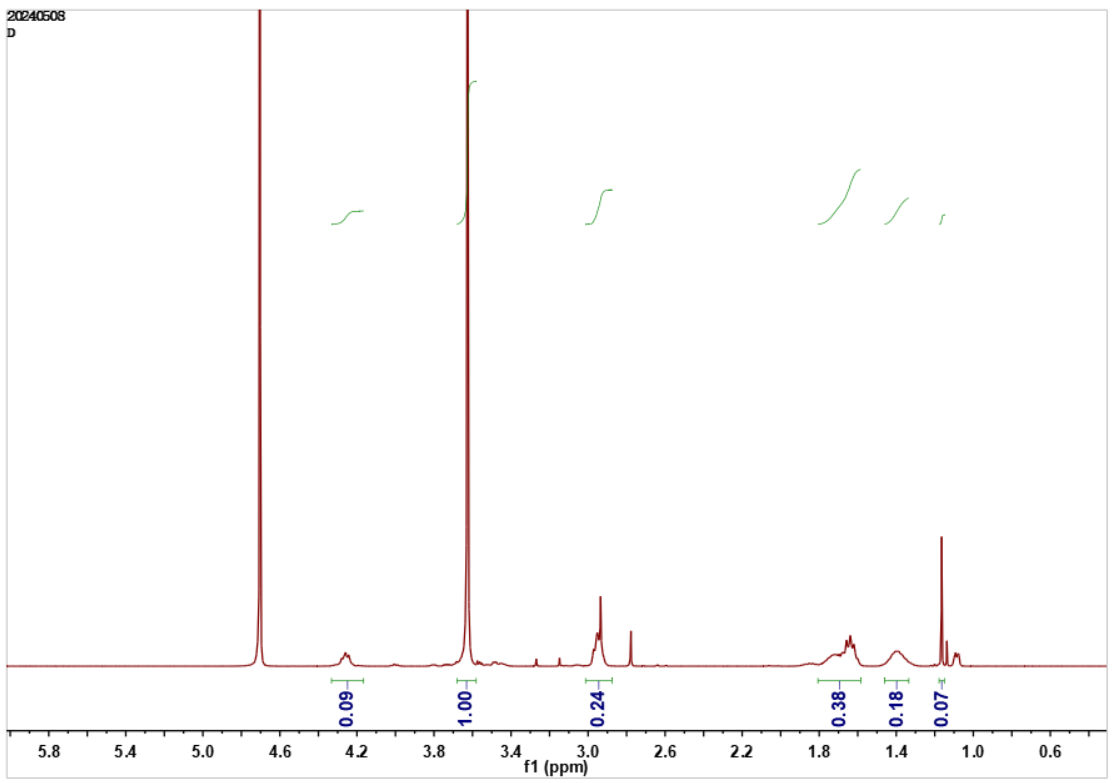


**Figure S30.** ^1^H NMR spectra of F127-D-PLys (400 MHz, CDCl_3_, 298 K).


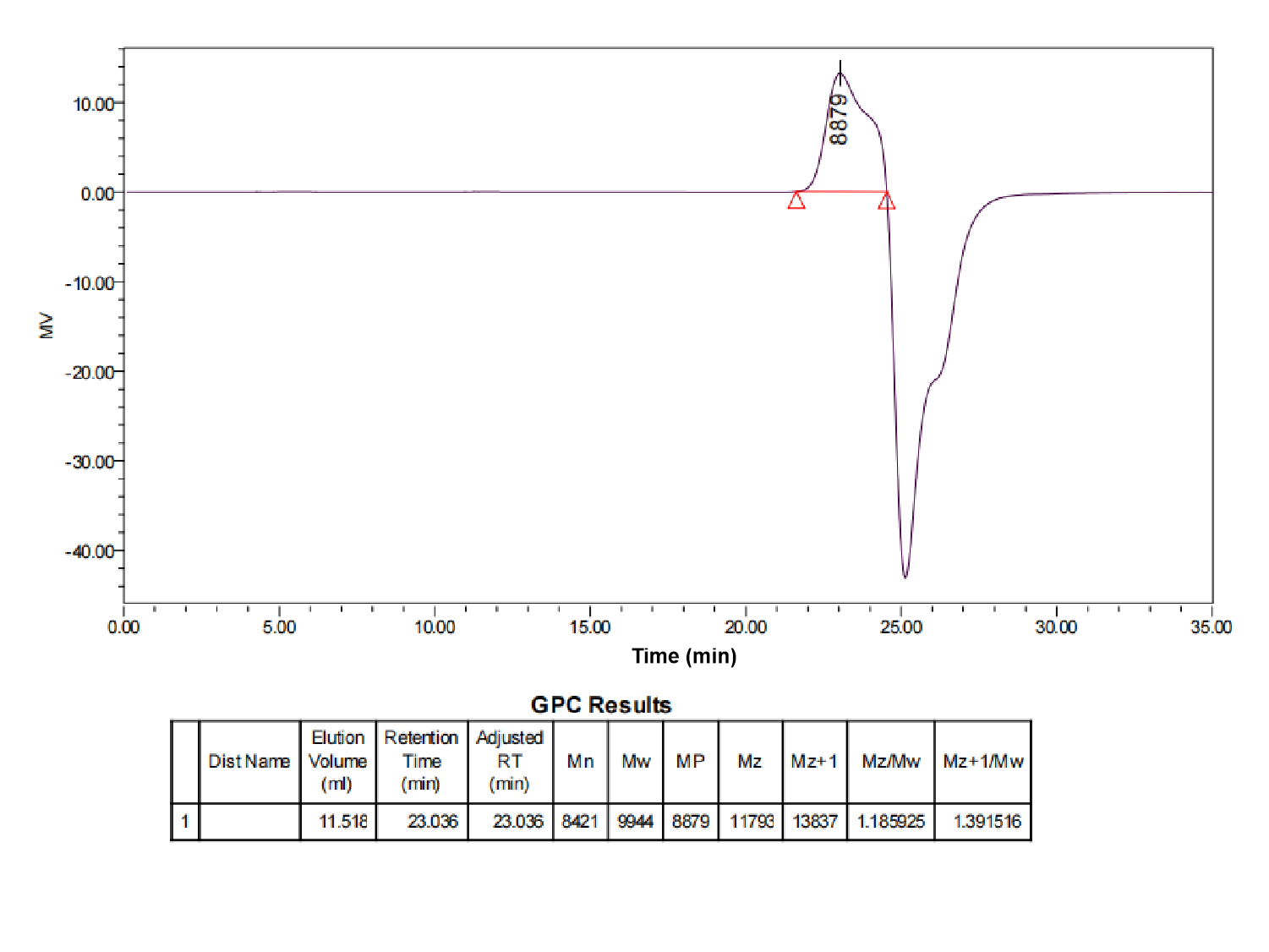


**Figure S31.** GPC chromatogram of F127-D-PLys.


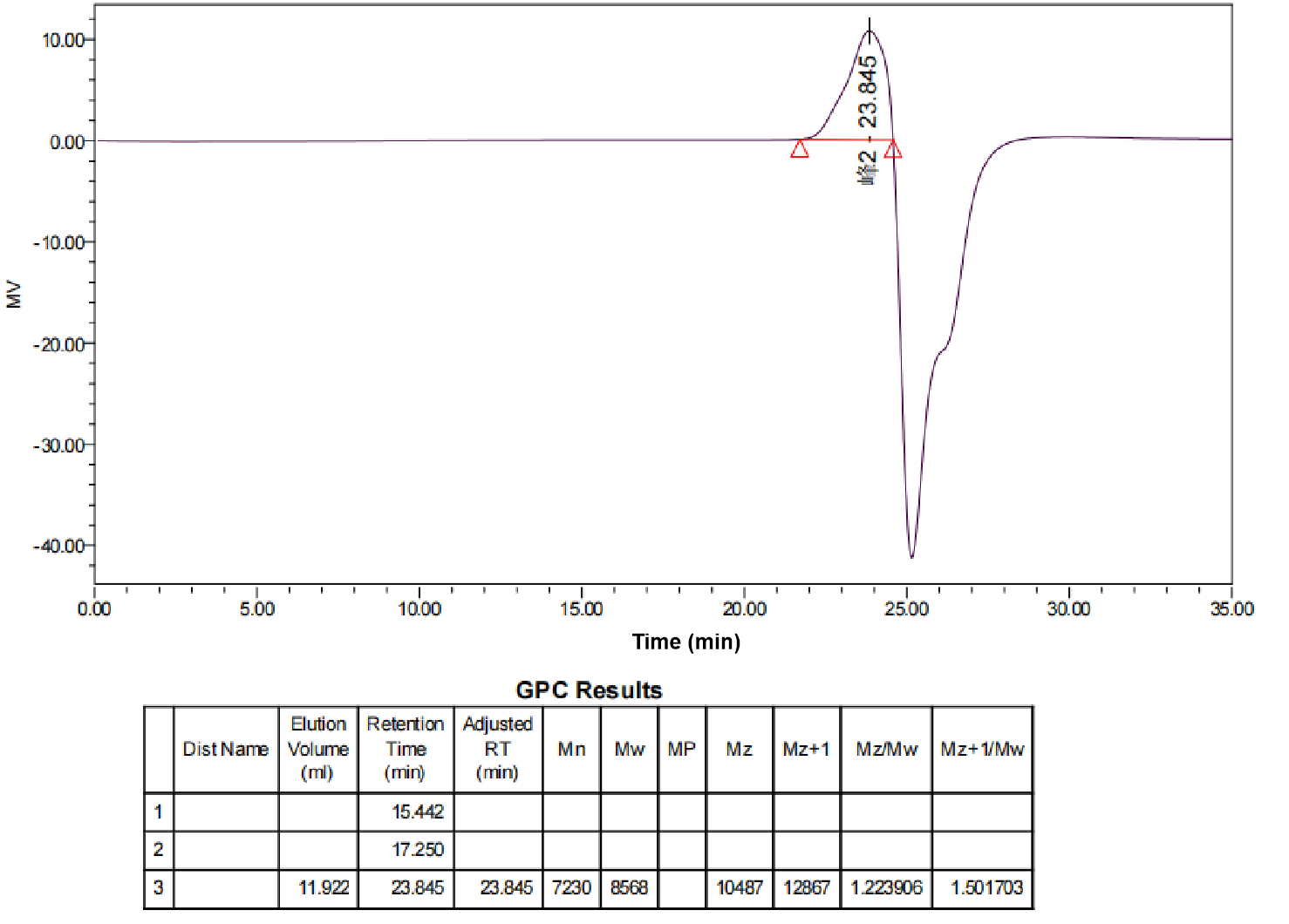


**Figure S32.** GPC chromatogram of F127-L-PGlu.


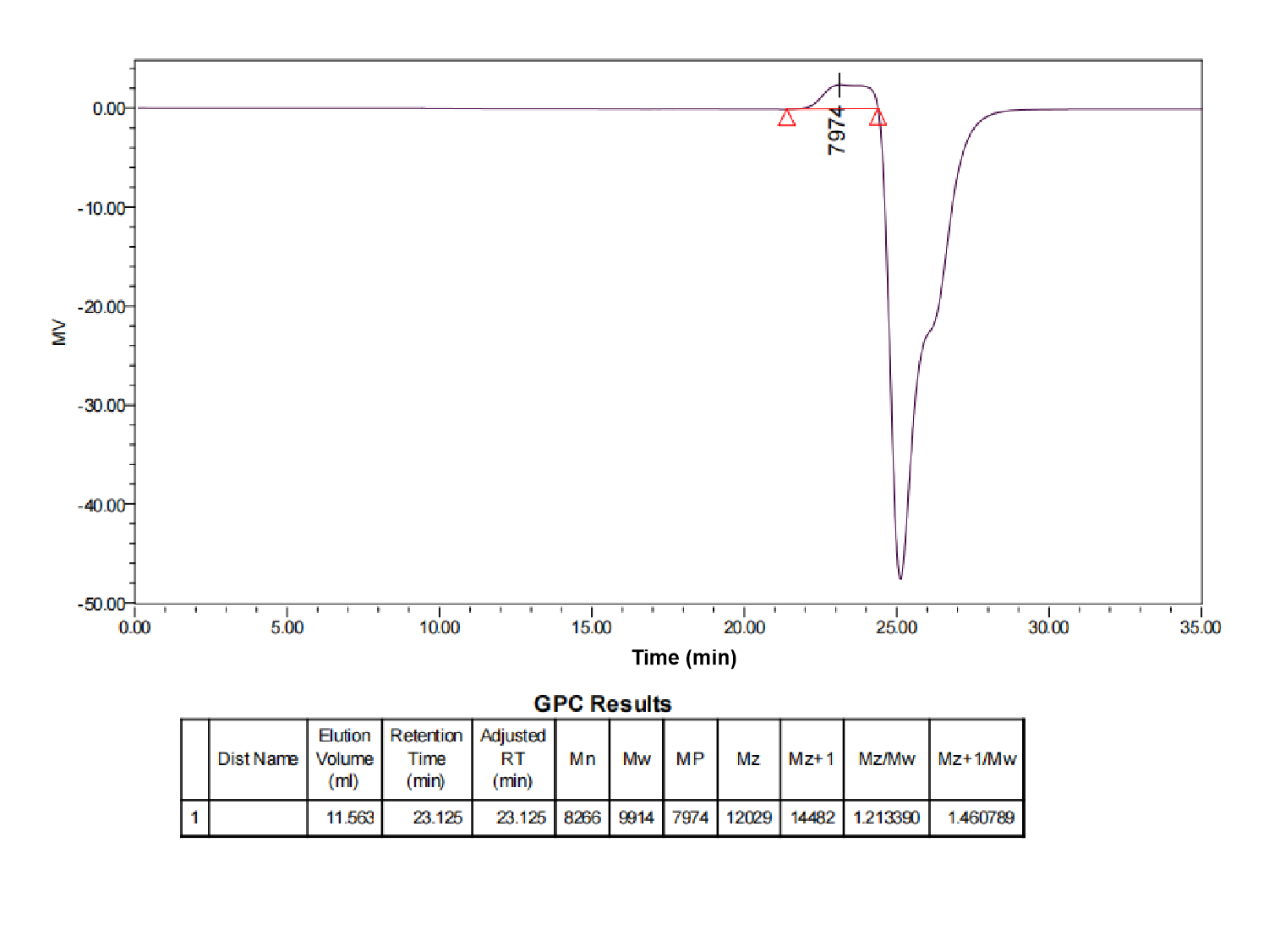


**Figure S33.** GPC chromatogram of F127-D-PGlu.


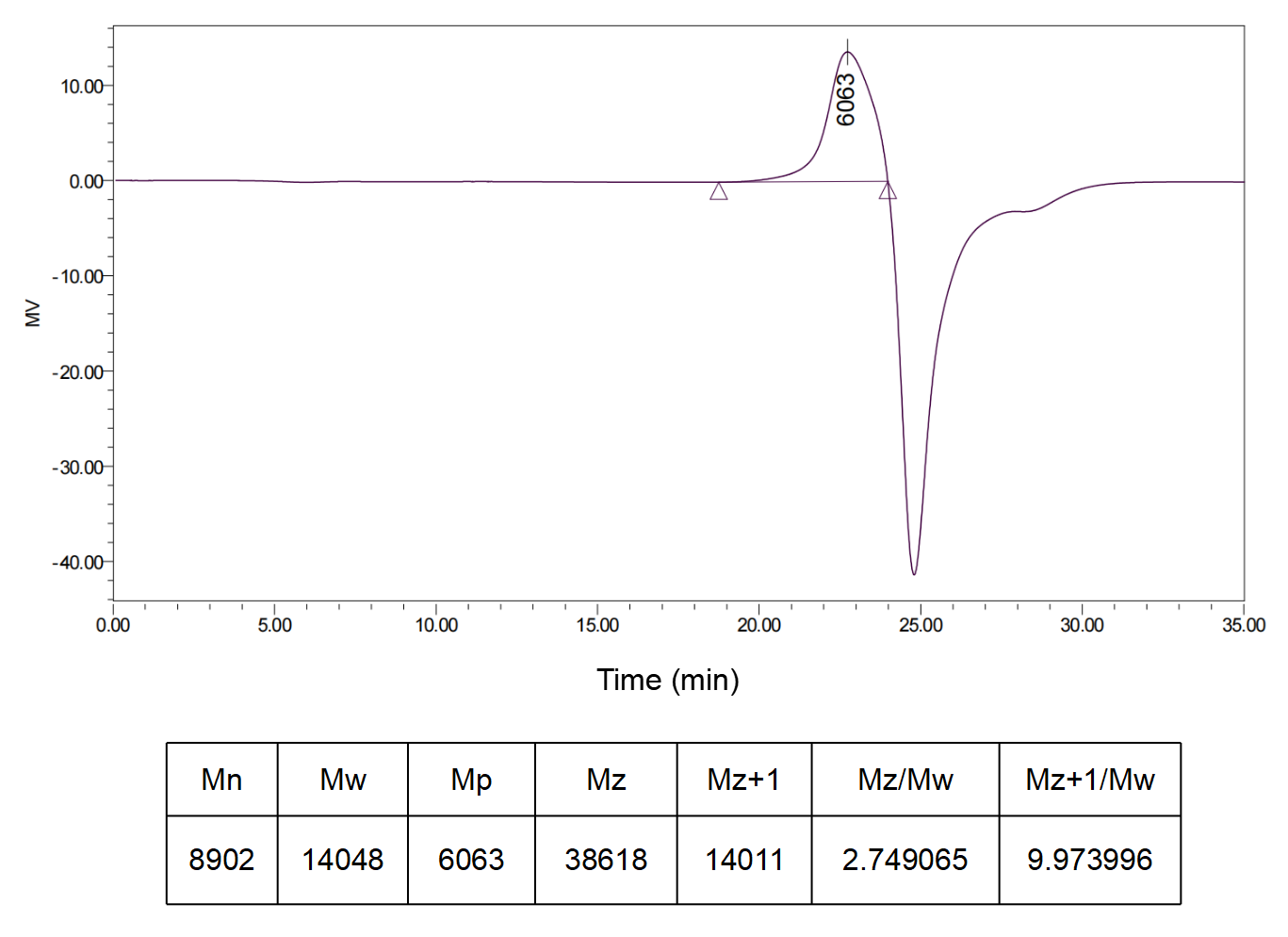


**Figure S34.** F127-L-PLYS. GPC chromatogram of F127-L-PLys.


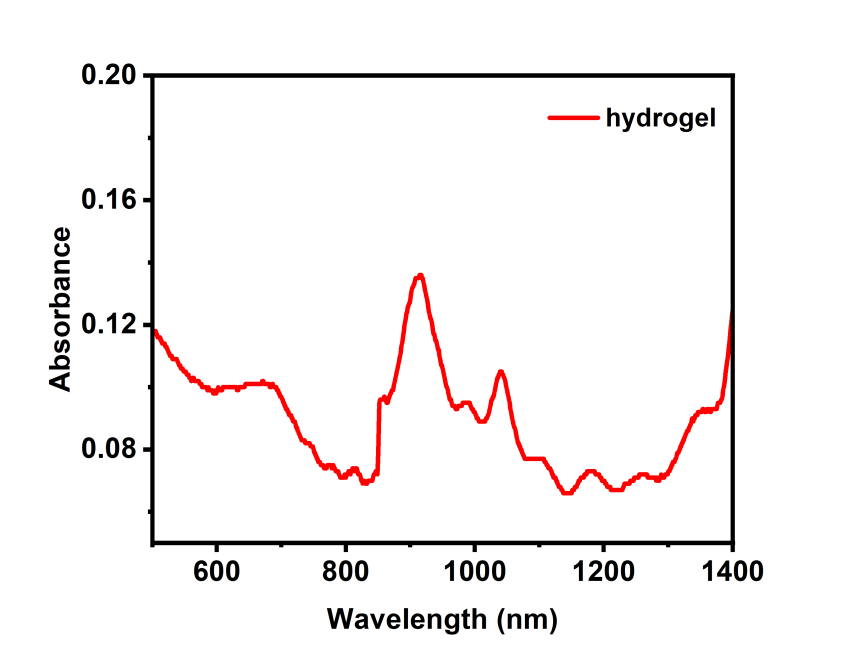


**Figure S35.** UV-vis absorption spectrum of TDI^•-^ D-gel.


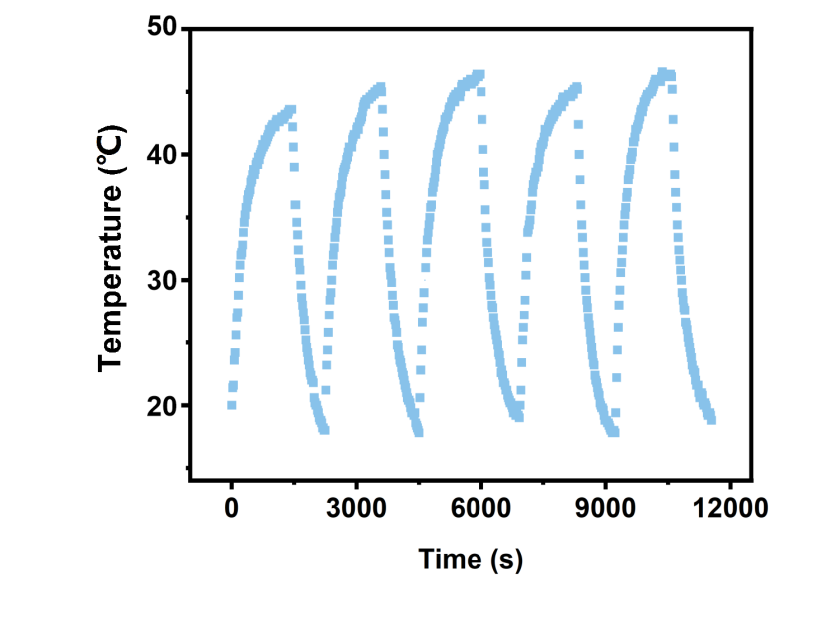


**Figure S36.** Multiple heating-cooling cycles of the TDI^•-^ D-gel (5 mg/mL) under 1064 nm laser irradiation (0.5 W/cm²) in the solid state.

| ****Gel type by polymer composition**** | ****Polymer component(s)**** | ****Gel name**** |
| --- | --- | --- |
| ****Dual-polymer gels**** | F127-D-PGlu | TDI^•-^ D-gel |
|  | F127-D-PLys |  |
|  | F127-L-PGlu | TDI^•-^ L-gel |
|  | F127-L-PLys |  |
| **Single-polymer gels** | F127 | TDI^•-^ gel |
|  | F127-D-PGlu | TDI^•-^ D-gel_(Glu)_ |
|  | F127-D-PLys | TDI^•-^ D-gel_(Lys)_ |
|  | F127-L-PGlu | TDI^•-^ L-gel_(Glu)_ |
|  | F127-L-PLys | TDI^•-^ L-gel_(Lys)_ |

**Table S3.** Classification and nomenclature of hydrogels based on polymer composition.


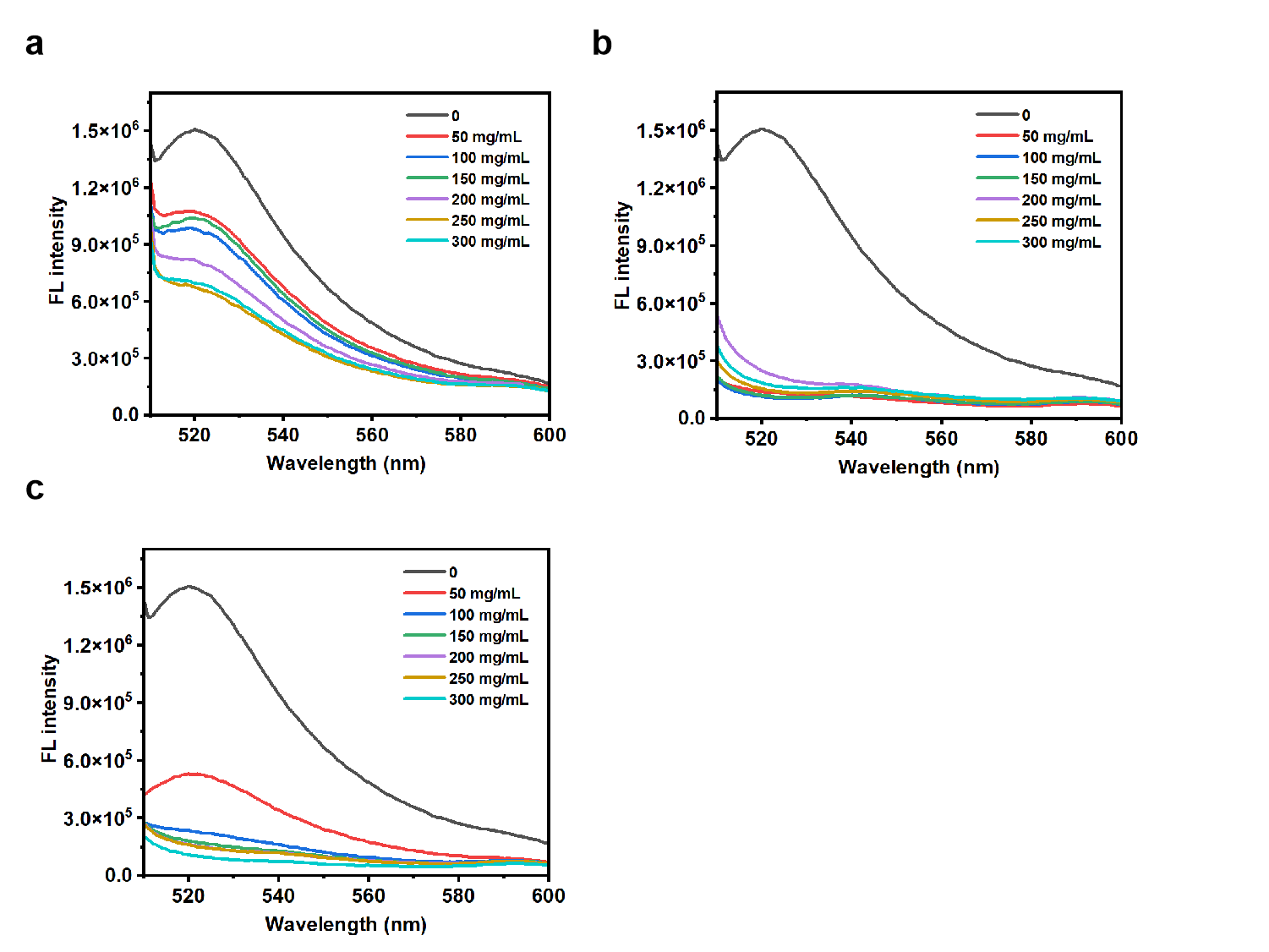


**Figure S37.** Fluorescence spectra of the TDI^•-^ D-gel_(Glu)_ (a), TDI^•-^ D-gel_(Lys)_ (b), and TDI^•-^ D-gel (c) at different concentrations (0-300 mg/mL) after adsorption of BSA-FITC.


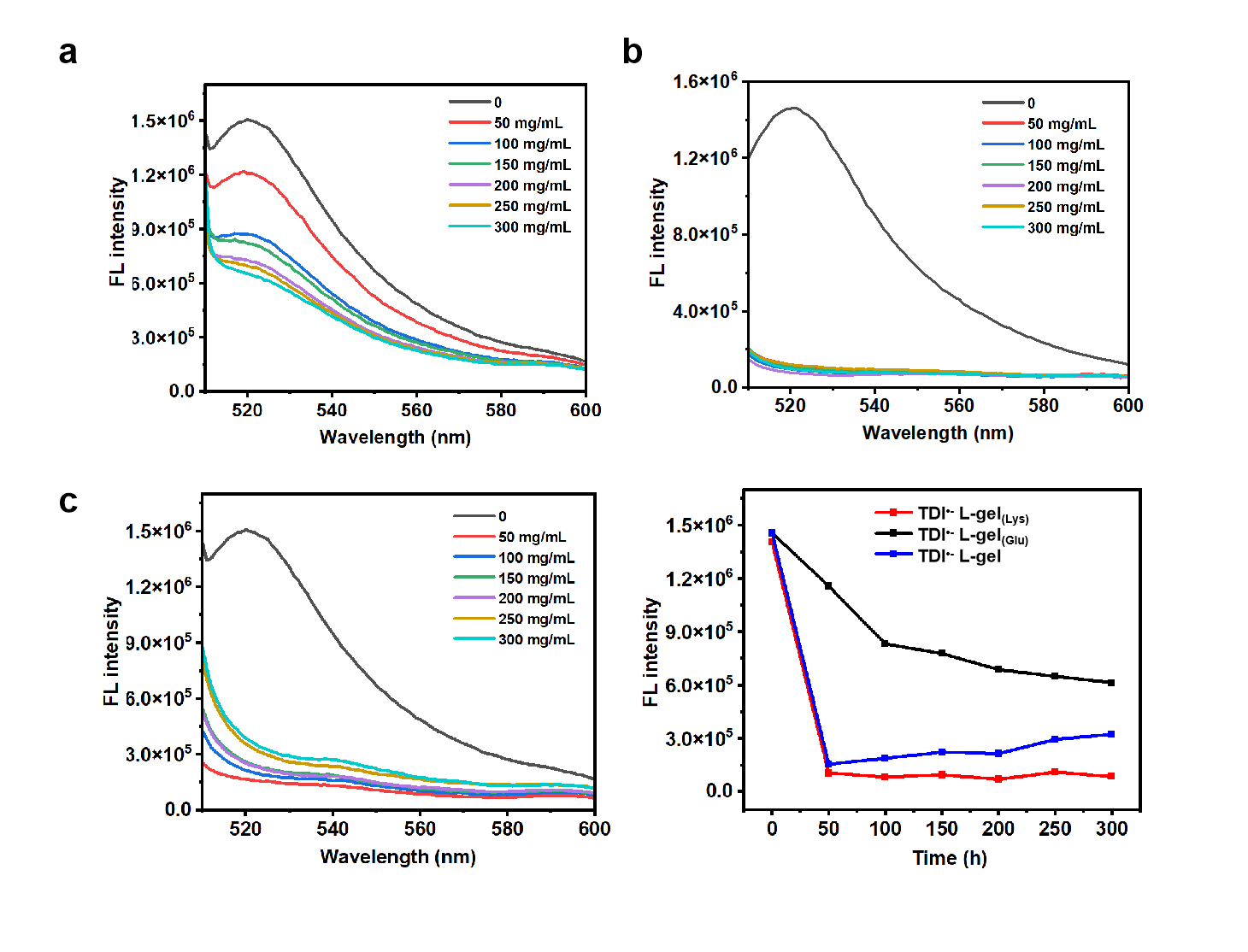


**Figure S38.** Fluorescence spectra of TDI^•-^ L-gel_(Glu)_ (a), TDI^•-^ L-gel_(Lys)_ (b), and TDI^•-^ L-gel (c) at different concentrations (0-300 mg/mL) after adsorption of BSA-FITC. (d) Comparison of the FL intensity changes of TDI^•-^ L-gel_(Glu)_, TDI^•-^ L-gel_(Lys)_ and TDI^•-^ L-gel at 525 nm.


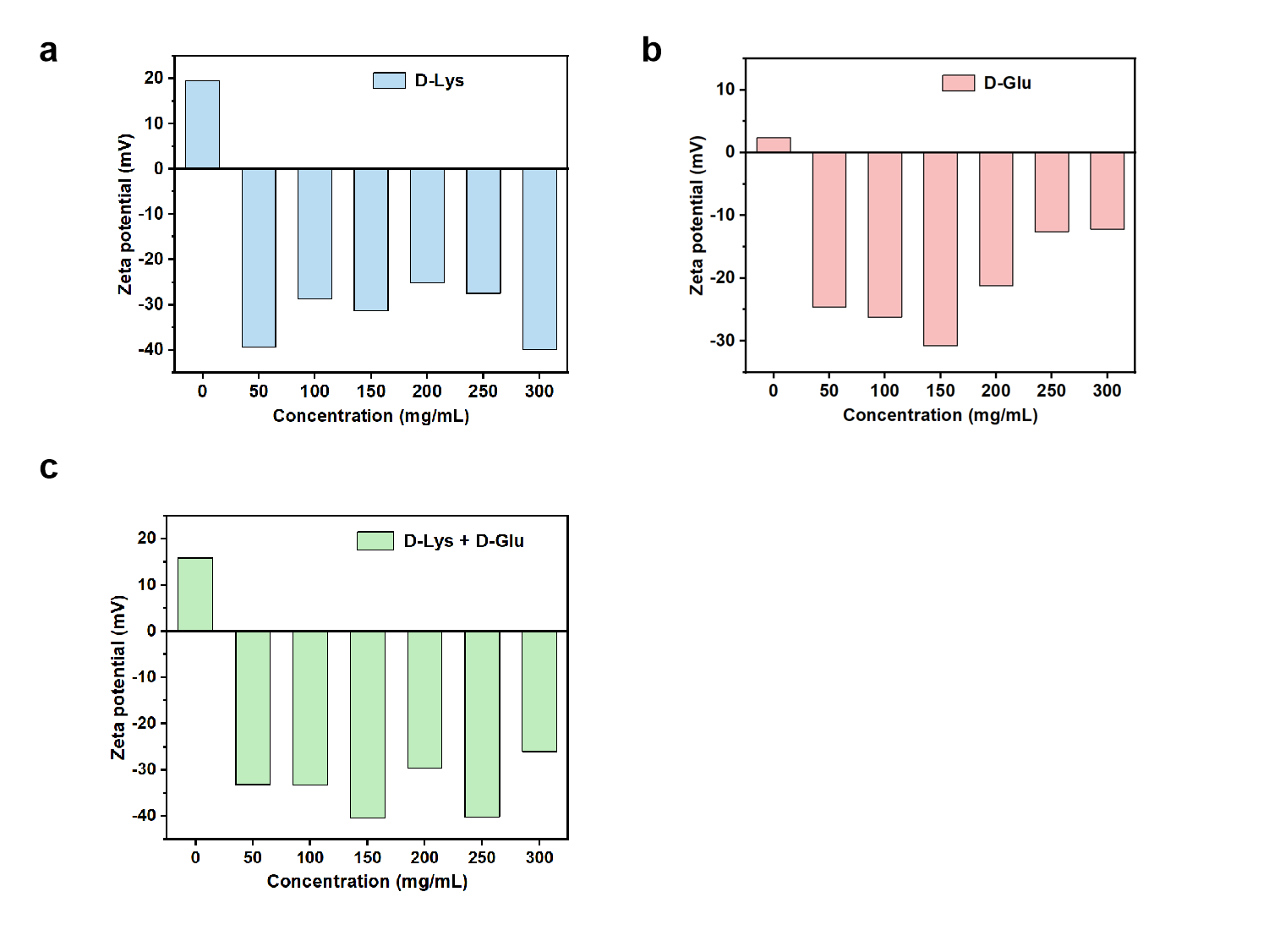


**Figure S39.** Zeta potential of TDI^•-^ D-gel_(Glu)_ (a), TDI^•-^ D-gel_(Lys)_ (b), and TDI^•-^ D-gel (c) at different concentrations (0-300 mg/mL) after adsorption of tumor-associated antigen.


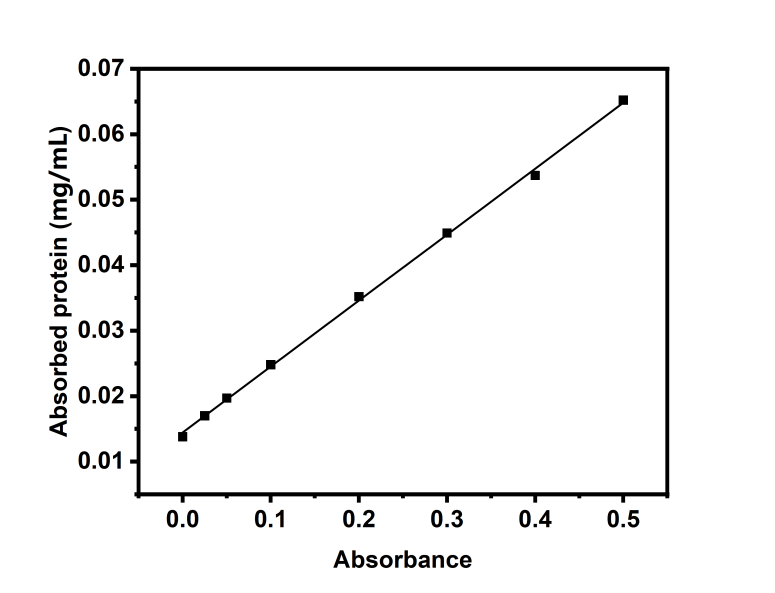


**Figure S40.** Standard curve for tumor-associated antigen detection using the BCA protein assay kit.

**
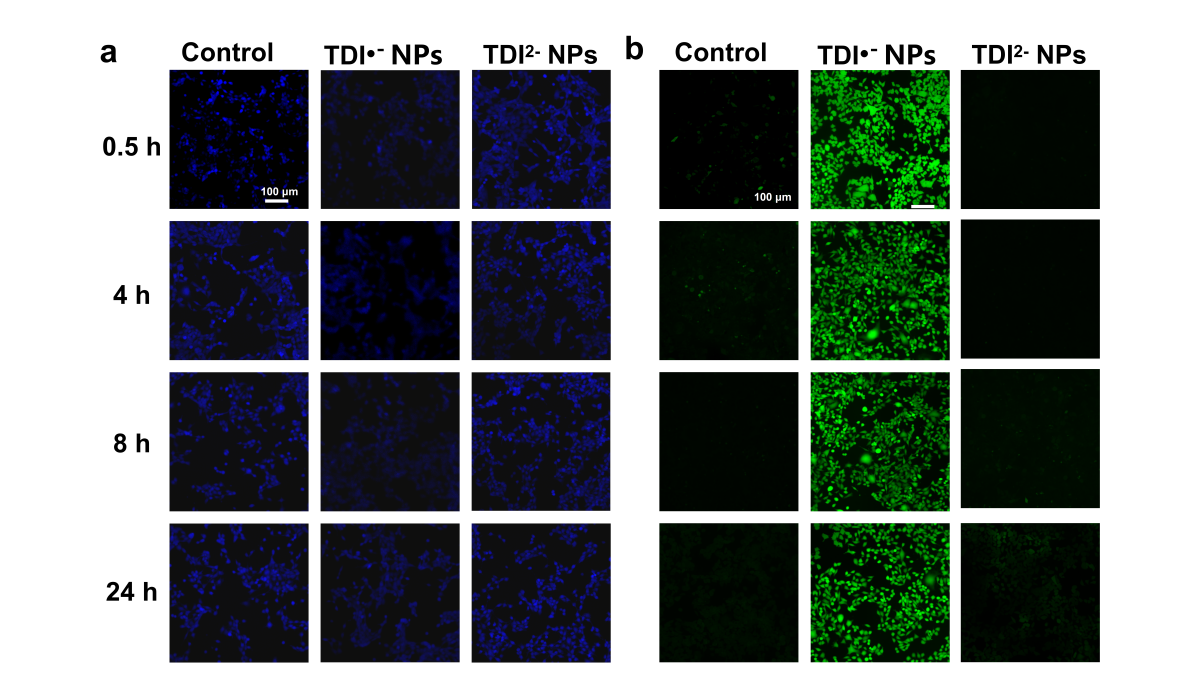
**

**Figure S41.** Time-dependent fluorescence images showing intracellular GSH depletion (a) and ROS generation (b) in 4T1 cells treated with TDI^•-^ NPs and TDI^2-^ NPs over 24 h. These data correspond to the non-gelled NP control and are complementary to the hydrogel-based results shown in Figure 3a-d. Scale bar: 100 μm.

**
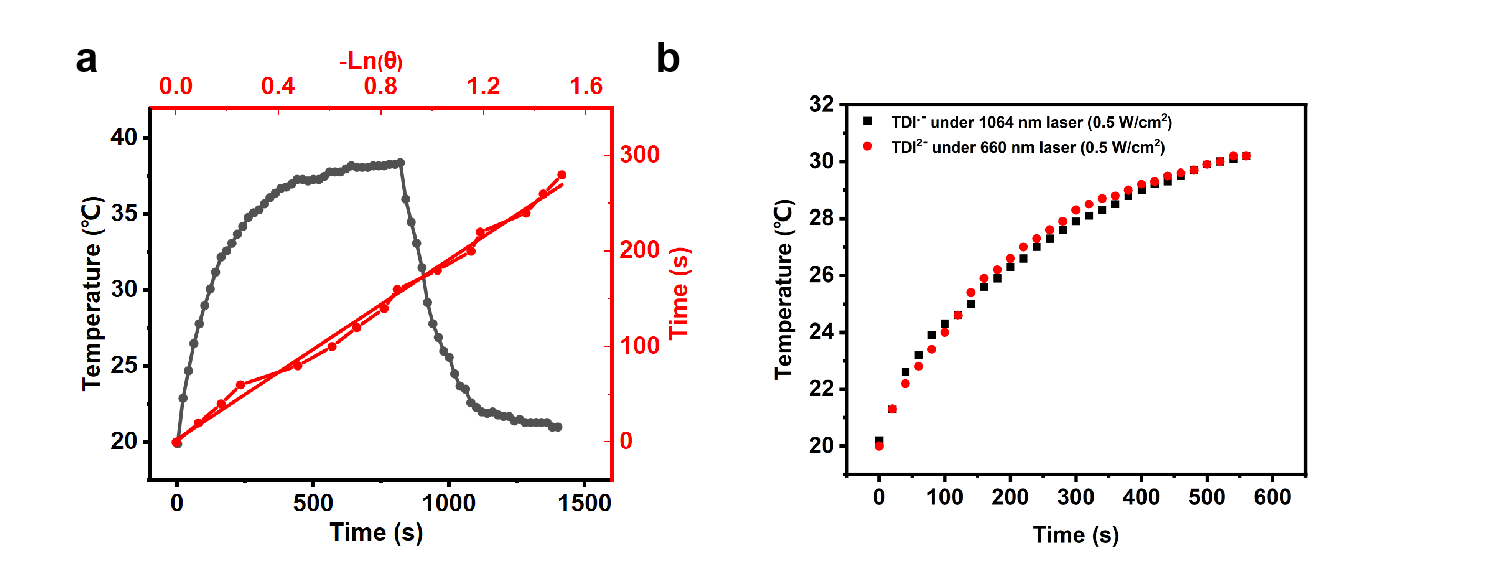
**

**Figure S42.** (a) Heating-cooling curves of TDI²⁻ gel under 660 nm laser irradiation (1 W/cm²). (b) Comparison of photothermal heating curves between TDI^•-^ gel (1064 nm laser, 0.5 W/cm²) and TDI²⁻ gel (660 nm laser, 0.5 W/cm²).

**
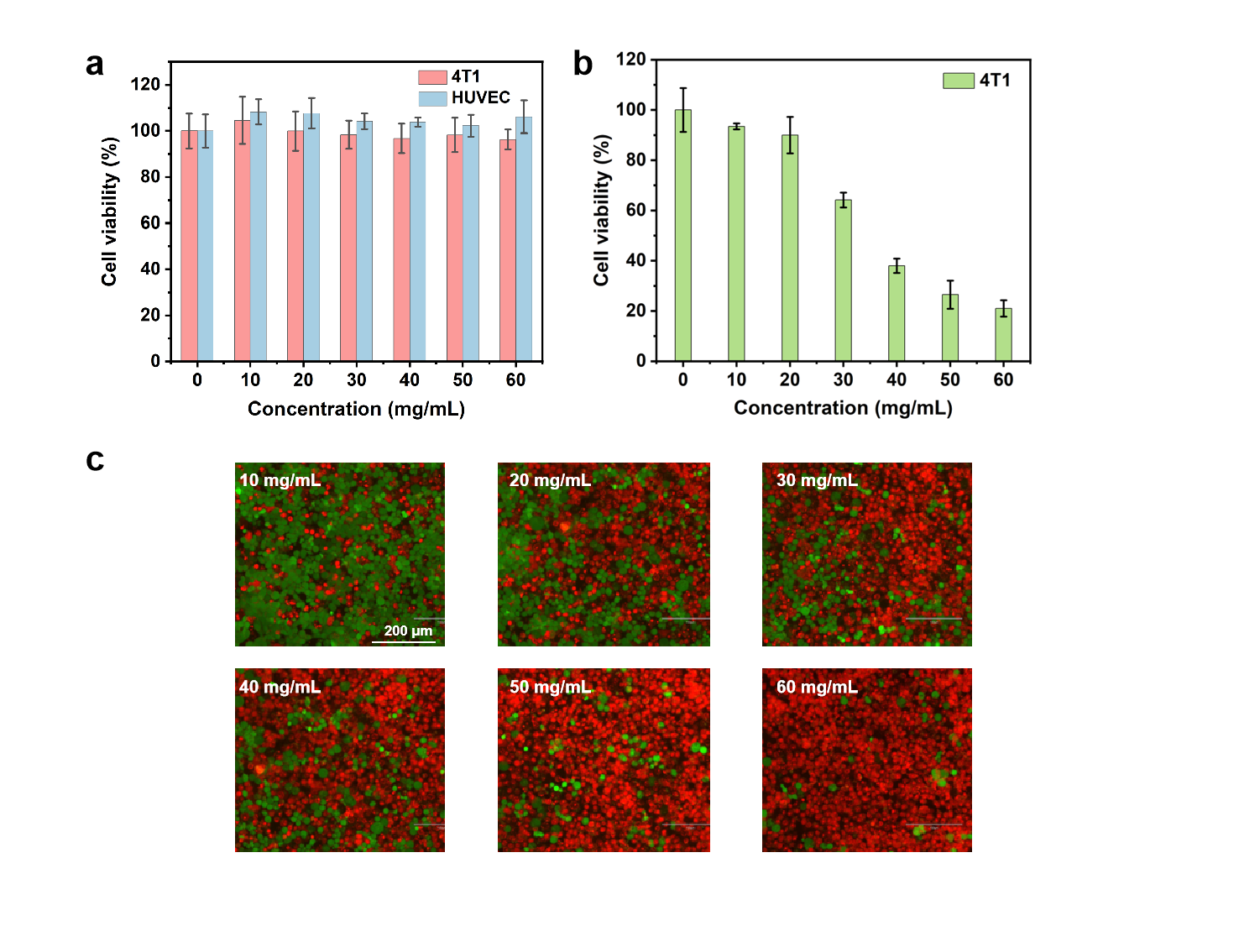
**

**Figure S43.** (a) Cell viability of 4T1 and HUVEC cells treated with TDI^•-^ D-gel as measured by CCK-8 assay. (b) Cell viability of cells treated with TDI^•-^ D-gel followed by laser irradiation (1064 nm, 0.5 W/cm², 5 min) as measured by CCK-8 assay. (c) Live/dead fluorescence imaging of cells incubated with TDI^•-^ D-gel at different concentrations (10-60 mg/mL). Scale bar: 200 μm.


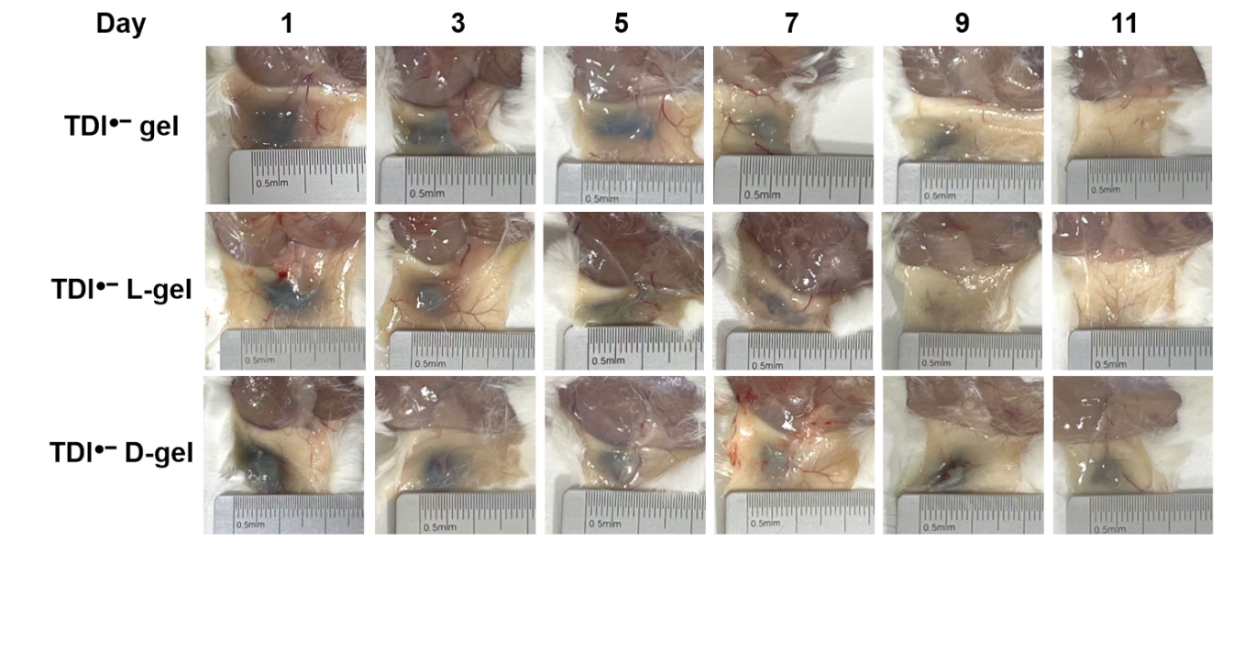


**Figure S44.** *In vivo* retention behavior of different chiral hydrogels in subcutaneous mouse model.


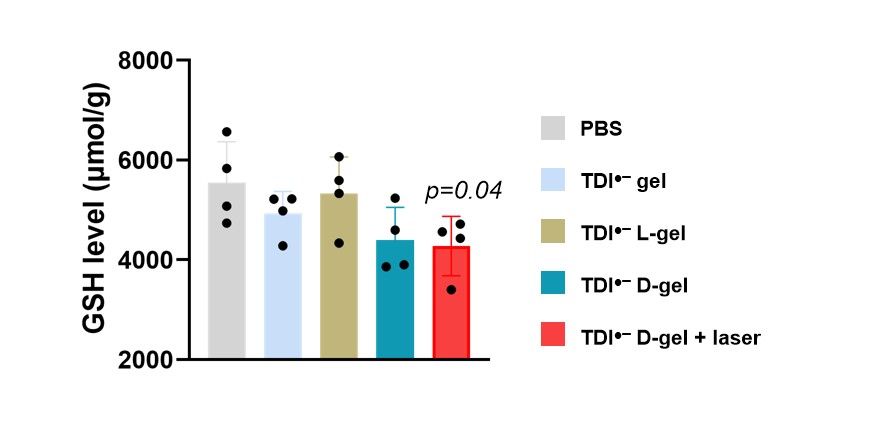


**Figure S45.** Tumor GSH levels over 7 days under different treatments.


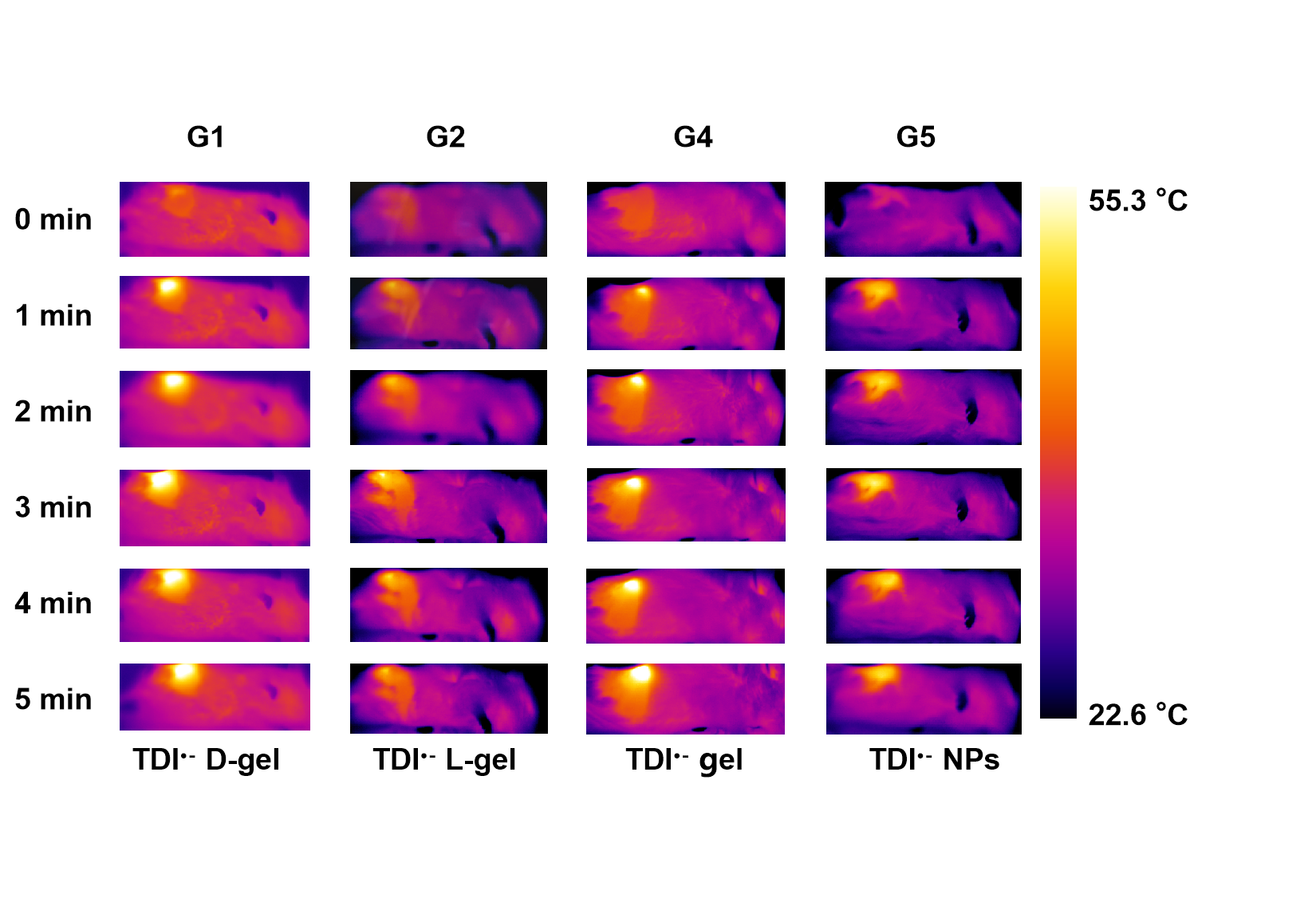


**Figure S46.** Photothermal images of 4T1 tumor-bearing mice subcutaneous injected with TDI^•-^ D-gel (40 μL), TDI^•-^ L-gel (40 μL), TDI^•-^ gel (40 μL) and PBS under 1064 nm laser irradiation (0.5 W/cm^2^)


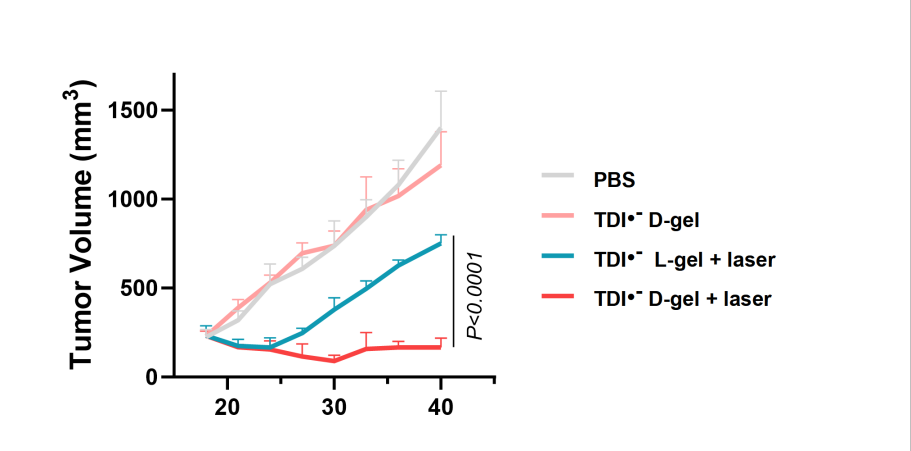
.

**Figure S47.** Primary tumor growth curves of 4T1 tumor-bearing mice receiving different treatments. Tumor volumes were monitored during the treatment period and are presented as mean ± SD (n = 5 mice per group). Statistical significance was analyzed by two-way ANOVA with Tukey’s post hoc test.


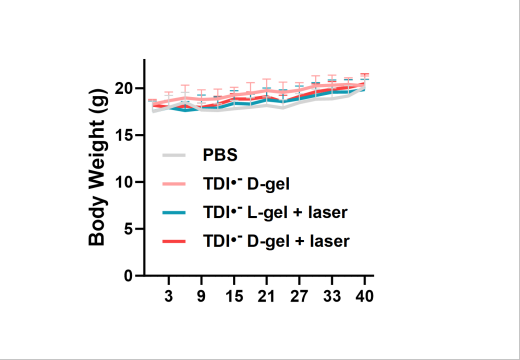


**Figure S48.** Body weight changes of 4T1 tumor-bearing mice during treatment with different formulations. Data are presented as mean ± SD (n = 5 mice per group). Statistical analysis was performed by two-way ANOVA with Tukey’s post hoc test; no significant treatment-related body weight loss was observed.


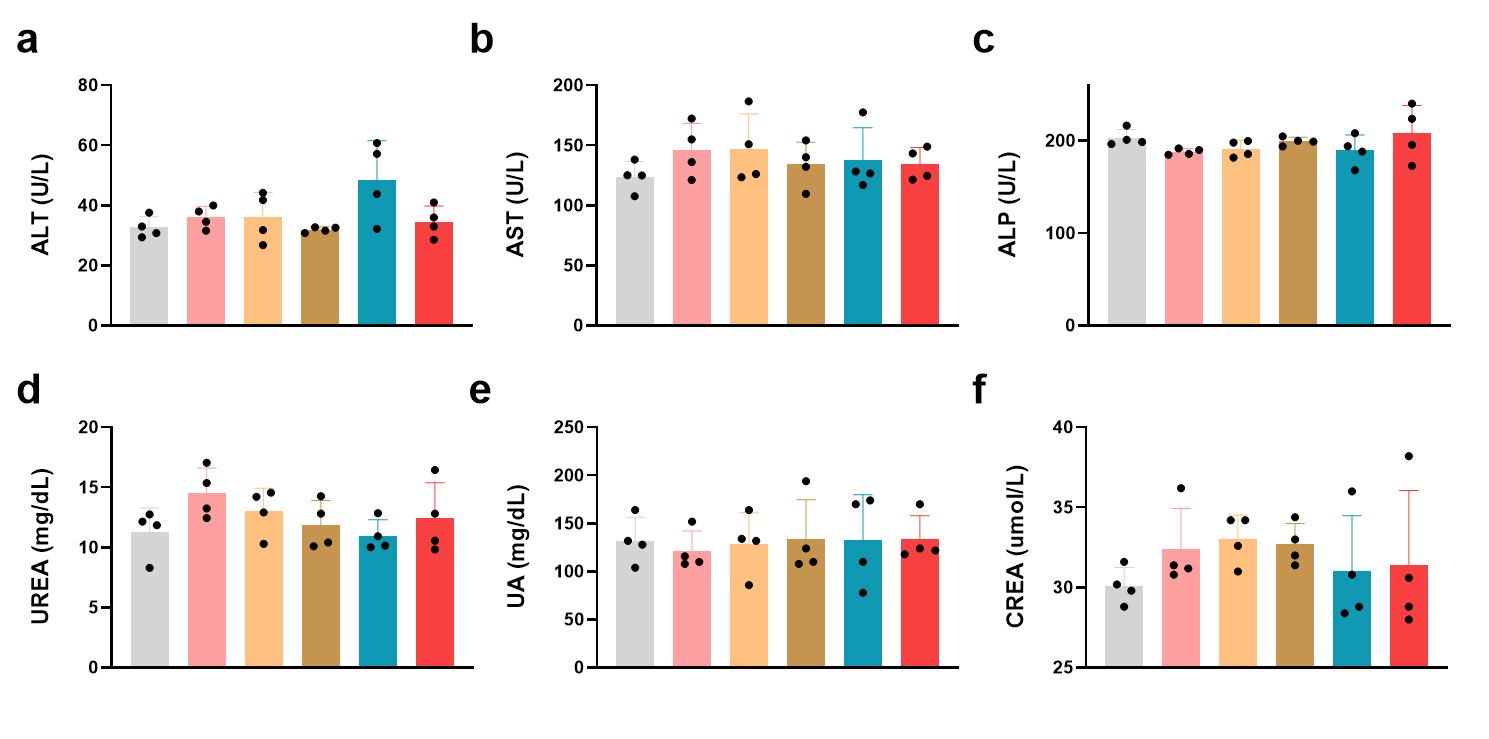


**Figure S49.** Blood biochemical analyses of TDI•- D-gel. Serum biochemistry data (ALT: alanine aminotransferase, AST: aspartate aminotransferase, ALP: Alkaline Phosphatase, UREA: Carbamide, UA: urine acid, and CREA: Creatinine). Complete blood counts.


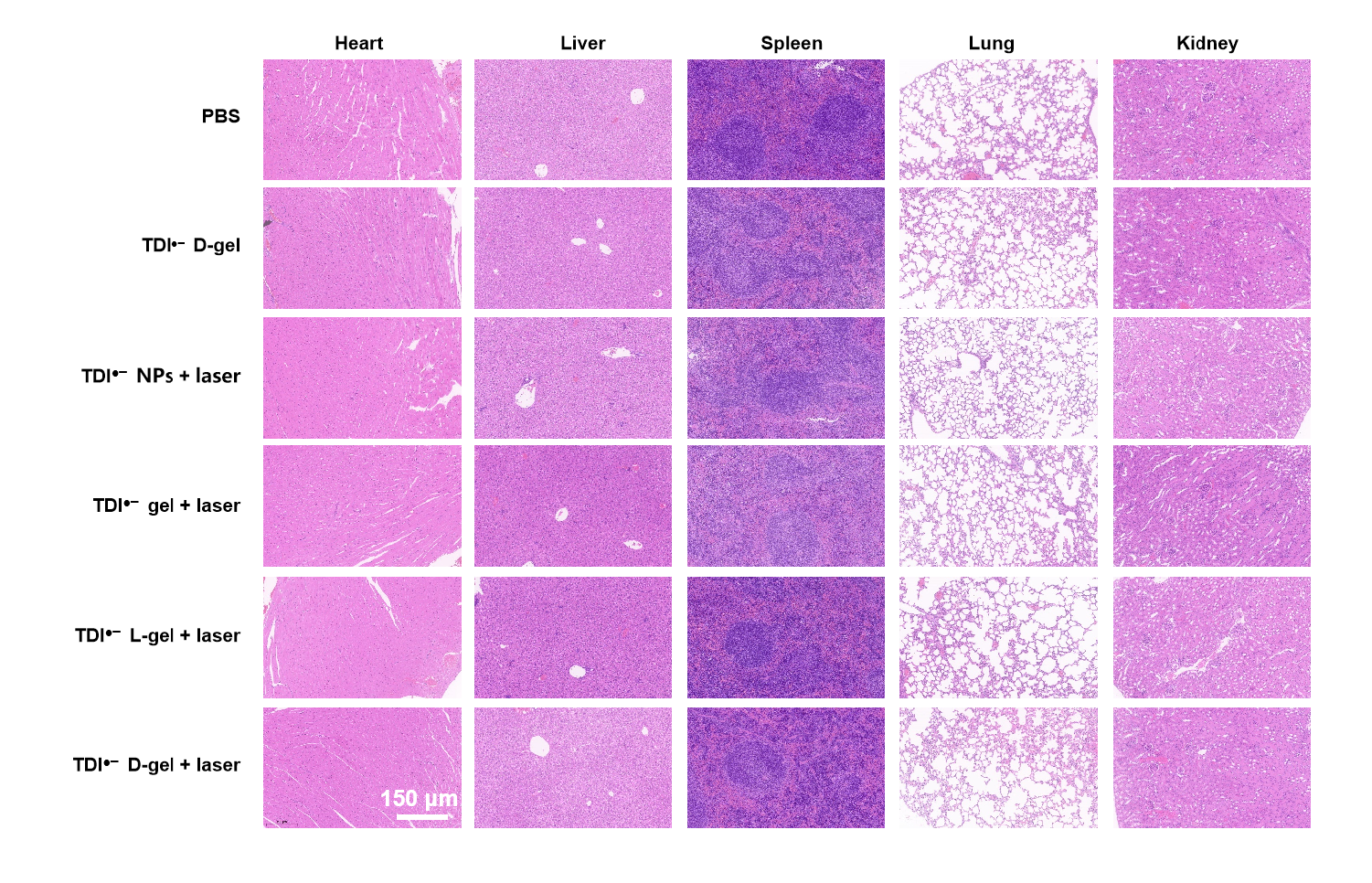


**Figure S50.** H&E staining of major organs from differently treated mice harvested on day 40. Scale bar: 150 μm.


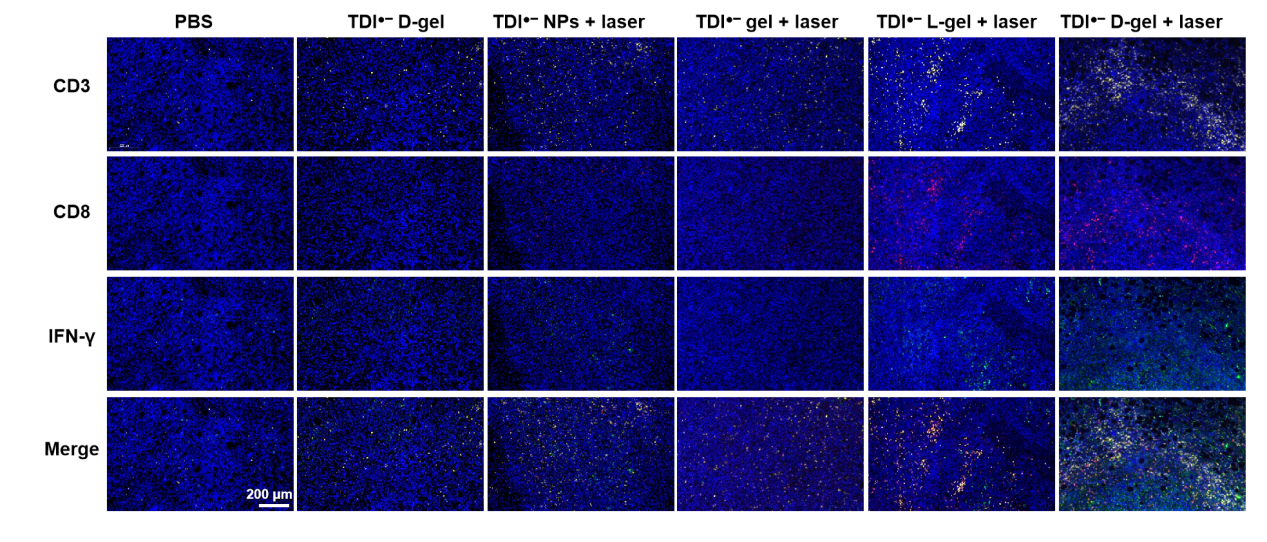


**Figure S51.** Immunofluorescence images of T cell infiltration in tumor tissues following various treatment regimens. Scale bar: 200 μm.


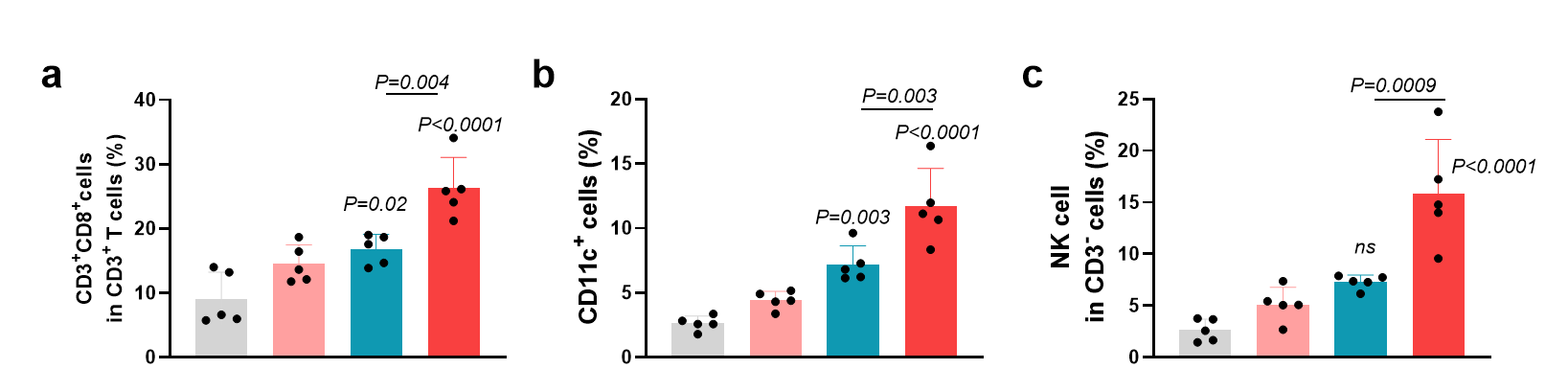


**Figure S52.** Flow cytometry analysis of NK cells in tumors following different treatments.


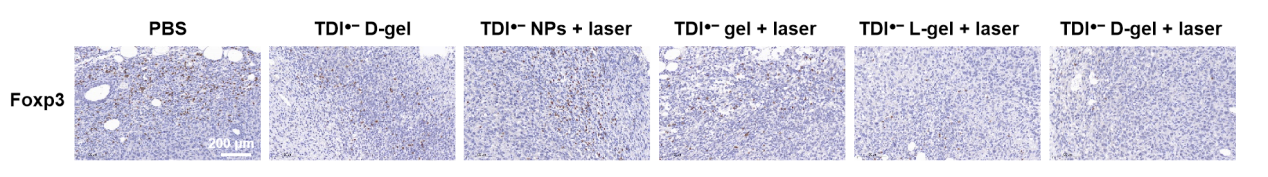


**Figure S53.** Immunofluorescence images of Foxp3 expression in tumor tissues following various treatment regimens. Scale bar: 200 μm.

**Reference**

[44] D. Ma, H. B. Zhang, D. H. Chen, L. M. Zhang, “Novel supramolecular gelation route to in situ entrapment and sustained delivery of plasmid DNA” *J. Colloid Interface Sci.* 364 (2011): 566-573. https://doi.org/10.1016/j.jcis.2011.08.051
